# Sequential drug treatment targeting cell cycle and cell fate regulatory programs blocks non-genetic cancer evolution in acute lymphoblastic leukemia

**DOI:** 10.1186/s13059-024-03260-4

**Published:** 2024-05-31

**Authors:** Alena Malyukova, Mari Lahnalampi, Ton Falqués-Costa, Petri Pölönen, Mikko Sipola, Juha Mehtonen, Susanna Teppo, Karen Akopyan, Johanna Viiliainen, Olli Lohi, Anna K. Hagström-Andersson, Merja Heinäniemi, Olle Sangfelt

**Affiliations:** 1 Department of Cell and Molecular Biology, Karolinska Institutet, Biomedicum, Solnavägen 9, 171 77 Stockholm, Sweden; 2 The Institute of Biomedicine, School of Medicine, University of Eastern Finland, Kuopio, Finland; 3 Division of Clinical Genetics, Department of Laboratory Medicine, Lund University, Lund, Sweden; 4 Tampere Center for Child, Adolescent and Maternal Health Research, Faculty of Medicine and Health Technology, Tampere University and Tays Cancer Center, Tampere University Hospital, Tampere, Finland

**Keywords:** Single-cell multiomics, WEE1, AZD1775, RUNX1, KMT2A-r, Pre-BCR, BCL6, B-ALL, Leukemia, Chromatin state, Cell state transition

## Abstract

**Background:**

Targeted therapies exploiting vulnerabilities of cancer cells hold promise for improving patient outcome and reducing side-effects of chemotherapy. However, efficacy of precision therapies is limited in part because of tumor cell heterogeneity. A better mechanistic understanding of how drug effect is linked to cancer cell state diversity is crucial for identifying effective combination therapies that can prevent disease recurrence.

**Results:**

Here, we characterize the effect of G2/M checkpoint inhibition in acute lymphoblastic leukemia (ALL) and demonstrate that WEE1 targeted therapy impinges on cell fate decision regulatory circuits. We find the highest inhibition of recovery of proliferation in ALL cells with KMT2A-rearrangements. Single-cell RNA-seq and ATAC-seq of RS4;11 cells harboring KMT2A::AFF1, treated with the WEE1 inhibitor AZD1775, reveal diversification of cell states, with a fraction of cells exhibiting strong activation of p53-driven processes linked to apoptosis and senescence, and disruption of a core KMT2A-RUNX1-MYC regulatory network. In this cell state diversification induced by WEE1 inhibition, a subpopulation transitions to a drug tolerant cell state characterized by activation of transcription factors regulating pre-B cell fate, lipid metabolism, and pre-BCR signaling in a reversible manner. Sequential treatment with BCR-signaling inhibitors dasatinib, ibrutinib, or perturbing metabolism by fatostatin or AZD2014 effectively counteracts drug tolerance by inducing cell death and repressing stemness markers.

**Conclusions:**

Collectively, our findings provide new insights into the tight connectivity of gene regulatory programs associated with cell cycle and cell fate regulation, and a rationale for sequential administration of WEE1 inhibitors with low toxicity inhibitors of pre-BCR signaling or metabolism.

**Supplementary Information:**

The online version contains supplementary material available at 10.1186/s13059-024-03260-4.

## Background

The WEE1 checkpoint kinase is a key regulator of the G2/M and G1/S transitions during cell division cycle [1, 2] through inhibitory phosphorylation of cyclin-dependent kinases 1/2 (CDK1/2) [3, 4]. WEE1 supports genome integrity by suppressing excessive origin firing during DNA replication and premature entry into mitosis [5–7]. Consequently, inhibition of WEE1 removes the negative phosphorylation on CDK1/2 (in concert with CDC25) resulting in deregulation of CDKs, increased origin firing and replication fork degradation leading to fork collapse, DNA damage, and unscheduled mitosis [1, 8–10]. Recent studies show that WEE1 inhibition by AZD1775 (Adavosertib), a potent and specific small-molecule inhibitor, alone or in combination with DNA damaging agents effectively kills cancer cells of various origins [8, 11, 12]. The targeting of DNA damage response-related proteins while also challenging the cell with genotoxic agents is a widely adopted concept in cancer therapy [13]. However, clinical application is restrained by a high rate of toxicity in combination with chemotherapy [13–16].

Acute lymphoblastic leukemia (ALL) is the most common type of childhood cancer, typically of B-cell lineage. Approximately 75% of B-ALL cases contain chromosomal rearrangements involving alteration of lineage-specific transcription factors (TFs) that are critical regulators of B-cell development and serve as prognostic markers underlying clinical responses. Mixed lineage leukemia (MLL) refers to chromosomal translocations involving the gene *KMT2A* which encodes histone lysine N-methyltransferase 2, an important regulator of epigenetic maintenance of stem cell gene transcription through activating histone modification at target genomic loci [17]. Despite improved treatment protocols with higher response rates and better outcomes for children with ALL, KMT2A-r patients have poor prognosis due to frequent relapses upon conventional chemotherapy [18, 19]. However, these leukemias typically harbor very few additional mutations that could explain drug resistance [20]. Recently, single-cell genomics-approaches have provided new insight into the aberrant gene regulatory programs that cause cell state instability, and hence diversification of cell states at cell fate decision points even in isogenic cell populations in response to treatment [21]. Such non-genetic heterogeneity has emerged as a central mechanism of drug resistance in cancer, including in leukemia [22–24]. A network of regulatory interactions exerted by TFs poises cells at fate decision points, primed for diversification into cell phenotypes with differential drug sensitivity [25, 26]. Specifically, increased drug tolerance in leukemia and other cancers [21] has been attributed to “stemness” properties, due to shift into more immature (stem-like) states—a phenotype switch governed by gene regulatory networks as opposed to resulting from genomic mutations [21, 24, 27–29].

Chromosomal rearrangements of the *KMT2A* gene can produce many different chimeric TFs through in-frame fusions with various partner genes [30], in ALL most commonly resulting in KMT2A::AFF1 (AF4) and KMT2A::MLLT1 (ENL) fusions through t(4;11) and t(11;19) rearrangements, respectively. These fusion proteins lack the C-terminal SET domain which normally confers the KMT2A multiprotein complex histone H3K4 methyltransferase activity. KMT2A fusion proteins instead associate with the super elongation complex, resulting in differentiation arrest at the early hematopoietic progenitor state due to increased levels of activating H3K79 methylation by DOT1L at genomic loci such as *HOXA9* and *MEIS1*, which are associated with leukemic transformation [18, 31]. However, KMT2A fusions also compromise the S-phase checkpoint by abrogating stabilization of wild-type KMT2A upon DNA damage, thereby preventing trimethylation of H3K4 and causing aberrant loading of CDC45 at late replication origins [32]. Recent work further indicates that other hematopoietic master regulators of stemness and development, notably RUNX proteins, also participate in cell cycle checkpoint control and DNA repair independent of their TF activities [33–36]. The impact of KMT2A fusion proteins at the convergence of two pivotal regulatory pathways, cell cycle checkpoint and cell fate control, represents a potential vulnerability; however, diversification of cell states upon targeted therapy may induce tolerance in distinct subpopulations, whose control would require combinatorial and sequential treatment.

Cancer cell responses to drug perturbations are often assayed using simple phenotypic readouts, such as proliferation or cell death. However, these end-point assays are insufficient to uncover cellular mechanisms that allow some cells to survive and that arise only in response to treatment within a subpopulation. Thus, distinguishing between selection of pre-existing drug-resistant cells and drug tolerance through drug-induced mechanisms is important to predict long-term drug efficacy. Importantly, targeted therapy that impinges on core regulatory circuits affects cell fate decisions which are poised to cause cell fate diversification. The latter generates non-genetic heterogeneity that has emerged as a major driver of treatment resistance [21, 37]. In the present study, we characterize in detail the genome-wide response and cell state dynamics and diversification upon treatment with cell cycle checkpoint-targeted drugs in leukemic cells. We used single-cell resolution multi-omics and demonstrated that perturbing the cell cycle regulation directly impacts cell fate regulatory circuits and cell fate decision.

## Results

### WEE1 inhibitor AZD1775 results in prolonged growth inhibition selectively in KMT2A-r B-ALLs

**Fig. 1:**
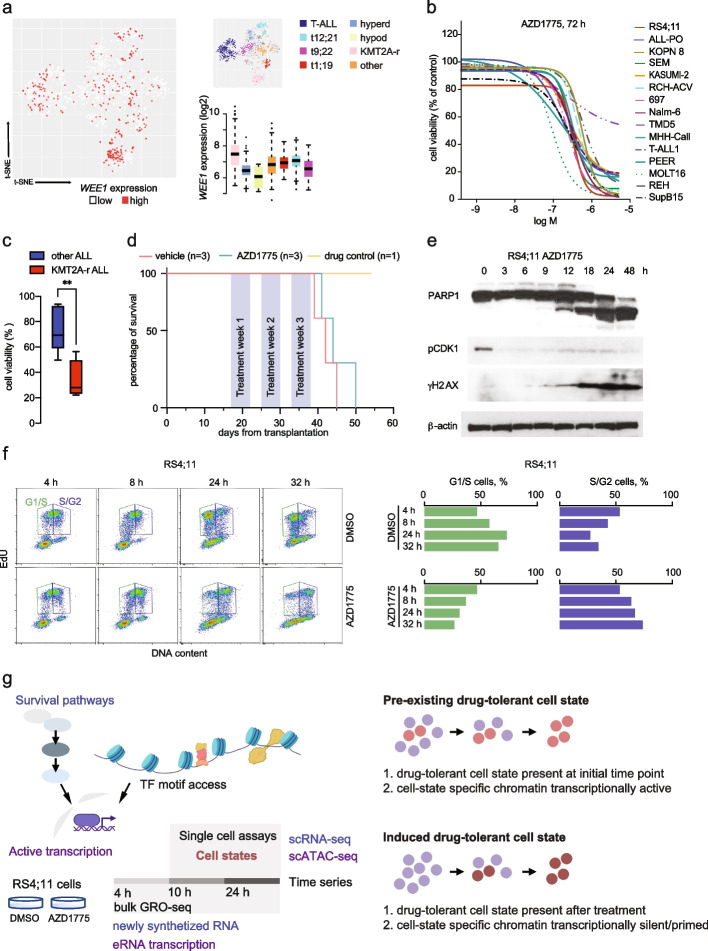
Characterization of AZD1775 response and *WEE1* expression in patient cells and in vitro models. **a** *WEE1* expression in primary ALL samples in HEMAP. tSNE-map: low (in white), high (in red) mRNA level. Boxplot: log2 normalized gene expression across subtypes. Wilcoxon test FDR: Hyperdiploid 6.1^-22^, t(9;22) 1.8^-18^, t(12;21) 6.0^-6^, t(1;19) 5.6^-6^, Hypodiploid 0.009, B-other 3.4^-11^. **b** AZD1775 dose response (relative to DMSO control) assessed by Alamar Blue assay in T-ALL and B-ALL cell lines. Percentage of viable cells at 72 h with increasing concentrations is shown. **c** Recovery of proliferation following removal of AZD1775 (relative to DMSO) analyzed by Alamar Blue assay. Cells were treated for 3 days (AZD1775 GI50 for each cell line, see Additional file 1: Fig. S1b) and allowed to recover for 14 days without the drug. Box plot comparing recovery in KMT2A-r and non-KMT2A-r ALL cell lines (TCF3-PBX1, ETV6-RUNX1, TCF3-PDGFRB subtypes) is shown. ** denotes *p* = 0.0094 determined using two-tailed *t*-test. Data are represented as mean ± SD. **d** Kaplan–Meier survival curves of the MLL-7 patient-derived KMT2A-r ALL treated with AZD1775 in NSG mice. **e** Western blot analysis of the AZD1775 response dynamics in KMT2A-r RS4;11 cells. Whole cell lysates were immunoblotted with specific antibodies; Cleaved PARP1 (cell death), phosphorylation of CDK1-Y15 (WEE1 inhibition), and phosphorylation of γH2AX-S139 (DNA damage). β-actin: loading control. **f** Flow cytometry density plots showing distribution of EdU (replicating cells) and DNA content (Propidium Iodide, PI) to determine the proportion of cells in G1/S (green) and S/G2M (purple). RS4;11 cells were pulse-labelled for 30 min with EdU, treated with DMSO or AZD1775, and chased for the times indicated. **g** Overview of the experimental setup for characterization of drug response dynamics to distinguish pre-existing and induced drug tolerance

*WEE1* expression was previously reported to be higher in primary ALL blasts compared to normal mononuclear cells [38]. To determine whether specific leukemia subtypes may rely more on *WEE1* expression, we used our data resource Hemap (http://hemap.uta.fi/) which contains curated genome-wide transcriptomic data from more than 30 hematologic malignancies [39]. Interestingly, *WEE1* expression was significantly higher (FDR < 0.01, Wilcoxon test) in the KMT2A-r ALL subtype (Fig. 1a, see also Additional file 1: Fig. S1a comparing KMT2A::AFF1, KMT2A::MLLT1 and KMT2A::MLLT3). Higher *WEE1* expression in KMT2A-r compared to other ALL subtypes raised the possibility that KMT2A-r cells may be more vulnerable to targeted inhibition of the WEE1 kinase. However, since WEE1 activity is regulated also at the protein level via phosphorylation, we performed further functional evaluation comparing different leukemic cell lines. First, to assess the sensitivity of leukemic cells that represent different ALL subtypes and lymphoid lineage differentiation states to WEE1 kinase inhibition, we treated a panel of 15 ALL cell lines with increasing concentrations of AZD1775 (Fig. 1b, refer to Additional file 1, Table S1 for GI50 data). Acute drug treatment induced apoptosis as measured by activation of caspase-3/7 and reduced cell viability in a dose-dependent manner in all cell lines (Fig. 1b, Additional file 1: Fig. S1b-c).

Next, we assessed whether the response to AZD1775 was sustained over time, by measuring recovery of proliferation post-treatment in leukemia cell lines, comparing the different chromosomal translocations, including *ETV6-PDGFRB* (Nalm-6), *TCF3-PBX1* (697, RCH-ACV and Kasumi-2), *ETV6-RUNX1* (REH), and KMT2Ar-ALLs (RS4;11, SEM, ALL-PO and KOPN8). Cells were treated with AZD1775 at sublethal dose (GI50 concentration) for 3 days, followed by drug washout and re-expansion in drug-free media. Although acute response to AZD1775 (GI50) did not associate with any specific ALL subtype (Additional file 1: Fig. S1b), WEE1 inhibition markedly attenuated re-proliferation following drug washout in KMT2A-r cells as opposed to non-KMT2A-r cells (*p* = 0.0094) (Fig. 1c, Additional file 1: Fig. S1d). Thus, the KMT2A-r subtype with the highest expression of *WEE1* exhibits significantly reduced recovery, in line with the patient genomics cohort result (Fig. 1a). To provide independent cell line evidence based on Crispr-Cas9 targeting across hematopoietic malignancies, we further analyzed *WEE1* gene dependency scores using the DepMap portal (https://depmap.org/portal/) and found a greater dependency on WEE1 in leukemia cell lines with higher *WEE1* expression (Spearman *r* = − 0.336, Additional file 1: Fig. S1e). However, characterization of the response to AZD1775 monotherapy cycles in vivo, using a primary KMT2A-r patient-derived xenograft (PDX) model (MLL-7, *KMT2A::AFF1*) [40] transplanted to NOD.Cg-*PrkdcscidIl2rgtm1Wjl*/SzJ (NSG) mice (*N* = 3 per group), did not show a strong survival benefit (Fig. 1d, AZD1775 median survival; 44 days, DMSO median survival; 42 days, see also Additional file 1: Fig. S1f-j), warranting a therapy strategy informed by cellular properties that contribute to drug tolerance or acquired resistance.

To explore the molecular determinants of the AZD1775 response, we selected KMT2A-r RS4;11 cells and non-KMT2A-r Nalm-6 cells for an in-depth analysis. First, we confirmed that AZD1775 reduced phosphorylation of CDK1 on tyrosine 15 [41], increased PARP cleavage and triggered DNA damage rapidly, as measured by phosphorylation of γH2AX (Fig. 1e, RS4;11 cells), according to the established mode of action of AZD1775. To confirm that RS4;11 cells treated with AZD1775 enter G2/M prematurely, cells were pulse-labelled with EdU and treated with DMSO or AZD1775 for different times, up to 32 h. As shown in Fig. 1f, while DMSO-treated cells divided and re-entered G1-phase, a large proportion of AZD1775-treated RS4;11 cells stalled at G2/M and failed to proceed through the cell cycle.

Analogous to KMT2A-r patients who initially respond to treatment but then relapse, the potent acute response of KMT2A-r cells in vitro was followed by recovery of proliferation after removal of AZD1775 (Fig. 1c). We reasoned that the cell characteristics associated with drug tolerance may originate through non-genetic heterogeneity from (1) a pre-existing subpopulation of drug-tolerant cells that are enriched during treatment, or alternatively these characteristics (2) are acquired during treatment through drug-induced cell state transition [21, 42, 43]. The first scenario would manifest as a gradual increase in cell phenotype/mutation frequency, limited by the cell doubling rate. The latter would involve shifts in gene expression programs induced by drug exposure, driven by rapid changes in TF activity, where only a fraction of the cells are expected to adapt to a new, possibly reversible cellular state that supports drug tolerance. Considering these two possibilities (Fig. 1g), we characterized the early shifts in gene expression programs using a combination of genomics assays. Concomitantly, we quantified the recovery capacity over extended time periods to assess the phenotype of drug-tolerant cells post-treatment.

### WEE1 kinase inhibition triggers a rapid transcriptional response and emergence of multiple cell states

**Fig. 2:**
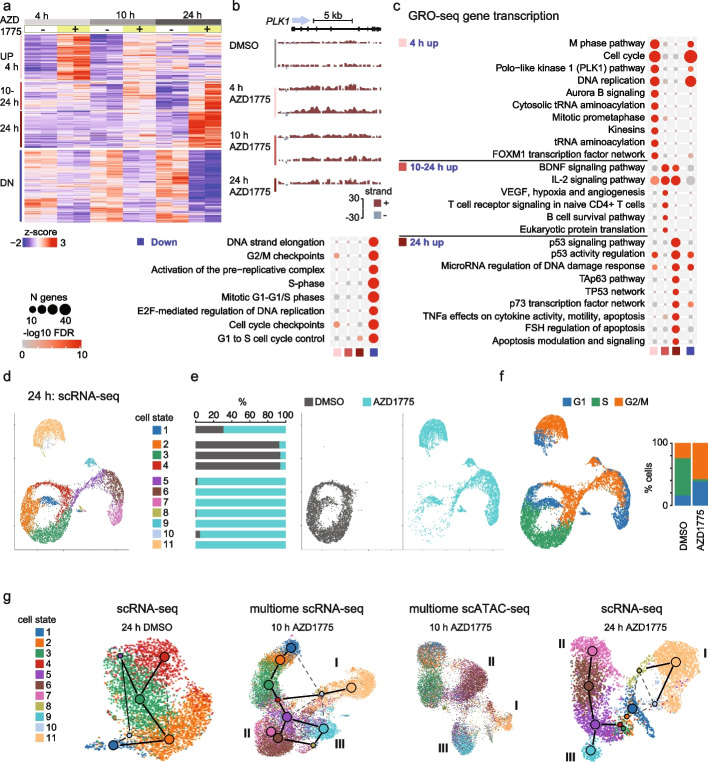
WEE1 kinase inhibition triggers a rapid transcriptional response and emergence of multiple cell states. **a** Heatmap illustrating the magnitude and direction of changes in the GRO-seq signal (*z*-score; tones of red indicate high level, tones of blue low level) for annotated gene transcripts. Gene clusters that correspond to regulation patterns 4 h up, 10–24 h up, 24 h up, and down are shown. **b** GRO-seq signal is illustrated at *PLK1* gene region from replicate samples collected at 4, 10, and 24 h (top left). Signal from + / − stand is plotted above (in red) or below (in gray), respectively. **c** Enrichment of biological processes for GRO-seq gene clusters (right: upregulated with distinct time profile, left: downregulated) shown as dot plots. Size of the dot corresponds to the number of genes from the gene set and − log10 FDR value is shown in color. **d–f** Low-dimensional UMAP projection and scRNA-seq transcriptome-based clustering for the RS4;11 cells from DMSO- and AZD1775-treated cells are shown. Colors correspond to cell state assignment (in **d**), treatment (in **e**), and assigned cell cycle status (**f**). Quantification of cell proportions across categories is shown in **e** and **f**. **g** UMAP based on transcriptome and chromatin access profiles generated separately for DMSO- and AZD1775-treated cells. Graph connectivity (PAGA) is visualized on scRNA-seq maps where connecting lines indicate putative cell state transition paths. Multiome data from 10 h includes scATAC-seq from the same cells. The colors correspond to assigned cell states based on 24 h scRNA-seq data (in **d**). Three treatment-specific cell fates that are reproducibly found in both time points and data modalities are indicated by roman numerals (cell fate I, II and III, see also Additional file 1: Fig. S2a)

We first performed global run-on sequencing (GRO-seq) to assess early effects on newly synthesized RNA at gene and enhancer regions at 4, 10, and 24 h (< population doubling time). The time course GRO-seq profiles identified 1239 significant gene transcription changes across time points (F-test FDR < 0.001). The temporal response to AZD1775 in bulk (Fig. 2a) could distinguish (1) genes primarily upregulated at 4 h, exemplified by the GRO-seq signal at the *PLK1* gene locus (Fig. 2b), (2) genes peaking in upregulation at 10 h, (3) genes most strongly upregulated at 24 h, and (4) genes downregulated at all time points (see also Additional file 2, Table S2). Pathway enrichment analysis revealed genes associated with regulation of the cell cycle (FDR 4^-17^), DNA replication (FDR 1^-12^), PLK1/AURORA signaling (FDR 1^-15^, 4^-12^), FOXM1 TF network (FDR 3^-6^), and M-phase pathways (FDR 2^-19^) in the early (4 h) upregulated cluster (Fig. 2c, Additional file 2, Table S2). Genes functionally related to cell cycle checkpoints (FDR 2^-20^), DNA-strand elongation (FDR 3^-19^), activation of pre-replication complexes (FDR 7^-18^), and S-phase (FDR 9^-18^) were repressed in the bulk genomics data (Fig. 2a bottom, “down”). B cell survival pathway was enriched (FDR 5^-2^) at 10 h, preceding upregulation of apoptosis modulation and signaling (FDR 2^-3^) related genes at 24 h.

Since WEE1 inhibition interferes with a central decision point of cell fate control during the cell cycle, we anticipated that this perturbation would result in cells occupying distinct states. To expose the diversification and nature of responses of individual cells, we therefore performed single-cell transcriptome and chromatin accessibility measurements. scRNA-sequencing revealed that 24 h after treatment with AZD1775, the surviving cells analyzed alongside DMSO-treated cells formed 11 cell clusters (Fig. 2d). We refer to these clusters as cell states hereafter. As determined by expression of cell cycle phase-specific markers, states 2, 3, and 4, correspond to different cell cycle phases present in DMSO-treated cells. State 1 corresponded to G0/G1 cells from both treatments, while clusters 5–11 were almost completely represented by AZD1775-treated cells (Fig. 2e). This initial analysis supports the scenario that AZD1775 treatment rapidly impacts the gene regulatory network, leading to the emergence of new cell states with unique active transcription profiles. As noted in the bulk GRO-seq analysis, treated and surviving cells had reduced number of cells in the transcriptional state associated with S phase and an enrichment of cells in G1 or G2/M phase (Fig. 2f), indicating an arrest at the G1/S checkpoint and a possible failure to progress through mitosis, consistent with the flow cytometry data of Fig. 1f.

Since the bulk GRO-seq analysis indicated ongoing transition and possible pro-survival adaptation of cells arising already at 10 h, we performed multiome single-cell RNA- and ATAC-seq to assay simultaneously from the same cell how open chromatin sites influence the transcriptional response and cell state diversity. We also compared the cell states to the scATAC-seq profile acquired at 24 h from parallel cell cultures.

First, to investigate the correspondence between cell states identified from the single cell transcriptome and clustering of cells defined by their chromatin accessibility, we examined the transcriptome and chromatin dynamics by separating the cells by treatment from 10 and 24 h and performing RNA velocity and PAGA graph analysis (Fig. 2g, transcriptome-based cell state labels from 24 h scRNA-seq are shown, see also Additional file 1: Fig. S2a-d and Methods). The predominant cell state dynamics (Fig. 2g, left) in DMSO-treated cells matched to active cell cycle transitions, as indicated by lines on the UMAP based on PAGA graph analysis (see Methods). By contrast, AZD1775-treated cells at 10 and 24 h (Fig. 2g, middle and right, respectively) followed a trajectory along three distinct branches. The first branch is dominated by cell state 11 (cell fate I), the second branch corresponds to a succession of cell states from 5 to 7 (cell fate II), and a third smaller population of cells branches into cell state 9 (cell fate III) (Fig. 2g). Each trajectory is unique to the AZD1775-treated cells and the distinct genome-wide changes are detectable as early as the 10 h time point, thus supporting a tolerance-inducing transcriptional response at the level of transcription and chromatin access.

Altogether, despite the highly heterogeneous response, we found a high concordance between the cell subpopulations analyzed using both RNA- and ATAC-seq analysis (Additional file 1: Fig. S2a), and between bulk and single-cell pathway analyses (Fig. 2c, Additional file 1: Fig. S2e, Additional file 3, Table S3).

### Forced mitotic entry in response to AZD1775 treatment arrests cells from S-phase in a condensed mitotic-like chromatin state

**Fig. 3:**
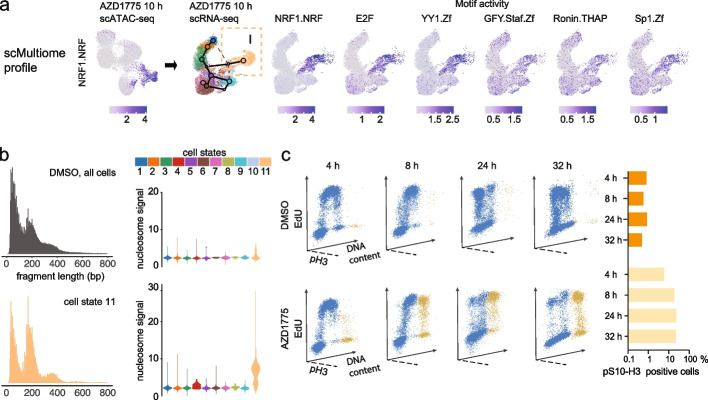
Forced mitotic entry upon AZD1775 treatment arrests cells from S-phase in mitotic-like chromatin state. **a** TF motif activity score (chromVAR) is shown for NRF1, E2F, YY1, GFY, Ronin, and Sp1 with highest score in cell fate I. To match with scRNA-seq cell states, TF motif activities are visualized on the 10 h scRNA-seq UMAP from the 10 h multiome profile. The NRF1 motif with highest cluster-specific activity (summarized in Additional file 4, Table S4) is shown on multiome scATAC-seq UMAP (10 h). Darker tones correspond to high motif activity score. **b** Typical scATAC DNA fragment length distribution in RS4;11 cells (24 h DMSO-treated cells, top left) compared to cells assigned to cell state 11 (bottom left). The nucleosome signal metric calculated from the ratio of fragments between 147 and 294 bp (mononucleosome) to fragments < 147 bp (nucleosome-free) across cell states is shown as violin plots (right panel, top: DMSO, bottom 10 h AZD1775-treated cells). **c** Three-dimensional (3D) flow cytometry dot plots showing the distribution of EdU (replicating cells, Y-axes) versus DNA content (X-axes) and pS10-H3 positive cells (Z-axes). RS4;11 cells were pulse-labelled for 30 min with EdU, treated with DMSO or AZD1775 and chased for the times indicated. Mitotic status was analyzed by labelling of phosphorylated S10-H3 (pH3) at the indicated time points. Graphs (right panel) represent the proportion of pS10-H3 positive cells in DMSO or AZD1775-treated cells

Next, to elucidate whether the transcriptome and chromatin dynamics indeed reflect diversification into functionally different cell fates, we analyzed the cell states in more detail. We found that state 11 distinguished a distinct subpopulation (cell fate I) that was prevalent (20%) already after 10 h and constituted 32% of cells at 24 h of AZD1775 treatment (Fig. 2g, middle and right, respectively). Based on scATAC-seq TF motif analysis, these cells had elevated S-phase (E2Fs, YY1, Additional file 4, Table S4) and mitotic checkpoint (NRF1, Ronin, Sp1, Additional file 4, Table S4) TF motif access (Fig. 3a and Additional file 1: Fig. S3a-b). Simultaneously, these cells showed evidence of mitotic entry downstream the forced activation of CDK1 based on the scATAC-seq fragment length distribution, with cells corresponding to cell fate I exhibiting a condensed chromatin state and elevated nucleosome signal metric (Fig. 3b, Additional file 1: Fig. S3c,d). Moreover, pathway enrichment from scRNA-seq cell cycle phase marker genes was consistent with a mitotic-like state (Additional file 1: Fig. S2e and Additional file 3, Table S3, FDR 1.23^-24^). Premature entry into mitosis can extend mitosis and enhance cell death [44]. In agreement with delayed mitotic exit, the fraction of phosphorylated serine 10 histone 3 (pS10-H3) with 4N DNA content increased with treatment time, reflecting a mitotic-like arrest (Fig. 3c).

We also performed scRNA-seq in non-KMT2A-r Nalm-6 cells and in sharp contrast to RS4;11, the majority of AZD1775-treated Nalm-6 cells retained a population in the active cell cycle (matched to cell states 2–4), in line with their ability to rapidly recover and proliferate upon drug withdrawal (Additional file 1: Fig. S3e). Accordingly, phosphorylation of S10-H3 was only transiently increased in Nalm-6 cells, while it was continuously high in RS4;11 cells treated with AZD1775 (Additional file 1: Fig. S3f), supporting a reduced capacity of KMT2A-r cells to cope with AZD1775-driven CDK-activation and replication stress compared to non-KMTA2-r cells.

### AZD1775 triggers unscheduled replication characterized by genome-wide p53 response

**Fig. 4:**
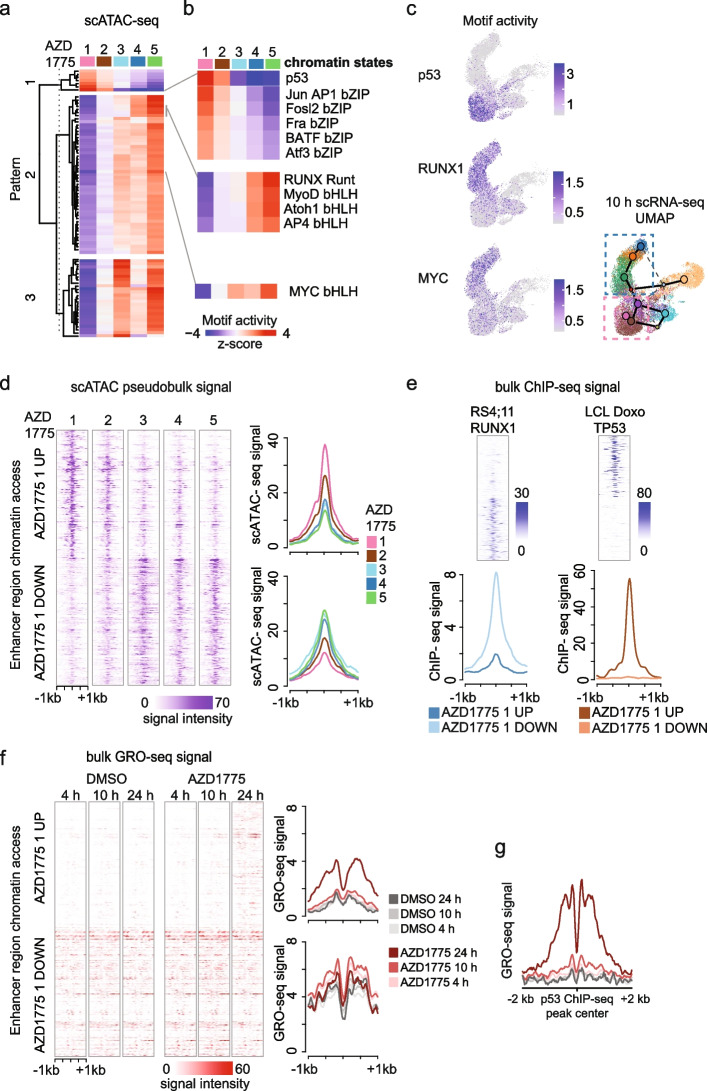
p53-driven gene regulatory response initiates from transcriptional silent chromatin and anti-correlates with RUNX1 binding. **a** TF motif activity score (scATAC, 24 h) across chromatin states 1–5 (with uniform QC metrics, refer to Additional file 1: Fig. S3d) in AZD1775-treated cells is shown as a heatmap, where TF motifs (in rows) are clustered into three main patterns. **b** Pattern 1 TF motifs with highest activity score in cell fate II (top panel). Selected pattern 2 TFs are shown for comparison (bottom panel). **c** p53, RUNX1, and MYC TF motif activity (10 h) visualized on UMAPs, as in Fig. 3a. **d** Pseudobulk scATAC-seq signal at − / + 1 kb at 200 up- and downregulated enhancer regions most specific to AZD1775 chromatin state 1 (AZD1775 1) vs other cell states. In the heatmap (left), each row corresponds to one enhancer and brighter color tones to higher access level. The magnitude and direction of changes in the cell state-specific chromatin accessibility signal across chromatin states 1–5 are summarized as average signal histograms (right). **e** ChIP-seq signal profile of RUNX1 in RS4;11 cells (DMSO) and p53 from doxorubicin (Doxo) stimulated lymphoblastoid cells (LCL). TF occupancy signal heatmap and histogram at the same enhancer regions as shown in **d**. **f** GRO-seq signal heatmap (left) and histogram (right) at the same enhancer regions as shown in **d**. Line color corresponds to treatment time and condition. **g** GRO-seq signal histogram at ChIP-seq-based high-occupancy p53 binding sites from LCL shown as in **f**

Comparison of the other cell clusters detected from scATAC-seq (referred to as “chromatin states”) revealed that AZD1775 induced genome-wide changes in chromatin accessibility with overall highly distinct TF motif activity patterns (Fig. 4a) that could underlie the emergence of distinct cell fates upon replication stress.

Elevated accessibility at the binding motifs for p53 (and bZIP-factors JUN/AP, FOS, Atf) was characteristic of AZD1775-specific chromatin states 1 and 2 (Fig. 4b, Additional file 4, Table S4). The TF motif activity score, visualized on the scRNA-seq map (Fig. 4c), reveals that these cells correspond to the branch leading to cell states 6–7 (26% 10 h, 32% 24 h, cell fate II) (Fig. 4c, see also comparison of RS4;11 and Nalm-6 in Additional file 1: Fig. S4a). Given that p53 modulates the senescence program in response to replication stress, we assessed the proportion of senescence following AZD1775 treatment. Indeed, we found a high gene set score for the senescence pathway in cell states 6–7 (Additional file 1: Fig. S4a), and an increased proportion of SA-β-Gal-positive cells in AZD1775-treated KMT2A-r compared to non-KMT2A-r cell lines (Additional file 1: Fig. S4b). This is in line with the increased mRNA expression of components related to the double-stranded DNA (dsDNA) sensor pathway (*cGAS*, *STING*) and factors of the senescence-associated secretory pathway (SASP, *HMGB2*) in RS4;11 cells (Additional file 1: Fig. S4c). Accordingly, AZD1775-treated Nalm-6 cells used for comparison exhibited a much smaller (20.4% respective) cell cluster matching p53-regulated gene sets (Additional file 1: Fig. S4a). Additionally, the activation of caspase 3/7 was higher in KMT2A-r cells compared to non-KMT2A-r cells (Additional file 1: Fig. S1c).

Based on the scATAC-seq profiles of the distinct cell states, the elevated p53 TF motif access (Fig. 4a, in pattern 1) anti-correlated with two other TF motif access patterns (Fig. 4a, patterns 2 and 3). Closer inspection of pattern 2 revealed mutual exclusivity with the lymphoid progenitor TF RUNX1 (clustered together with bHLH motifs recognized, e.g., by AP4) (Fig. 4b, Additional file 1: Fig. S4d). Notably, RUNX1 is a key TF regulating target genes downstream of KMT2A::AFF1 in a feedforward loop [25] and was expressed at higher levels in KMT2A-r patients compared to other ALL subtypes (Additional file 1: Fig. S4e).

To further explore the relationship between cell state-specific chromatin accessibility and transcriptional regulation, we visualized the scATAC-seq signal profile at enhancer regions with the highest and lowest chromatin accessibility in chromatin state 1 (Fig. 4d, AZD1775-treatment). Next, we generated RUNX1 ChIP-seq profile from RS4;11 cells (basal state) and retrieved p53 ChIP-seq signals from doxorubicin-stimulated lymphoblastoid cells (Fig. 4e). The average TF occupancy signals at these cell fate II-specific high chromatin access regions showed high p53 and low RUNX1 signal, while an opposite profile characterized the low accessible regions, providing further confirmation of their mutually exclusive TF activities. Notably, cells with high RUNX1 TF motif activity in AZD1775-treated cells (chromatin states 4 and 5) corresponded to transcriptome cell states 1–4 (also present in the basal state), and their relative proportion decreased from 35% at 10 h to 11% at 24 h.

Next, we quantified transcription from enhancers (enhancer RNA, eRNA) at these same regions (AZD1775, chromatin state 1). This regulatory region activity profile showed that enhancers in cell fate II-specific accessible chromatin were transcriptionally silent at 4 and 10 h (Fig. 4f). This is in agreement with gene-level GRO-seq results that indicated delayed upregulation of the p53-signaling network and pathways regulating apoptosis (cluster active at 24 h, Fig. 2a, Additional file 2, Table S2). Enrichment of p53 (*p*-value 1^-47^) and bZIP (*p*-value 1^-3^) binding motifs in enhancer regions active at 24 h was independently validated in the bulk GRO-seq data (see “Methods,” Fig. 4g, Additional file 1: Fig. S4f, Additional file 5, Table S5). Hence, inducing a p53 response from initially silent regulatory regions could allow cells time to adapt and initiate pro-survival responses.

### CDK-mediated degradation results in loss of RUNX1 in response to AZD1775

**Fig. 5:**
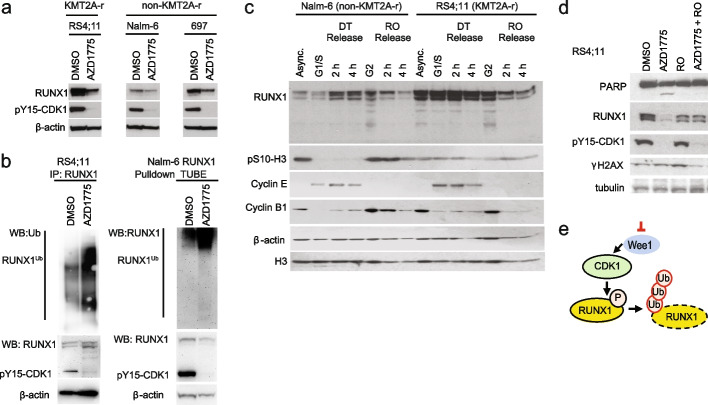
WEE1 inhibition promotes CDK-driven RUNX1 protein degradation. **a** RUNX1 protein levels in RS4;11, Nalm-6, and 697 treated with AZD1775 or DMSO for 24 h analyzed by immunoblotting. Phosphorylation CDK1 was detected with pY15-CDK1 antibodies. β-actin is a loading control. **b** Left panel: poly-ubiquitination of RUNX1 in RS4;11 cells treated with AZD1775 for 20 h (and with proteasome inhibitor MG-132 for the last 3 h). RUNX1 was immunoprecipitated (IP) under denaturing condition. RUNX1-ubiquitination was detected by immunoblotting with ubiquitin antibodies. Ten percent of whole cell lysate was used as input control for IP. Right panel: reciprocal pulldown of ubiquitin chains from whole cell lysates of Nalm-6 cells stably overexpressing RUNX1. Ubiquitin was pulled down using tandem ubiquitin binding entities (TUBEs), pulldown and input blotted and probed with RUNX antibody. Input for both experiments was probed with RUNX1, pY15-CDK1, and β-actin antibody. **c** RS4;11 and Nalm-6 cells were synchronized at different cell cycle stages (G1/S or G2) by double thymidine block (DT) or by treatment with RO3306 (RO) (CDK1 inhibitor), and released for the indicated time points. RUNX1 protein levels were analyzed by immunoblotting of whole cell lysates using RUNX1 antibodies. Asynchronous (Async) cells were treated with DMSO. Expression of cyclin E, cyclin B1, and phosphorylation of pS10-H3 was analyzed as indicated. β-actin and H3 are loading controls. **d** RS4;11 cells treated with AZD1775, RO3306 (RO), or their combination (AZD1775 + RO) for 24 h. Expression of RUNX1, cleaved PARP1, phosphorylation of Y15-CDK1, and phosphorylation of S139-γH2AX was analyzed by immunoblotting of whole cell lysates with the antibodies as indicated. Tubulin is a loading control. **e** Schematic illustration of mechanism linking CDK1 activity and RUNX1 protein degradation following WEE1 inhibition by AZD1775

To further characterize the decline in RUNX1 activity and other pattern 2 TFs, including the KMT2A::AFF1 TF target MYC that promotes leukemia survival [25], we performed immunoblot analysis (in bulk) and found that AZD1775 treatment decreased RUNX1 protein expression levels in KMT2A-r RS4;11 and SEM cells, and in several non-KMT2A-r ALL cell lines (Fig. 5a, Additional file 1: Fig. S5a). The hematopoietic stem cell TFs GATA2 and MYC similarly had declining protein expression following AZD1775 treatment (Additional file 1: Fig. S5b). In comparison, ATF4 that acts as transcriptional activator of the integrated stress response was transiently induced at 4 h (Additional file 1: Fig. S5b).

RUNX1 was previously reported to be degraded in a CDK-dependent manner by the APC ubiquitin ligase complex during mitosis [45, 46]. According to the scATAC-seq TF motif analysis in DMSO-treated cells, the RUNX1 and bHLH motifs exhibit the highest accessibility in DMSO chromatin cluster 1 (matched to G1 phase, or early S-phase in the multiome profile, Sankey plot Additional file 1: Fig. S2a) and lowest accessibility in G2 phase (Additional file 1: Fig. S5c). We hypothesized that in the absence of inhibitory phosphorylation by WEE1 (due to AZD1775 treatment, Fig. 5a, pY15-CDK1), the hyperactivation of CDK1/2 may promote ubiquitination and proteasomal degradation of RUNX1 during G2/M phase. Supporting this, AZD1775 treatment did not alter *RUNX1* mRNA expression levels in RS4;11 cells or in other B-ALL cell lines (Additional file 1: Fig. S5d), but increased RUNX1 protein ubiquitination upon proteasomal inhibition (Fig. 5b). We next verified that RUNX1 levels decreased following release from a G2/M-phase block as compared to cells released from a G1/S-phase block (Fig. 5c). Concurrent treatment with the CDK1 inhibitor RO3306 and AZD1775 partially rescued downregulation of RUNX1 protein, and reduced PARP cleavage and γH2AX (Fig. 5d), further supporting CDK1-driven ubiquitination and proteasomal degradation of RUNX1 in response to WEE1 inhibition (illustrated in Fig. 5e).

Together, these results suggest that WEE1 inhibition may interrupt a core KMT2A-r transcriptional regulatory program involving RUNX1-MYC-GATA TFs through reduced protein expression.

### Stress- and pre-B-state regulatory programs distinguish a drug-tolerant sub-population with pre-BCR and BCL6 gene loci activation

**Fig. 6:**
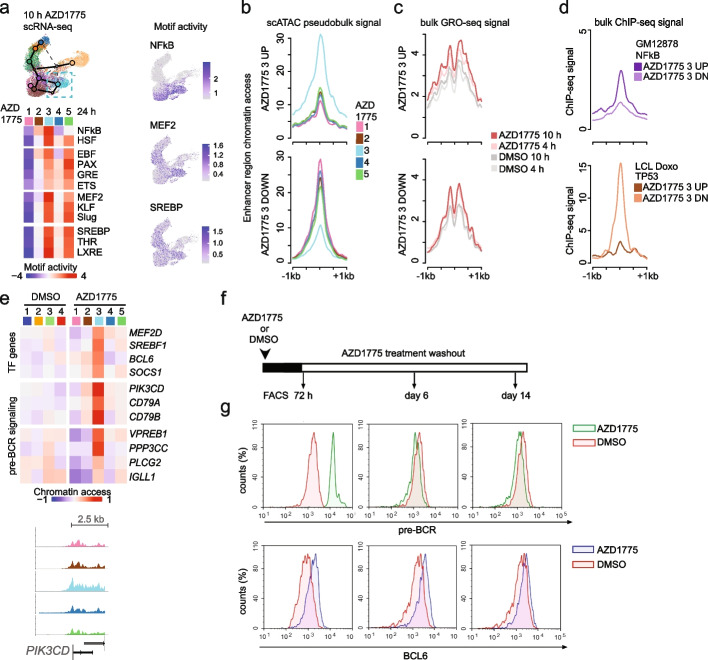
Stress- and pre-B-state regulatory programs distinguish a drug-tolerant sub-population with pre-BCR and BCL6 activation. **a** TF motif activity scores across AZD1775 chromatin states (1–5) is shown for TFs with high score in cell fate III (Pattern 3, see Fig. 4a) (left). NFkB, MEF2, and SREBP motif activity scores are visualized on the 10 h scRNA-seq UMAP (right). **b** scATAC-seq signal at − / + 1 kb at 200 up- and downregulated enhancer regions most specific to AZD1775 chromatin state 3 (AZD1775 3) vs other cell states is shown as average signal histogram, as in Fig. 4d. **c** GRO-seq signal histogram at the same enhancer regions as shown in **b**. **d** ChIP-seq signal profile of NfKB in lymphoblastoid cells (LCL) and p53 from doxorubicin (Doxo) stimulated lymphoblastoid cells. TF occupancy signal histogram is shown at the same enhancer regions as shown in **b**. **e** Chromatin access at TF and pre-BCR signaling-related gene loci (gene body flanked by 2.5 kb up- and downstream) compared across AZD1775 chromatin states is shown as a heatmap (top). Aggregated scATAC-signal across cells assigned to each cluster is exemplified at *PIK3CD* gene region (bottom). The track colors correspond to 24 h AZD1775 chromatin state annotation. **f** Experimental setup for analysis of drug response and recovery dynamics. **g** Flow cytometry histograms of pre-BCR (upper panels) and BCL6 (lower panels) in RS4;11 cells treated with DMSO and AZD1775 for 72 h and following 6 and 14 days drug washout

Functionally, RUNX1 is part of a core regulatory circuit that acts as a switch, governing cell fate commitment [25]. Consequently, perturbing RUNX1 expression and TF activity dynamics by WEE1 inhibition may facilitate a transition to an alternative cell state. In line with this, the cell fate III subpopulation (cell state 9, Fig. 2g) had decreased RUNX1 motif activity (Fig. 4a, AZD chromatin state 3), but significantly high chromatin access of pre-B cell state TF motifs, including PAX5, EBF, GR, and MEF2C (FDR 2.8^-35^, 8.6^-41^, 1.5^-29^, 1.4^-99^, respectively), and high motif activity of TFs regulating cellular metabolism (SREBF, THR, LXR) (Fig. 6a, see also Additional file 1: Fig. S6a-b, Additional file 4, Table S4). As a distinct feature, cell state 9 (cell fate III)-specific open chromatin regions had highest NFkB (FDR 2.4^-69^) and heat shock factor (HSF, FDR 3.9^-67^) motif activity (Fig. 6a). Supporting activation of a functional TF circuitry, cell fate III cells had high expression of the respective TF target genes of SREBF (*LDLR*, *HMGCS1*), HSF (*HSPA1B*), GR (*TSC22D3*), NFkB (target gene set activity scores shown), and pre-BCR signaling (*BCL6*, *SOCS1*) (Additional file 1: Fig. S6c-e, Additional file 3, Table S3). The combination of stress- and pre-B fate-specific TF activation signifies a cell state transition in response to AZD1775 treatment that may confer a more stress-resistant phenotype. In a similar fashion as examining cell fate II-specific chromatin (Fig. 4d–f), we quantified enhancer regions that represent highest vs lowest sc-ATAC-seq signal in cell fate III (AZ chromatin cluster 3) and compared chromatin accessibility (Fig. 6b, scATAC-seq signal) and enhancer activity (Fig. 6c, GRO-seq signal). Interestingly, the regions with high access in cell fate III were transcribed, albeit at a low level, even in the basal state (gray lines, Fig. 6c), suggesting a primed state. Following AZD1775 treatment, these enhancers were quickly further activated (4 and 10 h profiles in red, Fig. 6c). Comparison to NFkB ChIP-seq signal in lymphoblastoid cells provided further confirmation that the activated chromatin regions in cell fate III-specific open chromatin harbor NFkB binding sites (Fig. 6d, upper panel). In comparison, the ChIP-seq signal at enhancers with lowest chromatin access revealed that these sites were p53-bound in lymphoblastoid cells treated with doxorubicin (Fig. 6d, lower panel), which could indicate active suppression of p53 chromatin recruitment in cell fate III [47]. The difference in the temporal dynamics of transcriptional activation of cell state II vs cell state III enhancers observed in RS4;11 cells, coupled with the reduced chromatin accessibility at p53 binding sites, may therefore contribute to the increased drug tolerance in cell fate III. Furthermore, chromatin accessibility increased at gene loci encoding *MEF2D*, *SREBF1*, *BCL6*, *SOCS1*, and components of the pre-BCR signaling pathway, all of which play central roles in the cellular survival pathways of B-lymphoid cells (Fig. 6e). Interestingly, this pattern resembles a previously recognized leukemia-associated TF circuitry that is active in the MEF2D-fusion subtype of ALL [48]. In line with the emergence of a drug-tolerant cell state through transcriptional regulation, the B cell survival pathway was significantly enriched (FDR < 0.05) in the activated gene cluster identified from bulk GRO-seq profile at 10–24 h (Fig. 2c). These data are consistent with leukemic cells adopting a BCL6 + pre-BCR + cell state having higher tolerance to AZD1775, concordant with results obtained in pre-BCR + Nalm-6 cells (Additional file 1: Fig. S4a-b).

To investigate whether the activation of cell fate III transcriptional program constitutes a potential escape mechanism that may support recovery following AZD1775 treatment, we analyzed pre-BCR and BCL6 protein expression in subsequent time points from cells treated with AZD1775 for 72 h, and after 6 and 14 days of recovery. Notably, the drug tolerant cells that persisted at 72 h matched the pre-BCR + /BCL6 + population (cell fate III) based on protein levels that were strongly upregulated in RS4;11 cells (Fig. 6f, g). This activation of pre-BCR and BCL6 in response to AZD1775 was reversible, albeit with a delay: pre-BCR expression returned to baseline levels by day 6 and BCL6 by day 14 after the removal of the drug (Fig. 6f,g, Additional file 1: Fig. S7a-b). Accordingly, RUNX1 could be detected at day 14 in cells that had recovered from treatment (Additional file 1: Fig. S7c). Consistent with this reversibility, the cells that recovered post-treatment (drug schedule shown in Additional file 1: Fig. S7a) retained their sensitivity to AZD1775 (Additional file 1: Fig. S7b, right), mirroring the response of the parental, untreated cells (Additional file 1: Fig. S7b, left). Additionally, both RS4;11 (Additional file 1: Fig. S7c-f) and Nalm-6 (Additional file 1: Fig. S7g-j) cells recovering from AZD1775 remained sensitive to other conventional drugs used in ALL treatment protocols (L-asparaginase, cytarabine, doxorubicin). Although our findings do not definitively rule out the alternative possibility (Scenario 1, Fig. 1g) that a pre-existing resistant cell subpopulation is selected for and expands during the recovery phase, they are more consistent with reversible cell state switching induced by WEE1 inhibition. Furthermore, treatment with L-asparaginase, cytarabine, or doxorubicin did not activate pre-BCR in KMT2A-r cells (Additional file 1: Fig. S7d-f), highlighting the specificity of the AZD1775-induced phenotype shift.

To investigate markers associated with cell state III in vivo, MLL-7-engrafted NSG mice were treated with AZD1775 or control vehicle (as detailed in Additional file 1: Fig. S8a). hCD45 + hCD19 + cells were isolated and subjected to scRNA-seq after 28 h and at day 6 post-treatment to identify early transcriptional changes. Gene enrichment analysis revealed significant over-representation of genes from the BCR signaling pathway in cluster 6 (Additional file 3, Table S3) of AZD1775-treated MLL-7 cells (28 h treatment and matched cells from day 6; FDR 1.03^-8^ and 1.15^-8^, respectively, Additional file 1: Fig. S8b). Resembling the profile found in RS4;11 cell state 9, expression of pre-BCR or BCR signaling, pre-B TF genes, and NFkB targets was increased, while *MYC* levels declined (Additional file 1: Fig. S8c-e). The expression pattern in vehicle-treated cells was more dispersed with only weak expression of *MS4A1* (encoding the differentiation marker CD20), the most significant marker gene for cluster 6 (Additional file 1: Fig. S8d). For comparative analysis, we also included an in vivo treatment profile from MEF2D-fusion ALL case (Additional file 1: Fig. S9). Positive correlation of TF expression rate could be detected at a cluster level for *MEF2D*-*BCL6* (Pearson *r* 0.8, *p*-value 0.056) and *BCL6*-*SREBF1* (Pearson *r* 0.9, *p*-value 0.016). Together, these data indicate that the cell phenotype with pre-BCR and pre-B fate TF expression is present in leukemia patient-derived cells.

### Sequential drug treatment can target non-genetic evolution of leukemic cells

**Fig. 7:**
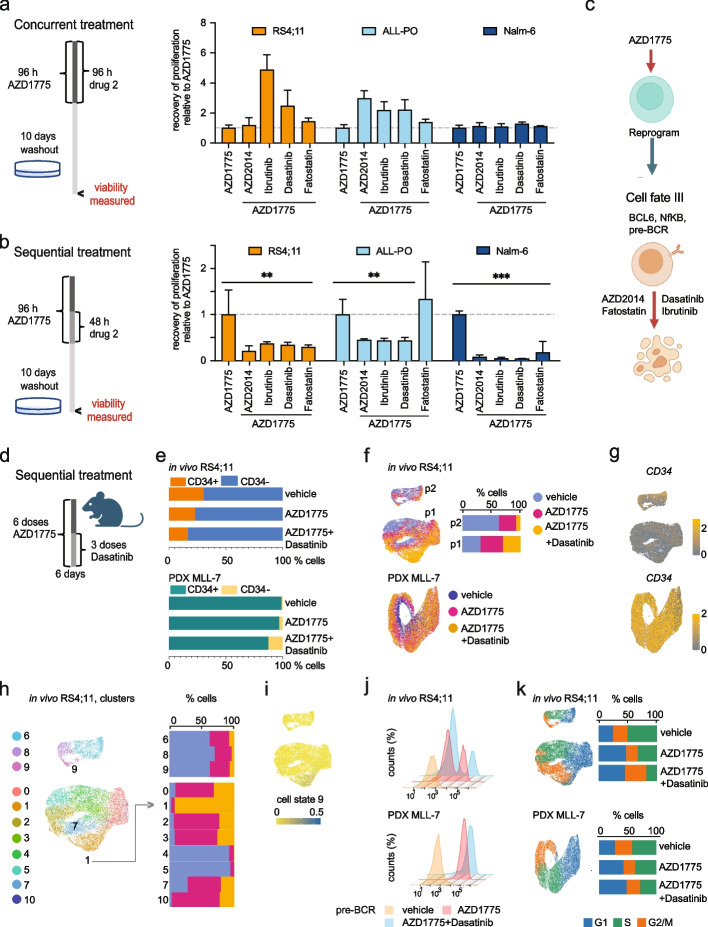
Targeting WEE1 and the drug tolerant cell state sequentially attenuates recovery of leukemic cells. **a,b** Left panels: Overview of the experimental setup. Cells were treated as indicated and allowed to recover in drug-free media for an additional 10 days. Right panels: **a** recovery of cell proliferation after concurrent treatment, following removal of the drugs. Cells were treated with 0.5 μM of AZD1775 and indicated drugs (1 μM) for 96 h. **b** recovery of cell proliferation after sequential treatment. Drug response relative to recovery of proliferation of cells treated with AZD1775 is shown. Regrowth was assessed using Alamar Blue stainings. ** denotes *p* < 0.01 and *** denotes *p* < 0.001 between AZD1775 alone and indicated drug combinations determined using Student’s *t*-test. Data are presented as mean ± SD. **c** Schematic model of the treatment strategy. **d** Schematic illustration of sequential treatment in vivo. Drugs were administered daily as outlined. **e** Proportion plot of immunophenotypic profiling of CD34 subpopulations in RS4;11 and MLL-7 xenograft models. **f** Proportion plots and UMAP visualization of treatment group in in vivo RS4;11 model (top). Treatment group data is shown for two distinct cell populations (p1 and p2, indicated on the UMAP). UMAP visualization of treatment group in MLL-7 cells across sample (below). **g ***CD34* mRNA level shown on UMAP RS4;11 in vivo model (top) and MLL-7 (below). **h** Cluster assignment in RS4;11 in vivo model shown on UMAP (left). Barplots of corresponding cell proportions per treatment group (right). Clusters corresponding to G/2 M phase are indicated on the UMAP. Cluster 1, with majority of cells corresponding to the drug combination group is highlighted. **i** Label transfer score for cell state 9 shown on UMAP (RS4;11 in vivo model). **j** Flow cytometric analysis of pre-BCR expression under treatment in RS4;11 and MLL-7 xenograft models. **k** Proportion plots and UMAP visualization of treatment group and cell cycle phase data in RS4;11 (top) and MLL-7 (below) xenograft models

Drugs disrupting the regulatory network activated in cell fate III could have high efficacy in preventing recovery of the remaining leukemic cell population. Therefore, we selected drugs that target pre-BCR signaling (dasatinib and ibrutinib) [49, 50]. Based on higher TF motif accessibility observed in cell state III for master regulators of cellular lipid metabolism, such as SREBF1, we also included inhibitors of metabolism (mTOR inhibitor AZD2014) and fatty acid synthesis (fatostatin) [51, 52]. To assess the potential of repositioning these drugs as effective combination therapies with AZD1775, we initially measured efficacy of each drug as single agents in the drug recovery setting (illustrated in Fig. 7a,b, Additional file 1: Fig. S10a). When used as monotherapies, these drugs did not affect recovery of proliferation (Additional file 1: Fig. S10a), and neither did they when administered concurrently in combination with AZD1775 (Fig. 7a). However, the sequential administration of AZD1775 in conjunction with several of these drugs effectively inhibited the recovery of RS4;11 and ALL-PO cells in vitro (Fig. 7b). This supports the strategy that targeting drug-induced adaptive survival pathways attenuated drug tolerance in KMT2A-r cells (Fig. 7c). Notably, administration of AZD1775 followed by AZD2014, dasatinib, or ibrutinib also strongly attenuated recovery of non-KMT2A-r Nalm-6 cells (Fig. 7b).

To corroborate these findings, we sorted and profiled CD45 + / CD19 + RS4;11 and MLL-7 cells following a sequential combination therapy (two doses of AZD1775 followed by daily doses with dasatinib combination started at 72 h) in xenograft mouse models (Fig. 7d, Additional file 1: Fig. S10b). The combination therapy resulted in a lower number of leukemia cells in both blood and spleen tissues (see Additional file 1: Fig. S10b), indicating that the treatment reduces the proliferation of leukemia cells compared to the control vehicle. To characterize the effect of treatment on cell phenotypes, we first performed immunophenotype analysis by flow cytometry using the stemness marker CD34. In MLL-7, nearly all cells (99%) treated with the vehicle were CD34 + , while AZD1775 treatment, and even more so the combination treatment, decreased the number of CD34 + cells (to 97 and 87%, respectively) (Fig. 7e, upper panels). In comparison, the RS4;11 cells exhibited higher immunophenotype diversity, with 30% CD34 + cells in vehicle, declining to 23% (AZD1775) and 16% (AZD1775 + dasatinib) (Fig. 7e, lower panel).

To examine the gene expression phenotypes, we subjected the cells to scRNA-seq. RS4;11 cells grouped across the treatments into two separate, actively dividing, cell populations, while a more uniform cell population was present in MLL-7 (UMAPs, Fig. 7f). Comparison of progenitor marker genes revealed higher *CD34* levels in the smaller population of RS4;11 (p2, Fig. 7f,g), referred to as stem-like from here on. In agreement with the immunophenotype analysis, cells obtained from vehicle-treated mice (62.4%) were more prevalent in the stem-like population, compared to cells from mice treated with AZD1775 alone (30.1%), or the combination with Dasatinib (7.5%) (Fig. 7f). In MLL-7, the scRNA-seq also identified cells with decreased *CD34* expression (Fig. 7g).

In the RS4;11 cells with low *CD34* expression (p1, Fig. 7f), cluster 1 showed the greatest proportion of cells treated with the combination, as indicated in Fig. 7h. Label transfer scoring for cell fate III was highest in this cluster that revealed a significant enrichment of pathways that control cell death (apoptosis, FDR 0.003, Additional file 3, Table S3) and upregulation of *TP53*, *GADD45A*, and *BAX* (Additional file 1: Fig. S10c), with concomitant decrease in cellular mRNA levels (Additional file 1: Fig. S10d,e, also visible in MLL-7 Additional file 1: Fig. S10f). These data are consistent with early apoptosis [53, 54] and imply that the combination treatment specifically affects and may trigger cell death in cells that resemble cell state III.

Subsequent analysis of cell state III markers, specifically pre-BCR and BCL6, revealed that AZD1775 increased protein levels of these markers in the two in vivo models (Fig. 7i, Additional file 1: Fig. S10g), confirming the treatment effects observed in vitro (Fig. 6g). When combined with Dasatinib, there was a notable decrease in pre-BCR expression in RS4:11 cells (Fig. 7j). In MLL-7, the combination resulted in a distinct population of pre-BCR-negative cells compared to treatment with AZD1775 alone (Fig. 7j).

Finally, scRNA-seq and DAPI staining indicated changes in the proportion of actively cycling cells following the combination treatment (Fig. 7k, Additional file 1: Fig. S10h-i). The combination treatment consistently decreased the population of cells in the S-phase in both xenograft models, with concomitant increase in G1-phase in MLL-7 and expansion of G2/M cells in RS4;11. Notably, cells that exhibited an early apoptotic gene expression signature in RS4;11 corresponded to G2/M or G2/M-to-G1 transitioning cells (depleted by treatment in MLL-7, as denoted by arrows on the UMAP plots separated by treatment, Additional file 1: Fig. S10h). This suggests that the observed effects may stem from cell death during the S and G2/M phases, arrest in the G1 or mitotic phase, or a combination thereof. Concordantly, subsequent Annexin V/PI staining on CD45 + /CD19 + RS4;11 and MLL-7 cells revealed increased cell death in both xenograft models post-treatment (Additional file 1: Fig. S10j).

Taken together, these results show that perturbing cell cycle regulation through inhibition of WEE1 kinase in KMT2A-r cells triggers multiple distinct genome-wide responses that include rewiring of TF programs, resulting in cell state diversification. The sequential administration of AZD1775 together with drugs targeting metabolism and pre-BCR signaling can disrupt a non-genetic evolution toward a drug tolerant state, a discovery that potentially offers new opportunities for drug repositioning to prevent or delay the regrowth of leukemic cells.

## Discussion

Targeted therapy holds promise in cancer treatment to reduce the toxicity of long-term chemotherapy. However, cancer cells can develop resistance to targeted treatments not only through the selection of genetic mutations but also through non-genetic heterogeneity, namely drug-tolerant states with phenotypic adaptations that allow cells to evade drug-induced lethality. Herein, we characterized in detail the genome-wide response and cell state evolution upon cell cycle checkpoint-targeted therapy through inhibition of the WEE1 kinase in ALL. Data-driven analysis from the HEMAP and the DepMap genomics resources combined with drug response profiling in different ALL subtypes revealed higher expression and increased WEE1 gene dependency in KMT2A-r ALL as compared to other genetic subtypes. We show that WEE1 inhibition and CDK1 activation led to degradation of RUNX1 and repression of the KMT2A-r stemness phenotype, with concomitant repression of CD34, MYC, and GATA2. Thus, contrary to currently held views that stemness features critically underlie drug resistance in KMT2A-r leukemias, we found that upon targeting their cell cycle control vulnerability, the drug-tolerant cells corresponded to a more differentiated phenotype with expression of pre-BCR and BCL6. We show that WEE1 inhibition followed by drugs targeting the drug-tolerant cell state regulatory program prevents recovery in KMT2A-r and pre-BCR + ALL cells. This synergy was observed only in the sequential, not in concurrent treatment schedule.

Upon WEE1 inhibition, most of ALL cell lines, including KMT2A-r cells, rapidly enriched for cell cycle markers consistent with deregulated DNA replication and unscheduled transition into G2M-phase, leading to cell death. However, the cell lines showed capacity to resume proliferation post-AZD1775 treatment within 1–14 days after removal of AZD1775. Using KMT2A-r RS4;11 cells as a model, we show that AZD1775 treatment results in early diversification in cell states, as evidenced by significant alterations at both transcriptional and epigenetic levels. Specifically, the single-cell profiles mapped the majority of cell states with extended mitotic arrest distinguished by condensed chromatin status (cell fate I) and strong p53 activation (cell fate II), consistent with induction of apoptosis and senescence pathways. In comparison, cell fate III had higher chromatin access at BCL6 and pre-BCR gene loci and TF motifs of NfKB, MEF2D, and the metabolic regulators SREBF and LXR. A reversible shift to pre-BCR + /BCL6 + state was confirmed at protein level (day 3) that may be attributable to drug-induced steady state (attractor) switch [21], consistent with a non-genetic and drug-induced acquisition of a drug tolerant cell phenotype through altered TF activity that persists several weeks in vitro. The comparison of enhancer activation signals in the cell fate-specific open chromatin at p53, RUNX1, and NFkB bound sites provided insight into how the alternative cell fates could arise downstream TF activity changes: a progressive response activating p53-bound sites in cell fate II diverted cells with unscheduled S-phase entry away from cell cycle and towards cell death and senescence through p53 activation at initially transcriptionally silent regions. In contrast, NfKB and BCL6 both correspond to TFs known to counteract p53-driven apoptosis and repress genes involved in sensing or responding to DNA damage [55–58]. The loss of chromatin access at RUNX1 occupied sites upon WEE1 inhibition, and concomitant loss of stemness markers, induction of differentiation-related TFs, activation of cell fate III-specific chromatin co-localizing with NFkB sites, and pre-BCR expression represent, to our knowledge, a previously unrecognized connection between cell cycle and cell fate regulatory circuits.

RUNX1, GATA2, and MYC are crucial drivers of leukemogenesis that belong to the core KMT2A-r gene regulatory network and represent regulatory targets of KMT2A-r fusions [25, 33, 59, 60]. Consequently, disruption of the RUNX1-GATA2-MYC progenitor program could potentially reroute cell fate decisions favoring survival in absence of RUNX1-MYC through the activation of alternative TFs. In particular KMT2A-r cells display strong addiction to RUNX1 for survival [33, 36]. The loss of RUNX1 through ubiquitin-mediated protein degradation reported here may impinge upon multiple surveillance mechanisms that when disrupted enhance the sensitivity of KMT2A-r cells to AZD1775. Alternatively, perturbations in the epigenetic control that are a hallmark of KMT2A-r leukemias, such as the increased transcriptional variability reported upon histone acetyltransferase KAT2A loss in KTM2A-r acute myeloid leukemia [37], may facilitate stochastic cell diversification upon stress or injury. Here, we examined the shift to pre-BCR + state, comparing drug-induced stress upon WEE1 inhibition or conventional chemotherapy drugs, and contrary to this more general mechanism found that the cell state diversification occurred specifically upon AZD1775 treatment. In addition, since RUNX proteins have been reported to function directly as integral regulators of DNA repair [35], and previous studies demonstrated that KMT2A-r cells have compromised the ATR-mediated S/G2-phase checkpoint [32], the inhibition of WEE1 in KMT2A-r cells may not only override the G2/M-phase checkpoint but also cause disruption of the G1/S checkpoint [1], resulting in CDK1/2-driven unscheduled origin firing, further enhancing p53 activation and senescence/apoptosis. These findings are thus consistent with the increased sensitivity of KMT2A-r cells to AZD1775 and the dependency on RUNX1.

Although KMT2A-r leukemias resemble the early lymphoid/multipotent progenitors by immunophenotype, recent single-cell characterization of patient cells has revealed that the transcriptional programs of a subset of cells present in primary bone marrow tissue match more differentiated pre-B-like states [24, 29]. Similarly, our previous single-cell genomics study deciphering features of drug resistance directly in ETV6-RUNX1 ALL patients during induction chemotherapy [23] demonstrated that although the diagnostic blasts matched a pro-B-like cell state, the persisting leukemic cells at day 15 are re-programmed towards a pre-B TF activity state. The new profiles acquired here from KTM2A-r drug response both in vitro and in vivo indicated increased chromatin accessibility of genes encoding pro-survival BCR − and PI3K signaling components and the co-expression of BCL6 and pre-BCR signaling, consistent with non-genetic adaptation contributing to treatment resistance. Recent research, including flow-sorted subpopulations and cellular barcoding studies [61–63], has shed light on the inherent capacity of leukemia blasts, across different stages of immunophenotypic maturation, to propagate the disease. This emphasizes the clinical importance of considering non-genetic pathways to drug tolerance in treatment strategies.

BCL6 and pre-BCR signaling can form an oncogenic feedback loop in ALL cells that promotes survival signaling via SRC family kinases, SYK, ZAP70, and downstream PI3K activation [64]. Due to the recent development of efficient drugs inhibiting downstream pathways (dasatinib, ibrutinib), this elevated drug tolerance could potentially be disrupted. Our results further implicate regulation of lipid metabolism (LXR, SREBF) as a concomitant change that has been recognized in immune cell activation and oncogenic PI3K signaling in different cancers [65, 66]. By interfering with pro-survival signaling through targeting lipid metabolism or inhibition of BCR signaling, sequential administration of mTOR inhibitor AZD2014, fatostatin, and inhibitors of BCR-signaling (dasatinib, ibrutinib) strongly suppressed recovery of KMT2A-r cells in vitro. Notably, this strategy also potently blocked recovery in non-KMT2A-r Nalm-6 cells, indicating that sequential targeting of WEE1 along with pre-BCR or metabolic activity may be broadly effective in ALLs.

Our results, consistent with a number of ALL drug therapy studies, indicate that targeting specific cancer vulnerabilities using carefully designed drug scheduling may represent the most useful measures for improving leukemia treatment success and counteracting disease recurrence. Despite existing data of potent chemo-sensitizing activities of AZD1775 in combination with different cytotoxic agents [67–70], WEE1 inhibitors have not been tested, to our knowledge, in ALL clinical trials. To date, several ongoing efforts aim to introduce immunotherapy in B-ALL treatment which requires implementation of a successful chemotherapy regime to initially reduce the leukemia burden, followed by immunotherapy. Combination therapies with WEE1 inhibitors may be attractive in this setting, reducing leukemia burden by inducing cell death through the cell-intrinsic mechanism, as characterized here. In solid cancers, favorable responses have been reported in phase 3 trials, though concerns in toxicity remain when combined with strongly cytotoxic backbone drug therapies. Our results show that several low toxicity drugs, already in clinical use for ALL or other non-communicable disease, potentiate the efficacy of WEE1 inhibitors in leukemia to overcome this challenge. In the preclinical xenograft models that represent the more diverse in vivo microenvironment, we could recapitulate the decrease in stemness markers, expression of pre-BCR and BCL6 and elevated cell death, as an indication of treatment efficacy following a sequential scheme. In follow-up, optimizing the drug scheduling to the in vivo cell state dynamics and assessing the long-term efficacy of the drug combination across a broader panel of KMT2A-r leukemias should be conducted. Furthermore, AZD1775 was recently reported to additionally activate immune signaling through activation of STING and STAT1 pathways, or the double-stranded RNA viral defense pathway, and sensitize solid tumors to immune checkpoint therapy [71, 72]. Pre-clinical testing in xenograft models lacking the immune system, as used here, may therefore only partially represent the efficacy of AZD1775 therapy.

## Conclusions

In summary, the combination of biochemical assays and cellular resolution genomics analyses provided new insight on the remodelling of the gene regulatory landscape upon cell cycle checkpoint targeting. Based on the results, we propose a strategy for WEE1 inhibition in ALL that anticipates the TF network re-wiring and the transition to a pre-BCR + BCL6 + cell state. This strategy targets the drug-tolerant phenotype through novel sequential drug administration, establishing a proof-of-concept that could be utilized in the design of new targeted drug screens and clinical trials.

## Methods

### Cell culture

A panel of ALL cell lines consisting of T-ALL: T-ALL1, Peer, Molt-16, and B-ALL cell lines: Nalm-6 (ETV6-PDGFRB); 697, RCH-ACV, Kasumi-2, MHH-Call3 (TCF3-PBX1); REH (ETV6-RUNX1); RS4;11, SEM, ALL-PO, KOPN8 (MLLr-ALL); SupB15, TMD5 (BCR-ABL) were purchased from the Department of Human and Animal Cell Lines (DSMZ). Cell lines were maintained in RPMI1640 (Gibco, Thermo Fisher) supplemented with 10% FBS (Gibco, Thermo Fisher) and 2 mM L-glutamine (Gibco, Thermo Fisher) and 10 mM HEPES (Gibco, Thermo Fisher). To generate stable Nalm-6-RUNX1 cell lines, *RUNX1* cDNA (NM_001754, originally from Origene Technologies Inc, MD, USA, cat #RG223809) was cloned into pLVX-Tight-Puro vector (Clontech) using NotI and EcoRI restriction sites. Nalm-6 cells were transduced with the regulatory vector TetOn and subsequently with pLVX response vectors (either without an insert or with RUNX1).

### Drug and dose response assessment

AZD1775, AZD2014 (Astra Zeneca), Dasatinib, Ibrutinib, and Fatostatin (MedChemExpress, Monmouth Junction, NJ, USA) were reconstituted in dimethyl sulfoxide (DMSO, Sigma-Aldrich, Saint Louis, Missouri, USA) for in vitro experiments. For in vivo experiments AZD1775 and Dasatinib were reconstituted in 5% DMSO, 40% PEG 300, 5% Tween 80, and 50% NaCl (all from Sigma-Aldrich) and stored in aliquots at − 20 °C. Alamar Blue assay was used to determine cell viability of the cells treated with increasing doses of drugs for 72 h. Cells (30,000 cells/well) were seeded into 96-well plates. For the recovery of proliferation assay, cells were treated for either 72 or 96 h and allowed to recover for an additional 10–14 days without the drugs. Regrowth was assessed by Alamar Blue staining. Fluorescence was measured at 570 nm by spectrophotometer. All experiments were repeated three times.

To assess the combined effects of AZD1775 and Dasatinib, Ibrutinib, Fatostatin, and AZD2014 (Drug 2) on cell viability, cells were treated with 0.5 μM of AZD1775 for 48 h, followed by the Drug 2 (1 μM) for an additional 48 h. As controls, cells were treated with AZD1775 or Dasatinib, Ibrutinib, Fatostatin, and AZD2014 alone for 96 h. Subsequently, the drugs were washed out and cells were allowed to recover for 10–14 days. Cell viability was measured using Alamar Blue assay. A reference point was established as the number of cells recovered after treatment with AZD1775 alone for 96 h, which was considered 100% recovery. The recovery of cells treated with the combination of AZD1775 and Dasatinib, Ibrutinib, Fatostatin, and AZD2014 was calculated relative to this reference, allowing for an assessment of the impact of the combined treatment on long-term cell viability. The experiments were performed in triplicate.

### Analysis of apoptosis using fluorescence live cell microscopy

Cells were stained with Incucyte® Caspase-3/7 Dye for Apoptosis (Sartorius) according to the manufacturer’s instructions, followed by live cell microscopy performed with an IncuCyte S3 Live Cell Analysis System (Essen Bioscience). Nine planes of view were collected per well, using the × 20 objective. The obtained data were analyzed with the IncuCyte S3 Cell-by-Cell Analysis Software Module (Essen Bioscience).

### Western blotting

For biochemical analyses of protein phosphorylation and total level, the cells were lysed in NP-40 lysis buffer (50 mM Tris–HCl pH 8.0, 150 mM NaCl, 1% NP-40) supplemented with both protease (Complete mini, Roche) and phosphatase (PhosSTOP, Roche) inhibitors. Proteins were resolved in 12% Bisacrylamide gel under reducing-denaturing condition, blotted onto PVDF membrane, and detected by immunoblotting using the appropriate antibodies. The list of antibodies and their sources can be found in the appendix file (Additional file 6, Table S6).

### Isolation of RUNX1 ubiquitin conjugates by TUBE pulldown

To detect ubiquitinated RUNX1, Nalm-6 cells overexpressing RUNX1 were used. RUNX1 expression was induced by adding doxycycline to the media 24 h prior to the experiment, followed by treatment with either AZD1775 or DMSO for 20 h before TUBE pulldown. Cells were lysed in NP-40 lysis buffer containing protease and phosphatase inhibitors, along with 100 mM N-ethylmaleimide (NEM). Lysates were incubated with anti-ubiquitin Agarose-TUBE 2 (Lifesensors, cat. no. UM402) for 1 h, according to the manufacturer’s protocol. Eluted proteins were subjected to immunoblotting with an anti-RUNX1 antibody (Abcam).

### In vitro ubiquitination assay

RS4;11 cells were treated with AZD1775 or DMSO for 20 h. Four hours before harvesting, cells were treated with 20 μM MG-132 to preserve multiubiquitin chains of RUNX1. Cell lysis was performed under denaturing conditions to disrupt non-covalent interactions. Total cell lysates were immunoprecipitated with 1 μg of anti-RUNX1 antibody (Abcam). Eluted proteins were then immunoblotted with an anti-ubiquitin antibody (Cell Signaling).

### Analysis of bulk microarray gene expression data in HEMAP

Normalized and log-transformed gene expression levels were compared between different hematologic malignancies or ALL subtypes from the HEMAP resource [39] based on the Wilcoxon test.

### PDX transplantation and in vivo drug treatments

In-house-bred NOD.Cg-*Prkdc*^*scid*^* Il2rg*^*tm1Wjl*^/SzJ (NSG) 8–9-week-old mice were sublethally irradiated (250 cGy) and 24 h later, transplanted through the tail vein with 2 × 10^6^ cells from MLL-7, a PDX established from a diagnostic sample from a childhood ALL with *KMT2A::AFF1* (courtesy of Dr. Richard Lock) [73]. For combination treatments, as a second cell model 2 × 10^6^ RS4;11 cells were transplanted to non-irradiated animals. Ciprofloxacin (KRKA, Stockholm, Sweden) was given in the drinking water for the duration of the experiment. A 1% engraftment of hCD45^+^hCD19^+^ cells was set as a starting point for the treatment and assessed by taking 60 µl of blood from *vena saphena* 14 days after transplantation and then every other day if the 1% of hCD45^+^hCD19^+^ was not reached. Red blood cells were lysed with ammonium chloride (Stemcell technologies, Cambridge, UK), washed in PBS (GE Healthcare Life Sciences, Logan, UT, USA) + 2% FBS (Thermo Scientific, Waltham, MA, USA), blocked with 10% mouse serum (#M5905, Sigma Aldrich) and stained with the following antibodies: anti-human CD45-APC (clone HI30, #555485, BD Bioscience, Franklin Lakes, NJ, USA) (hCD45) and anti-human CD19-APC/Cy7 (clone HIB19, #302217, Biolegend, San Diego, CA, USA) (hCD19). Dead cells were excluded with Draq7 (Biolegend). Flow cytometric analysis was performed using the LSRFortessa (BD Biosciences) and analyzed using FlowJo (FlowJo, LLC, Ashland, OR, USA). Once a 1% engraftment of hCD45^+^hCD19^+^ cells was achieved, mice (*N* = 3 per group) were either treated with 120 mg/kg of AZD1775 (MedChemExpress) 5 days a week, for 21 days (5 days on, 2 days off) (Additional file 1: Fig. S1d-h) for the survival experiment, or for 48 h with 120 mg/kg AZD1775 (MedChemExpress) every 24 h, and 4 h after the last dose, animals were sacrificed. For combination treatments, animals were treated with 6 days of AZD1775 (MedChemExpress) at 120 mg/kg alone or with Dasatinib for 3 days (MedChemExpress) at 50 mg/kg. Vehicle animals received 6 days of 5% DMSO, 40% PEG 300, 5% Tween 80, and 50% NaCl solution (all from Sigma-Aldrich). Animals were sacrificed by cervical dislocation 20 h after the last treatment. Before mice were sacrificed, a blood sample was taken from *vena saphena*. An autopsy was performed and bone marrow cells from the tibia, femur, and hip from both legs, as well as from the spleen, were collected and homogenized into single-cell suspensions by manual trituration and viably frozen in 10% DMSO (Sigma-Aldrich) in FBS (Thermo Scientific).

### GRO-seq assay

To analyze the actively transcribing RNA polymerases genome-wide, GRO-seq assays were performed as in [74]. Five million nuclei were collected from RS4;11 cells after 4, 10, and 24 h AZD1775 or DMSO treatment. The nuclear run-on reaction buffer (496 mM KCl, 16.5 mM Tris–HCl, 8.25 mM MgCl_2_, and 1.65% Sarkosyl (Sigma-Aldrich, Steinheim, Germany) was supplemented with 1.5 mM DTT, 750 μM ATP, 750 μM GTP, 4.5 μM CTP, 750 μM Br-UTP (Santa Cruz sc-214314A, Biotechnology, Inc., Dallas, Texas, USA and Sigma-Aldrich B7166), and RNAse inhibitors (RNase Inhibitor (Thermo Fisher, Carlsbad, CA, USA, and RNasin® Plus RNase Inhibitor Promega). To each 100 μl of nuclei samples, 50 μl of the run-on reaction buffer was added and incubated for 5 min at 30 °C. The RNA was collected using Trizol LS, fragmented and run-on reaction products were immuno-purified two times using anti-Br-UTP antibody (ab-6326, Abcam) bound to 30 μl of magnetic beads (Protein G DynabeadsThermo Fisher Scientific) and washed with 300 μl of PBST wash buffer four times (refer to detailed protocol in [75]). The cDNA template was PCR amplified (Illumina barcoding) for 12 cycles and size selected to 225–350 bp length. The ready libraries were sequenced with Illumina Hi-Seq2000 (GeneCore, EMBL Heidelberg, Germany).

### ChIP-seq assay

To distinguish the signal corresponding to active enhancer regions from the collected GRO-seq profiles, RS4;11 cells were similarly treated for 4, 10, and 24 h with AZD1775 or DMSO and cells were crosslinked with 1/10 of volume of 11% formaldehyde solution (37% formaldehyde (Sigma-Aldrich), 5 M NaCl, 0.5 M EDTA pH 8.0, 0.5 M EGTA, 1 M HEPES) for 10 min gently rotating at 2 mio/ml cell concentration in media. The reactions were quenched by adding glycine to a final concentration of 120 mM, and cells were washed twice with ice-cold PBS. Crosslinked lysate was flash frozen with liquid nitrogen and stored at − 80°C. Lysates were thawed, nuclei were extracted, and MNase-treated and antibody-bound chromatin collected by adding 25 μl of Dynabeads Protein G (Thermo Fisher Scientific) and rotating for 1 h at 4°C as previously described [74] with the following minor modifications: 5 U- 1U of MNase (Thermo Fisher Scientific) was used and 5 cycles sonicated then (Bioruptor® Plus and Bioruptor® Next Gen, Diagenode). Then supernatant was diluted to dilution buffer 2.5 times. Samples were pre-cleared with Protein G Dynabeads (Thermo Fisher Scientific) using 25 μl per sample (1 h 4°C rotating). Subsequently, input samples were taken. Five micrograms of the specific RUNX1 antibody (cat# ab23980, Abcam) for 5–16 million cells and 2.5 μg of specific H3K27ac antibody (cat# ab4729, Abcam) for 5–9 million cells were used. Beads were washed with 1 ml of cold wash buffers I, II, and III as in for 3 min [74]. (Finally, beads were washed twice with TE-buffer. Beads were eluted twice in elution buffer (1% SDS, 100 mM NaHCO_3_). Immunoprecipitated chromatin was reverse crosslinked by adding NaCl for a final concentration of 0.2 M and 0.4 mg/ml of RNAse A (Invitrogen) was added to each sample for 16 h at 65 °C. Proteinase K treated by adding 31 μl of Proteinase K buffer (161 mM EDTA, 645 mM Tris pH7.4, Proteinase K 0.65 mg/ml (Thermo Fisher Scientific)) for 1 h at 50°C. For DNA purification, ChIP DNA Clean & Concentrator (ZymoResearch, Irvine, CA, USA) was used like manufacturer’s protocol. ChIP-Seq libraries were prepared as previously described [74, 76] with samples PCR amplified for 13–14 cycles and size selected for 225–350-bp fragments by gel extraction. Single-end sequencing (75 bp) was performed with Illumina NextSeq 500/550 High Output Kit v2.5.

### GRO-seq read processing

GRO-Seq reads were quality controlled (FastQC). Reads were adapter trimmed using HOMER (version 4.9 [76] and filtered (min 95% of positions have a min phred quality score of 10) using the FastX toolkit (http://hannonlab.cshl.edu/fastx_toolkit/). Read mapping to rRNA regions (AbundantSequences as annotated by iGenomes) were discarded. The Bowtie software (version 1.1.2 [77]) was then used for alignment of remaining reads to the hg19 genome version, allowing up to two mismatches and no more than three matching locations. The best alignment was reported. Reads corresponding with so-called blacklisted regions that include unusual low or high mappability as defined by ENCODE, ribosomal and small nucleolar RNA (snoRNA) loci from ENCODE, and a custom collection of unusually high signal depth regions from GRO-seq was used to filter the data. GRO-seq tagDirectories were generated from aligned reads with fragment length set to 75 and data visualized using makeMultiWigHub.pl with strand-specificity HOMER (version 4.9).

### ChIP-seq read processing

Reads with poor-quality bases scores were filtered (min 97% of positions were required to have a min phred quality score of 10) using the FastX toolkit (http://hannonlab.cshl.edu/fastx_toolkit/). Duplicate reads were collapsed using fastx (collapse). Bowtie (version 1.2.3 [77]) was used to align the reads to the hg19 genome version, allowing up to two mismatches and no more than three matching locations. tagDirectories were generated with HOMER (version 4.9) tool. Histone peaks (GSE148195) [78] were identified using HOMER (version 4.9) findPeaks with setting -style histone. The peak signal profile across pooled tagdirs was then used to distinguish dips that correspond to nucleosome free regions that can be accessed by TFs (this was found to work well for peaks < 7.5 kb). To identify RUNX1 peaks, HOMER (version 4.9) findPeaks-style factor was used with the following less stringent settings: fold enrichment over input tag count 2, Poisson *p*-value threshold relative to input tag count 0.001, fold enrichment over local tag count 2, Poisson *p*-value threshold relative to local tag count 0.001, FDR 0.01, -tagThreshold 10. GM12878 NfKB (Tnfa treatment) from ENCODE SYDH ChIP-seq tagDirectory was generated from bam file using HOMER.

### GRO-seq gene signal analysis

To compare the effect of AZD1775 on primary transcript levels across the collected time points, Refseq (genome_annot_hg19 refGene_2018) coding and non-coding gene coordinates were quantified without exon regions. A raw count matrix was generated using Homer (version 4.9.1) specifying as maximum read per position 3 not allowing combining quantified reads if the same transcripts is detected more than one time. The low expression transcripts were filtered based on cpm and rpkm values requiring a row sum greater than 0.5 in at least two samples. To detect differentially expressed transcripts, a generalize linear model was fit using functions available in the R/Bioconductor package edgeR. Genes with significant change between any of the conditions (FDR < 0.001) were reported based on one-way ANOVA-like the likelihood ratio tested with glmLRT function. The genes were then clustered using k-means (into six clusters) and plotted as a heatmap (ComplexHeatmap R package) with six clusters. One cluster profile was excluded as it reflected mainly changes in DMSO basal levels between time points. Pathway analysis was performed on the gene clusters with > 100 genes; otherwise, the genes were plotted and examined individually.

### GRO-seq enhancer signal analysis

The nascent transcriptome result allows quantification of eRNA signal that can guide the analysis of TF activity. Enhancer regions are typically defined based on multiple genomic signal features. Here we started with DNA hypersensitive sites (DHS from Encode Duke narrow peaks, available across the ENCODE cell collection) and ATAC-seq peak coordinates (from RS4;11 cells in GSE117865 (GSM3681445) [79] and from bone marrow cell populations in [80]) for specifying candidate enhancer centers. The candidate regions were extended + / − 500 bp and the 1 kb size enhancer regions were quantified with Homer (homer/4.9.1) analyzeRNA. Coordinates with overlap with gene sense-transcription or the promoter region signal were excluded. Enhancers that passed a minimum cpm cutoff or intersected (bedtools2/2.27.1) with RS4;11 histone ChIP-seq peaks for H3K4me1 (GSE71616) [81] and H3K27ac (our own GSE148195 [78], GSE71616 [81] and GSE117865 (GSM3312817) [79]) were kept for analysis. Intergenic enhancers should have bidirectional and approximately equal signal from plus and minus strand. If the mean difference was over tenfold only the lower signal was considered (the stronger signal typically derives from transcribed gene region signal extending beyond annotated transcription termination site). For tightly clustered enhancers, the enhancer that overlapped best with RS4;11 ATAC or centered nuclear-free region (< 7.5 kb peak size) of H3K27ac peaks was selected, or otherwise the region with maximum signal was used. Quantified enhancers were filtered (expression cut-off 5 in minimum two samples) and normalization using RLE. A quasi-likelihood negative binomial generalized log-linear model was fitted using edgeR. The quasi-likelihood (QL) F-test was used to detect significant eRNA-level changes (FDR < 0.1). Clustering was performed using hierarchical clustering and distinct cluster profiles were visualized using the R package ComplexHeatmap. Denovo motif discovery was performed by extracting the DNA sequence for motif enrichment analysis with Homer (4.9.1) findMotifsGenome.pl (region size 200 bp, repeat masked genome, background all enhancers from statistical analysis). To further visualize enhancer signal, GRO-seq signal histograms were generated. For TF binding sites, intergenic region corresponding to motif-centered TF ChIP-seq peaks were used for signal summary. p53 ChIP peaks were retrieved from GSE46991 [82] and centered with TP53 motif (p63(p53)/Keratinocyte-p63-ChIP-Seq (GSE17611)/Homer). The histogram was generated with bin size 25 bp + / − 2000 bp from the ChIP-peak center. For chromatin state-specific enhancer analysis, regions that had high/low accessibility in each chromatin state were detected using the Seurat Findmarkers function, with a minimum fold change difference of 0.1 and FDR < 0.1. To further visualize top 200 chromatin state-specific enhancer signals the assay-specific signal matrixes were generated with annotatePeaks.pl tool of the HOMER package (version 4.11) [76]. The matrixes were generated with bin size 25 bp + / − 1000 bp from the enhancer center. Visualization was done with ImageJ (version 1.53t) [83] and average signal summary histograms were generated with the same parameters visualized with base R plots (R version 4.1.0).

### scRNA-seq assay from in vitro cell cultures

To analyze the drug response at cellular resolution RS4;11 or Nalm-6 cells were treated for 24 h with 1 µM AZD1775 or DMSO. Cell line samples from both the DMSO and AZD1775 treatments, representing the same parental line, were processed simultaneously. Cell viability was checked using Trypan blue with Cellometer Mini Automated Cell Counter (Nexcelom Bioscience), and only viable cells were processed further. To deplete dead cells, the Dead Cell Removal Kit (#130–090-101, MACS miltenyi Biotech) was used and the column rinsed twice with 1 ml Binding Buffer to elute viable cells (97–98% viability). Single-cell suspension, loading, and library preparation was performed according to the ChromiumTM Chromium Single Cell 3´Reagent Kits v3 User guide CG000184 Rev A and libraries constructed using the 10x Genomics Chromium technology. Loading concentrations were 1700 cells/µl, 1000 cells/µl for RS4;11 DMSO, AZD1775 cells; 500 cells/µl, 1300 cells/µl Nalm-6 DMSO, AZD1775, respectively. Each lane was loaded with 10,000 cells as pools of human and mouse cells (not part of this study) processed in parallel. Control and treatment samples were loaded onto the 10x Genomics Chromium controller and processed into libraries in parallel. Sequencing was performed in FIMM Technology Center Sequencing Laboratory, Biomedicum, Helsinki, Finland, by using a NovaSeq S2 sequencer aiming 50,000 reads per cell depth.

### scATAC-seq assay

RS4;11 cell nuclei were collected from parallel cell cultures as those used for the scRNAseq experiments to study chromatin accessibility at the single-cell level (scATAC-seq). Nuclei were isolated with 10x Genomics Nuclei Isolation for Single Cell ATAC Sequencing user guide CG000169 Rev D. Nuclei quality was determined with light microscoping with a × 40 focus. Nuclei concentration was determined with Trypan blue, and pools of human and mouse (not part of this study) cell lines were loaded together. scATAC-seq was performed using Chromium Single Cell ATAC Library & Gel Bead and Chip E Single Cell ATAC Kit (10 × Genomics), user guide CG000168 Rev C. Sequencing was performed in the FIMM Technology Center Sequencing Laboratory, Biomedicum, Helsinki, Finland, using NovaSeq S2 sequencer aiming 50,000 reads per cell depth.

### scMultiome-seq assay

To analyze the drug response transcriptional and chromatin accessibility from the same cells, RS4;11 were treated for 10 h with 1 µM AZD1775 or DMSO. In scMultiome assay nuclei were first isolated. 10x Genomics protocol (User guide CG000365 RevB) was first optimized for the RS4;11 cell line with a 3-min cell lysis time. Nuclei were then washed 3 times with 600* g* 7 min centrifugations steps. Nuclei quality was determined with light microscoping at × 40 focus. Nuclei count and lysis optimalization was determined with diluted Trypan blue solution with Countess 3 cell counter (Invitrogen). Each sample was loaded with 10,000 nuclei and library preparation performed according to the Chromium Next GEM Single Cell Multiome ATAC + Gene Expression User guide CG000338 Rev E and libraries constructed using the 10x Genomics Chromium technology. Loading concentrations were 7200 nuclei/µl for DMSO cells and 4860 nuclei/µl for AZD1775 cells. Multiome ATAC Sequencing was performed in Novogene Cambridge, UK, by using a NovaSeq PE50 sequencer aiming 20,000 reads per cell depth. Multiome RNA samples were sequenced in the same place with NovaSeq PE150 aiming 50,000 reads per cell depth.

### PDX cell staining and sorting for scRNA sequencing

Sample preparation for scRNA-sequencing involved thawing viably frozen bone marrow cells for WEE1 28 h time point inhibitor-treated mice and using fresh bone marrow cells for day 6 sequentially treated ones, which were stained with Draq7 (BioLegend), hCD45 (BD Bioscience), and hCD19 (BioLegend), together with a PerCP/Cyanine5.5 anti-mouse CD45 (BioLegend). Approximately 20,000 alive/mCD45^−^/hCD45^+^/hCD19^+^ cells were sorted into a PBS (GE Healthcare Life Sciences) + 2% FBS (Thermo Scientific) coated tube and prepared for sequencing using the 10x Genomics platform at the Center for Translational Genomics, Lund University.

### Single-cell genomics from primary ALL cells during chemotherapy

Bone marrow or blood mononuclear cells collected at diagnosis and 1 day following treatment start were analyzed from viably cryopreserved samples. Cells were thawn in a 37 °C water bath, immediately after adding 0.5 ml RPMI 1640 media (Thermo Fisher Scientific) + 10% FBS (Gibco) + 20 µl DNAse (Roche 100 U/µl) on top, then moving cells to 15 ml Falcon and filling the volume drop-wise, gently swirling. For samples with < 1 million cells, 10 µl of DNAse was added, filling volume to 5 ml. Cells were washed two times, then centrifuged and re-suspended in 50 µl Cell Staining Buffer (BioLegend) for counting. Subsequently, 100,000–200,000 cells per sample were processed by blocking with 5 µl of Human TruStain FcX Blocking Solution (BioLegend) for 10 min at 4 °C. Antibody pool (0.25 µg per million cells per specific antibody, except 0.125 µg for CD45 and CD8; 0.1 µg per hashtag antibodies) was added and incubated for 30 min at 4 °C. Samples were washed three times with cell staining buffer, counted and assessed for viability using trypan blue staining, and then sample pools were prepared, centrifuged, and loaded to Chromium lanes (10x Genomics). If post-stain viability was > 70% for all samples, equal ratios were loaded aiming at 10,000 cells. Otherwise, loading ratio was adjusted such that half target cell count was used for samples with viability 50–70% and one fifth of viability was 20–50%. The samples analyzed in this study (diagnosis EG9, day 2 EG19) are part of a sample set processed in three batches and with hashtag-barcoding of two donors per Chromium lane. The 5′ gene expression, ADT- and VDJ (BCR and TCR) libraries were prepared following manufacturer instructions (10x Genomics) and cDNA qualities were assessed using Bioanalyzer. Libraries were index barcoded and sequenced using Illumina Novaseq (S1/S2).

### scRNA-seq data processing and visualization

Cell line in vitro scRNA-seq data was processed and aligned with Cell Ranger (version 3.0.2) using hg19 and mm10 genome as reference. Human cell counts were used for downstream analysis. scRNA-seq data from mouse xenograft models were processed and aligned with Cell Ranger (version 6.0.1) using GRCh38-2020-A genome as reference. Primary ALL scRNA- and scADT-seq data was aligned with Cell Ranger 6.0 version to human reference (hg19) with default parameters. Donor (and singlet/doublet) assignment was carried out by DSB-normalized hashtag signals available in Seurat package and SNP-based donor assignment implemented in the tool vireo, keeping only concordantly assigned cells [84].

For downstream analysis, cells were log-normalized using a scaling factor of 10,000. Vst selection method was used to select 2000 highly variable features (genes) and from these PCA dimension 1:10 were used for neighborhood analysis (FindNeighbors function), clustering (FindClusters with resolution parameter varied from 0.5 to 2) and dimensionality reduction (UMAP). The respective parameters are specified in the accompanying code repository and resulting cell and cluster numbers in Additional file 3, Table S3. At 24 h, RS4;11 DMSO and AZD1775 samples were initially merged and filtered in the same manner. From the RS4;11 cell line in vitro sample, this resulted in a discovery of 11 distinct cell clusters from the merged object with DMSO and AZD1775 treatment (24 h) that are referred to as “cell states” and used as reference sample for label transfer analysis (see below). For cell cycle characterization, a list of cell cycle markers was utilized from [85].

Several statistical comparisons between the cells assigned to these cell states were performed: (i) Genes that had high/low expression in each cluster were detected using the Seurat Findmarkers function, with a minimum fold change difference of 0.25 between cells assigned to the cluster and other cells. (ii) AZD1775-treatment specific cell states were compared with the cell state matched to normal cell cycle G2/M-phase (cell state 4), (iii) cluster markers were detected between AZD1775-treatment specific cell states. Pair-wise correlation was also calculated for percentage of expression data (obtained from R dittoSeq version 1.6.0 dittoDotPlot function) to analyze TF co-expression at cluster level. This analysis was performed with rcorr function with Pearson correlation. Violin plots were used to visualize gene expression distributions. For visualizing gene expression level per cell cluster dotplots were generated using the dittoSeq package. Alternatively, all unique genes from cluster comparisons were acquired and the mean scaled counts were summarized. These mean expression values were then used as input for hierarchical clustering with 1-Pearson correlation as a distance metric. ComplexHeatmap R package was used for visualizations, testing different cluster numbers (10–20) for row-wise clustering. Pathway analysis was performed for the gene clusters and for broader patterns distinguished based on the heatmap.

### scATAC-seq data processing and visualization

The raw reads were processed and aligned with Cell Ranger atac workflow (version 1.2.0). Seurat ([86], version 3.1.1) package and Signac ([87] version 0.1.6) were used for downstream analysis, selecting only the human peak coordinates and cells. Peaks detected from DMSO- and AZD1775-treated cells were merged using bedtools (version 2.27.1), discarding < 10 bp wide peaks. Data across treatments was combined by re-quantifying counts from the fragment files based on the merged peak regions. Peaks detected in > 1% of cells were kept for downstream analyses. Nucleosome signal was quantified based on fragments mapping to chr 1 using the Signac package NucleosomeSignal function. TSS enrichment was calculated based on coordinate ranges retrieved from EnsDb.Hsapiens.v75. These quality metrics were used in combination with metrics to select good quality nuclei for downstream analyses (peak region fragments > 1000, peak region fragments < 100,000, percentage reads in peaks > 15, blacklist ratio < 0.05, nucleosome signal < 10, TSS.enrichment > 2). To analyze the atypical nucleosome signal manifest in AZD1775-treated sample, the above filtering criteria were used without the nucleosome signal cutoff to plot the signal profiles. Term frequency-inverse document frequency (TF-IDF) normalization of peaks by accessibility was performed using the LSI implementation used by [87]. To reduce dimensions and perform clustering of the data matrix, singular value decomposition (SVD) was run on the TD-IDF normalized matrix, keeping 150 dimensions. Based on elbow plot analysis 50 dimensions were kept for UMAP visualization and clustering.

To study more specifically chromatin activity responses at each treatment condition, separate UMAPs were created (reduction method lsi, dims = 1:50, resolution 0.4). The two smallest chromatin states found in the chromatin state clustering of DMSO- (c5, c6) and AZD1775- (c6, c7) treated cells differed based on quality control metrics. The smallest clusters had a low peak region fragment amount (low quality) and were discarded from all downstream analyses. The clusters with elevated nucleosomal signal were reproduced in the scMultiome result and could be matched using this data to cells with a good quality RNA-seq profile. Moreover, they had a distinct TF motif activity pattern in chromVAR analysis (unlike the smallest cluster). However, in visualizing TF activity scores specific to other clusters, we omitted the clusters with elevated nucleosome signal from heatmaps since they consistently showed low signal. For chromatin state-specific enhancer analysis, we started with intergenic enhancer defined from bulk genomics data and identified high/low accessibility regions in each chromatin state using the Seurat Findmarkers function, with a minimum fold change difference of 0.1 and FDR < 0.1. To further visualize top 200 chromatin state-specific enhancer regions, the assay-specific pseudobulk signal matrixes were generated by creating a combined tagDirectory for cells matched to each chromatin state, followed by signal quantification with annotatePeaks.pl tool of the HOMER package (version 4.11 [76]). The matrixes were generated with bin size 25 bp + / − 1000 bp from the enhancer center. Visualization was done with ImageJ (version 1.53t [83]) and average signal summary histograms were generated with the same parameters visualized with base R plots (R version 4.1.0).

### scMultiome-seq data processing and visualization

For comparison with the 24 h data, multiome scRNA-seq data was processed with the same Cellranger preprocessing workflow, resulting in reads mapped to the hg19 genome version. DMSO- and AZD1775-treated samples were merged and analyzed using Seurat v4.1.1. The raw reads were also processed and aligned with Cell Ranger Multiome workflow (cellranger-arc-2.0.1, hg38 genome version). The cell assignment available from per barcode metrics output was used to select nuclei for downstream analyses. The scATAC-seq workflow based on Signac [87] 0.1.6 and Seurat 3.1.1. versions that was used for 24 h data was run with minor modifications (EnsDb.Hsapiens.v86, no blacklist filtering, atac_peak_region_fragments > 1000 & atac_peak_region_fragments < 100000 & pct_reads_in_peaks > 50 & nucleosome_signal < 10) to generate comparable results. Nucleosome signal histogram analyses were performed using Signac version 1.6.0. The matching cell barcodes between scRNA- and scATAC-seq profiles from the same cells were utilized to visualize data across modalities. For example, this allowed showing the scATAC-seq nucleosome signal on the UMAP generated from scRNA-seq data. Cell type labels were assigned based on label transfer analysis with the respective 24-h sample used as reference sample.

### scRNA-seq RNA velocity and PAGA analysis

Dynamic changes in gene transcription can be modeled based on reads corresponding to both unspliced and splice mRNA (newly synthetized RNA vs processed RNA, respectively). Based on the dynamic RNA processing model [88], the future transcriptome state can be visualized together with the measured current state. This analysis was used to analyze cell state dynamics in the RS4;11 cell model. Velocyto CLI (version 0.17.17 [88]) was used to calculate the count matrices, masking expressed repetitive elements (available for hg19 from UCSC Genome Browser). The scVelo-package (version 0.2.1 [89]) was used in downstream analysis. The gene expression matrix was accompanied with the spliced and unspliced count matrices. The data was first filtered by removing genes with less than 20 shared counts in both spliced and unspliced data. The matrices were each then normalized by dividing the counts in each cell with the median of total counts per cell. The 2000 most variable genes were extracted based on the spliced count matrix and the data matrices were log-transformed. Top PCs (30) were used for neighborhood graph calculation, with the number of neighbors set to 30. Then the AZD1775 and DMSO samples were separated for RNA velocity analysis. Based on the neighborhood connectivities, the first-order moments for spliced and unspliced matrices were calculated and the velocity was estimated using the dynamical model. The velocities were embedded to a UMAP presentation, which was calculated with 20 PCs and 10 neighbors. A PAGA graph was used to visualize connectivities (dashed lines) and transitions (solid lines). Python Scanpy-package (version 1.5.1) was used for gene set scoring. Before scoring, gene sets used in the analysis (e.g. senescence gene sets) were filtered to include only genes that are expressed in the data, excluding G2M-phase markers (to remove cell cycle bias in the scores).

### TF motif activity profile from scATAC-seq profile

To determine motif activity, i.e., variability in chromatin accessibility per cell, the chromVAR ([90], version 1.6.0) tool was applied separately to each sample. The known TF motifs available in the Homer tool were scored. Subsequently, cluster-specific motifs were detected using statistical analysis (Seurat FindMarkers function, log2 fold change threshold 1.5, FDR < 0.0001) comparing each chromatin cluster to other clusters. Redundant motifs recognized by similar protein complexes were grouped manually and the most significant motif kept for visualization. The scaled chromatin accessibility values were used to calculate mean access per chromatin cluster and visualized using the ComplexHeatmap R package. The Signac visualization functions were used to generate genome browser track plots comparing different clusters.

### Data integration of single-cell genomics samples using label transfer

To characterize similar cells between two different sample sets, the canonical correlation analysis available in the Seurat (Seurat 4.1.1) package was used. First anchors between two different samples were identified following the user guide recommendations. For cell line and PDX data, the 24 h RS4;11 data cell states 1–11 were used as reference. These cell state labels were transferred to the 10 h multiome scRNA-seq data, or the PDX data. Similarly, the MLL-7 clusters from 28 h were transferred to day 6 sample data. Prediction scores were visualized for quality control purposes. The same analysis was performed to characterize chromatin levels with user guide recommendations for ATAC data. Twenty-four-hour RS4;11 DMSO ATAC clusters 0–5 were used as reference for 10 h DMSO multiome scATAC-seq data. Twenty-four-hour RS4;11 AZD1775 ATAC clusters 0–6 were used as reference for 10 h AZD1775 multiome scATAC-seq data. Cells with prediction scores < 0.3 were omitted from visualizations (assigned to category NA). Proportion plots of label assignments were visualized using the R Dittoseq package (version 1.6.0) with the dittoBarPlot command. To illustrate the correspondence between 24-h clusters and 10-h clusters, we generated Sankey diagrams using the 10-h multiome samples treated with DMSO and AZD1775. We employed the R package scmap (version 1.16.0) and getSankey function. Rare categories of labels (assigned to < 1.5% cells analyzed) were omitted from the plot for clarity.

### Pathway enrichment analysis

Gene lists acquired bulk genome-wide and scRNA-seq studies were analyzed using the online web server Enrichr [91] for enrichment of ontology and pathway terms. The analysis was performed based on gene sets from BioPlanet 2019 and TF perturbations. Enriched terms were ranked based on the lowest FDR and top terms per group visualized as dot plots (R package ggplot2).

### Cell proliferation, EdU incorporation, and senescence assessment by FACS

To study the effects of AZD1775 on cell proliferation, cells were incubated with 10 μM EdU for 1 h at 37°C to label proliferating cell population. EdU-labelling of the cells was done prior to the treatment for further processing according to the kit protocol (Thermo Fisher). Following cell fixation and permeabilization, the cells were stained with pH3 (1:25, BD Bioscience), and 50 μg / mL Propidium Iodide in 1% BSA/ TBS – 0.5% Tween 20 containing 10 μg/mL RNAseA overnight at 4°C. Finally, the click-iT reaction were carried out according to the manufacturer’s protocol prior to FACS analysis. To analyze senescence, treated cells were or CellEvent Senescence Green Flow Cytometry Assay Kit (Invitrogen, C10840) and Propidium Iodide according to the manufacturer’s protocol.

### Quantitative real-time PCR (qRT-PCR)

To perform the qRT-PCR, RNA was prepared using RNeasy kit (Qiagen). Residual genomic DNA was eliminated from the total RNA fraction by DNase I treatment (Qiagen), according to the manufacturer’s protocol (Qiagen). Fifty nanograms of the purified RNA was then used for cDNA synthesis (SuperScript VILO cDNA Synthesis Kit, Invitrogen, Life Technologies). Quantitative PCR were carried out using SYBR green probes as described in the appendix file (Additional file 6, Table S6). The data were analyzed with ΔΔCt method and presented as relative expression.

### Statistical analysis

Data from biochemical assays are presented as the mean + SD from two or more independent experiments unless indicated otherwise. Statistical analyses were performed using the statistical package GraphPad Prism 8 (GraphPad Software, San Diego, CA, U.S.A., http://www.graphpad.com) using either Student’s *t* test, Log-rank (Mantel-Cox), one-way analysis of variance (ANOVA), or two-way analysis of variance (ANOVA), as indicated. Genome-wide statistical analysis was performed using dedicated software (R or Python packages as indicated). Multiple testing correction was performed using Benjamini–Hochberg or Bonferroni’s post hoc test when appropriate.

### Supplementary Information

**Additional file 1. **Contains supplementary figures S1-S10 and their legends. Contains data set access codes and Table S1.

**Additional file 2. **Table S2 GRO-seq summary of significantly differentially expressed genes. Differentially expressed genes based on one-way ANOVA-like statistical comparison of mean transcriptional activity levels across sample conditions and summary of pathway enrichment analysis.

**Additional file 3. **Table S3 Summary of scRNAseq samples and cluster marker gene statistical analyses. Data filtering: scRNAseq sample filtering cutoffs and number of cells used in analysis. Differentially expressed genes from comparison of AZD1775-specific clusters (cell states 5-11) and DMSO cell state 4 (matching G2/M cell cycle phase) from 24 h time point in RS4;11 cells, heatmap cluster assignment (refer to Fig. S2e) and summary of pathway results. Marker gene lists for 24 h time point RS4;11 cell AZD1775-specific clusters. List of cell fate III top markers. In vivo samples: MLL-7 PDX AZD1775 treatment (28 h) cluster 6 marker genes and summary of pathway analysis; MLL-7 PDX AZD1775 treatment (day 6) corresponding pathway analysis for cells matched to 28 h cluster 6; RS4;11 in vivo marker genes for 11 clusters of data merged across treatments.

**Additional file 4. **Table S4 scATAC summary of significantly different motif access across clusters. RS4;11 24 h AZD1775 and DMSO chromVar analysis results. Significantly differentially active motifs used for clustering. Based on statistical comparison of AZD1775 and DMSO chromatin states (data sheets chromatin state 1-6), and DMSO chromatin state 1-5, p-value from Wilcoxon Rank Sum test is shown.

**Additional file 5. **Table S5 GROseq summary of enhancer analysis. Up-regulated enhancers at 24 h: top enriched TF motifs for the corresponding DNA sequences.

**Additional file 6. **Table S6 Primer and antibody summary. List of RT-qPCR primers and antibodies used in the study.

**Additional file 7. **Contains original images of uncropped western blots.

**Additional file 8. **Review history.

## Data Availability

Processed data from genomics assays are available via the NCBI Gene Expression Omnibus database under the following accession codes: GSE220099 (bulk GRO-seq profiles in the RS4;11 cell line) [92], GSE148195 (bulk ChIP-seq data in RS4;11 cell line) [78], GSE218621 (scRNA-seq data in RS4;11 and Nalm-6 cell lines) [93], GSE220476 (scRNA-seq data from mouse xenografts of RS4;11 and MLL7 cells) [94], GSE230295 (scRNA-seq data from primary ALL patients) [95], GSE218805 (scATAC-seq data in RS4;11 cell line) [96], and GSE220112 (sc-Multiome data in RS4;11 cell line) [97]. Additional data used in enhancer and ChIP-seq peak analysis is available under the accession codes GSE148192 (RUNX1 in Nalm-6 cell line) [98], GSE71616 (histone markers in RS4;11 cell line) [81], GSE117865 (chromatin accessibility and histone marker data in RS4;11 and SEM cell lines) [79], and GSE46991 (p53 binding sites in LCL) [82]. Code related to analyses is available at GitHub [99] and Zenodo [100] under GNU General Public License 3.0.
