# Supplementary material for: Sequential drug treatment targeting cell cycle and cell fate regulatory programs blocks non-genetic cancer evolution in acute lymphoblastic leukemia
Source: Genome Biol. 2024 May 31;25:143. doi: 10.1186/s13059-024-03260-4 (PMC11143599; doi:10.1186/s13059-024-03260-4)
Supplement: Supplementary file 1 — Additional file 1. Contains supplementary figures S1-S10 and their legends. Contains data set access codes and Table S1. [file 13059_2024_3260_MOESM1_ESM.pdf]

Additional file 1

Sequential drug treatment targeting cell cycle and cell fate regulatory programs blocks non-genetic cancer evolution in acute lymphoblastic leukemia

Alena Malyukova[1*†], Mari Lahnalampi[2†], Ton Falqués-Costa[3], Petri Pölönen[2], Mikko Sipola [2], Juha Mehtonen [2], Susanna Teppo[4], Karen Akopyan[1], Johanna Viiliainen[1], Olli Lohi[4], Anna K Hagström-Andersson[3], Merja Heinäniemi[2*†], Olle Sangfelt[1*†]

1. Department of Cell and Molecular Biology, Karolinska Institutet, Stockholm, Sweden
2. The Institute of Biomedicine, School of Medicine, University of Eastern Finland, Kuopio, Finland
3. Division of Clinical Genetics, Department of Laboratory Medicine, Lund University, Lund, Sweden.
4. Tampere Center for Child, Adolescent and Maternal Health Research, Faculty of Medicine and Health Technology, Tampere University and Tays Cancer Center, Tampere University Hospital

† Equal contribution

* Corresponding authors

Table of contents

Data set access codes

Supplementary table 1

Supplementary figures S1-S10 and their legends

Data set access codes

Superseries:	GSE220114
GSE220099	GRO-seq RS4;11 time course
GSE218621	scRNA-seq RS4;11 and Nalm6 24 h
GSE148192	scRNA-seq Nalm-6 (DMSO) and ChIP-seq
GSE218805	scATAC-seq RS4;11 24 h
GSE220112	scMultiome-seq RS4;11 10h
GSE220476	scRNA-seq PDX
GSE148195	ChIP-seq RS;411
GSE230295	scRNA-seq pediatric ALL (incl. MEF2D)

Table S1. GI50 values of cell line models. Related to Fig. 1.

Cell line	GI50 (nM)
T-ALL	266
Molt16	94
Peer	390
647	242
RCH-ACV	466
Kasumi-2	266
MHH-Call3	176
Sup-B15	321
TMD5	640
RS4;11	337
SEM	281
ALL-PO	320
KOPN8	440
Nalm6	331
REH	630

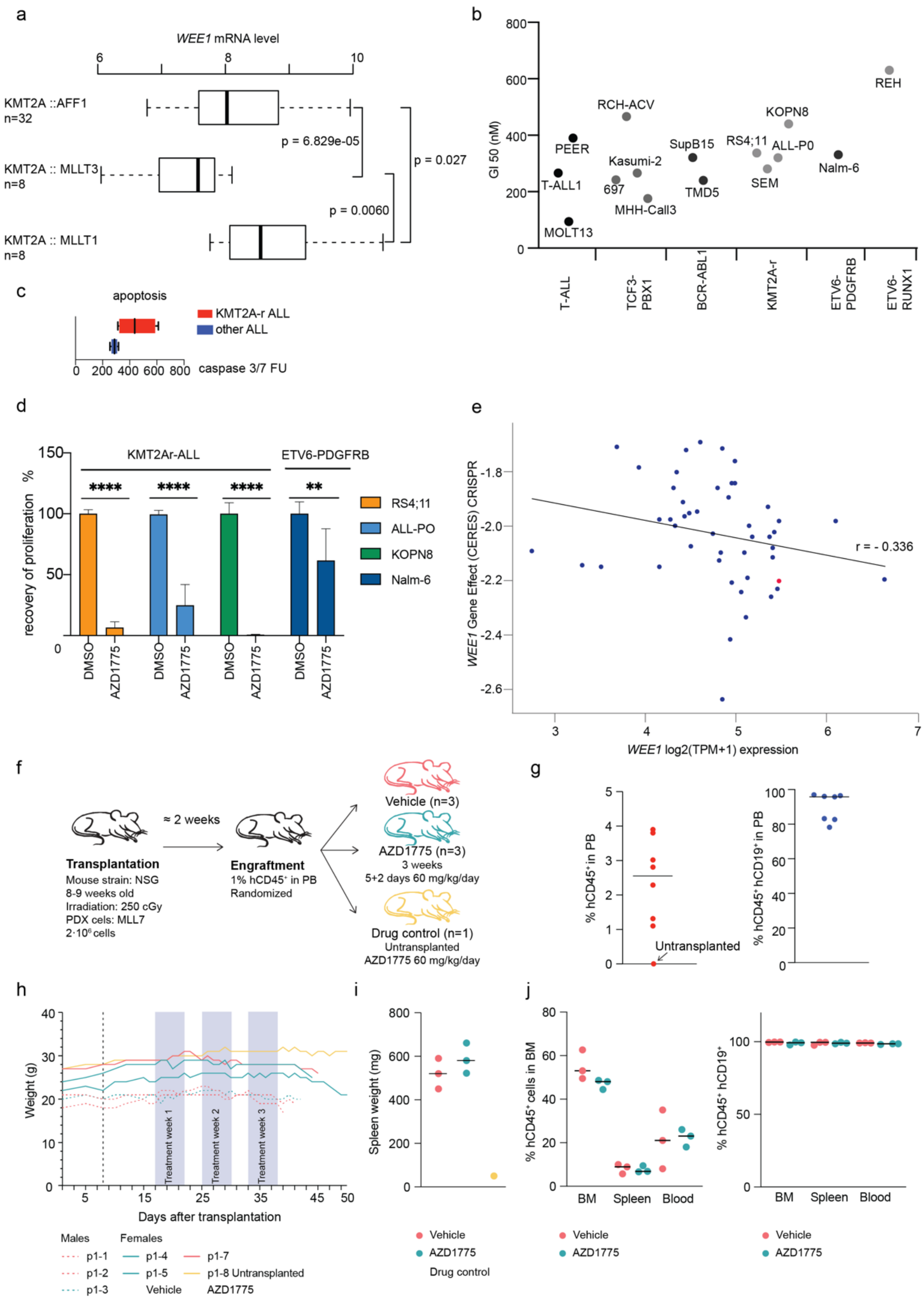

Fig. S1, related to Fig. 1.

a. Boxplots of *WEE1* expression level in different KMT2A fusions (HEMAP dataset). Wilcoxon test p-value is indicated.

b. GI50 values of AZD1775 treatment (72 h) comparing ALL cell lines corresponding to different genetic subtypes.

c. Box plots comparing Caspase3/7 activity in response to AZD1775 treatment in KMT2A-r and non-KMT2A-r ALL cell lines. Data are represented as mean ± SD.

d. Recovery of cell proliferation following removal of AZD1775. Cells were treated for 72 h with AZD1775 and allowed to recover for an additional 10 days without the drugs. Regrowth was assessed using Alamar Blue stainings.

** denotes $p<0.01$ and **** denotes $p<0.0001$ determined using Student's t-test. Data are represented as mean ± SD.

e. Scatter plot visualization of WEE1 gene dependency based on CERES score (from DepMap) against *WEE1* mRNA level in hematopoietic cell lines. A lower CERES score indicates higher probability of dependency (0: not essential, -1 median of all common essential genes). KMT2A-r ALL cell line (SEM) is indicated in red color.

f. Schematic representation of the experimental plan depicting the different treatment arms.

g. Scatter plot showing the leukemic cell engraftment 1 day after treatment start, represented by the fraction of hCD45+CD19+ cells.

h. Time course of mouse weight measurements (every other day before treatment and daily afterward). Treatment was well tolerated.

i. Spleen weights at sacrifice.

j. Flow cytometric quantification of leukemia burden, showing the fraction of hCD45+ and hCD19+ cells.

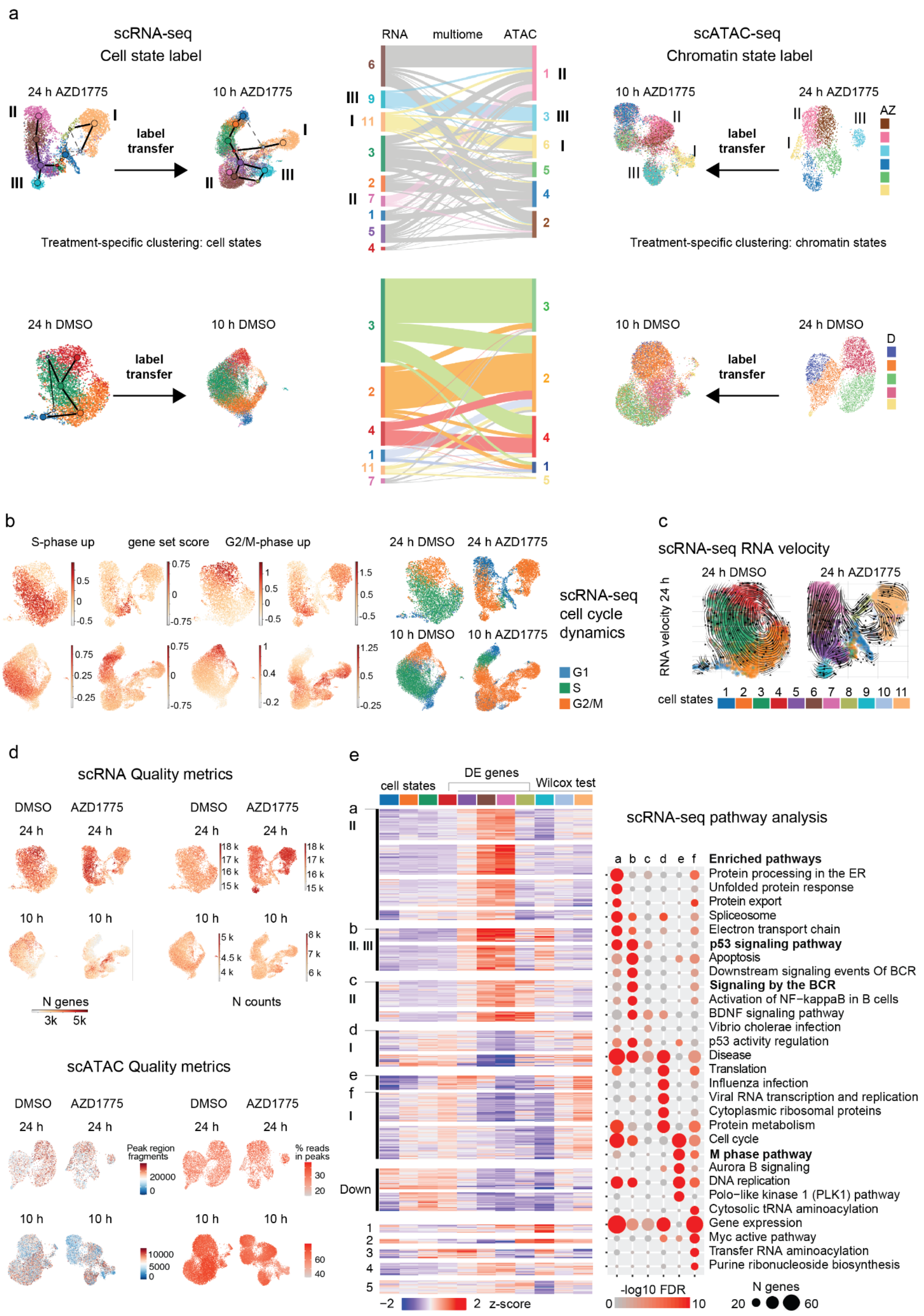

Fig. S2, related to Fig. 2.

a. Correspondence between 24 h clusters and 10 h data, as assigned by label transfer performed by assay, is indicated on the UMAPs. The Sankey plot shows how the labels assigned based on RNA profile compare to those assigned by ATAC profile for the 10 h multiome samples where each cell barcode has data for both modalities. Cell fates (I, II, III) upon treatment are highlighted.

b. UMAPs showing gene set scores for S- and G2/M phase specific genes and cell cycle phase assignment.

c. Cell state dynamics based on RNA-velocity analysis from 24 h scRNA-seq. The colors correspond to assigned cell state based on 24 h scRNA-seq data, refer to cell states shown in Fig. 2d. Arrows point towards the predicted future state of each cell.

d. UMAPs showing scRNA-seq (N counts: number of counts, N genes: number of genes) (top) and scATAC-seq quality metrics (peak region fragments, percentage of fragments in peaks) (bottom). Red color tones indicate higher values.

e. Differentially expressed genes from comparison of AZD1775-specific clusters (cell states 5-11) and DMSO cell state 4 (matching G2/M cell cycle phase) shown as a heatmap where genes (in rows) are clustered based on their mean expression in cells corresponding to each cell state. The color corresponds to z-score. Pathway analysis was performed based on broader similarity of clusters (indicated by letters a-f) and shown as dot plot as in Figure 2c. Small clusters (<100 genes) with specificity to cell state 9 are indicated at the bottom. For complete gene to cluster assignment, refer to Table S3.

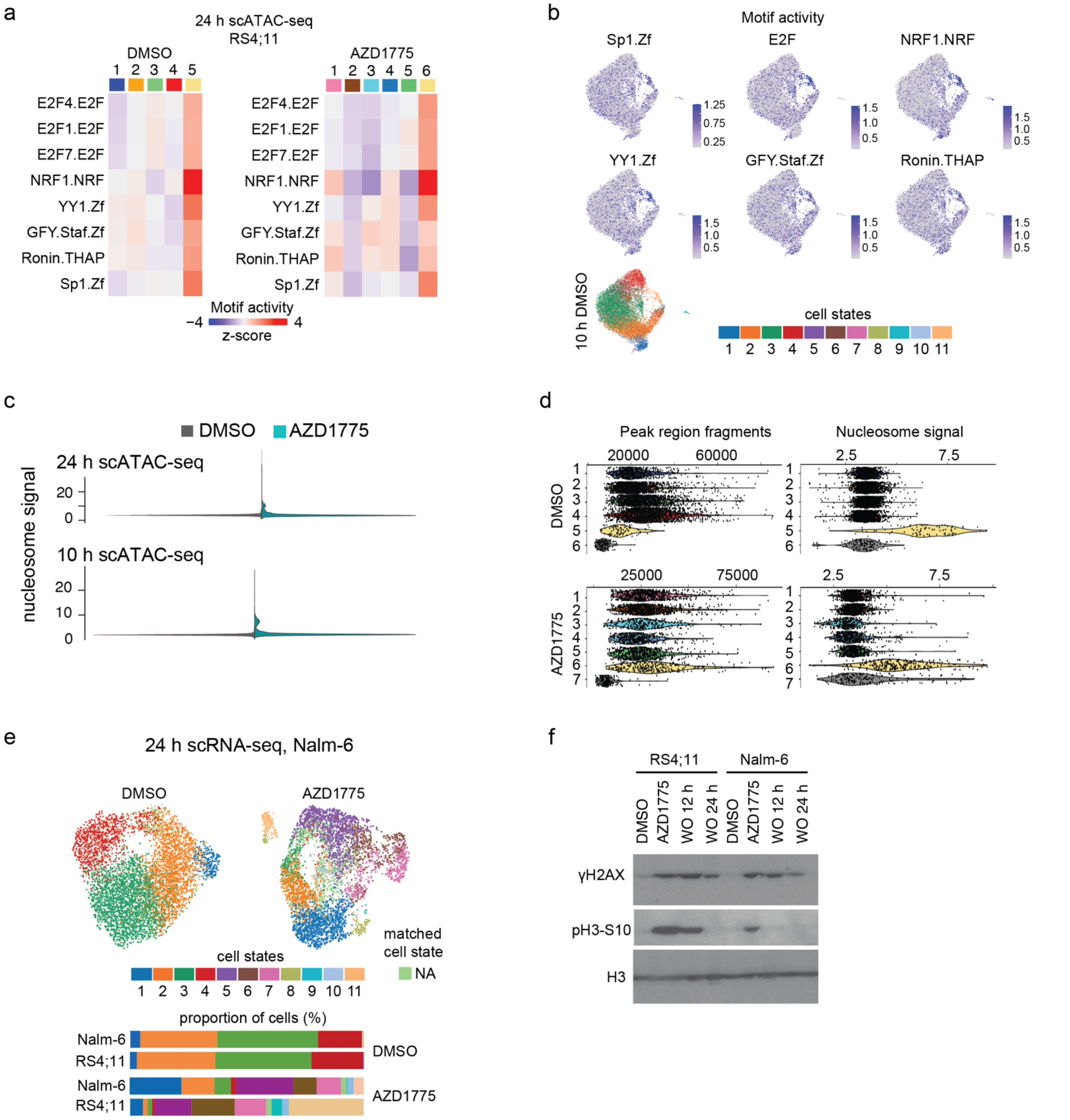

Fig. S3. related to Fig. 3.

a. Heatmap showing TF motif activity score across chromatin states in DMSO and AZD1775 treated cells (scATACseq, 24 h). Motifs with high chromatin access in the clusters with high nucleosome signal (see panel S3d) corresponding to chromatin state 5 (DMSO: left) and chromatin state 6 (AZD1775: right) are shown. Related to Fig. 3a-b.

b. TF motif activity score visualized for TFs shown in B on 10 h DMSO scRNA-seq UMAP, as in Fig. 3a.

c. Nucleosome signal visualized as violin plot from scATAC 24 h and 10 h data filtered based on other quality metrics (See methods). Grey: DMSO treated cells. Green: AZD1775-treated cells. Final analysis was limited to cells with signal <10.

d. Quality metrics compared between chromatin state clusters identified from scATAC 24 h data. Initial clustering based on the neighborhood graph obtained from LSI-reduced scATAC- seq signal resulted in 6 (DMSO) and 7 (AZD1775) clusters. From both treatments, cells with lowest peak region fragments were removed from further downstream analysis. Condensed chromatin state corresponds to clusters 5 (DMSO) and 6 (AZD1775).

e. scRNA-seq transcriptome based UMAP visualizations (top) of Nalm-6 cells treated with DMSO or AZD1775 are shown. Colors correspond to matching RS4;11 cell state. NA: cells with prediction score < 0.5. Cell states 6 and 7 correspond to highest p53 activity. Comparison of cell state proportions based on scRNA-seq analysis of RS4;11 and Nalm-6 cell lines is shown (bottom).

f. Immunoblot analysis of γH2AX and pS10-H3 in response to AZD1775 and after drug washout (WO) for 12 and 24 hrs in RS4;11 and Nalm-6 cells, respectively. Histone 3 serves as a loading control.

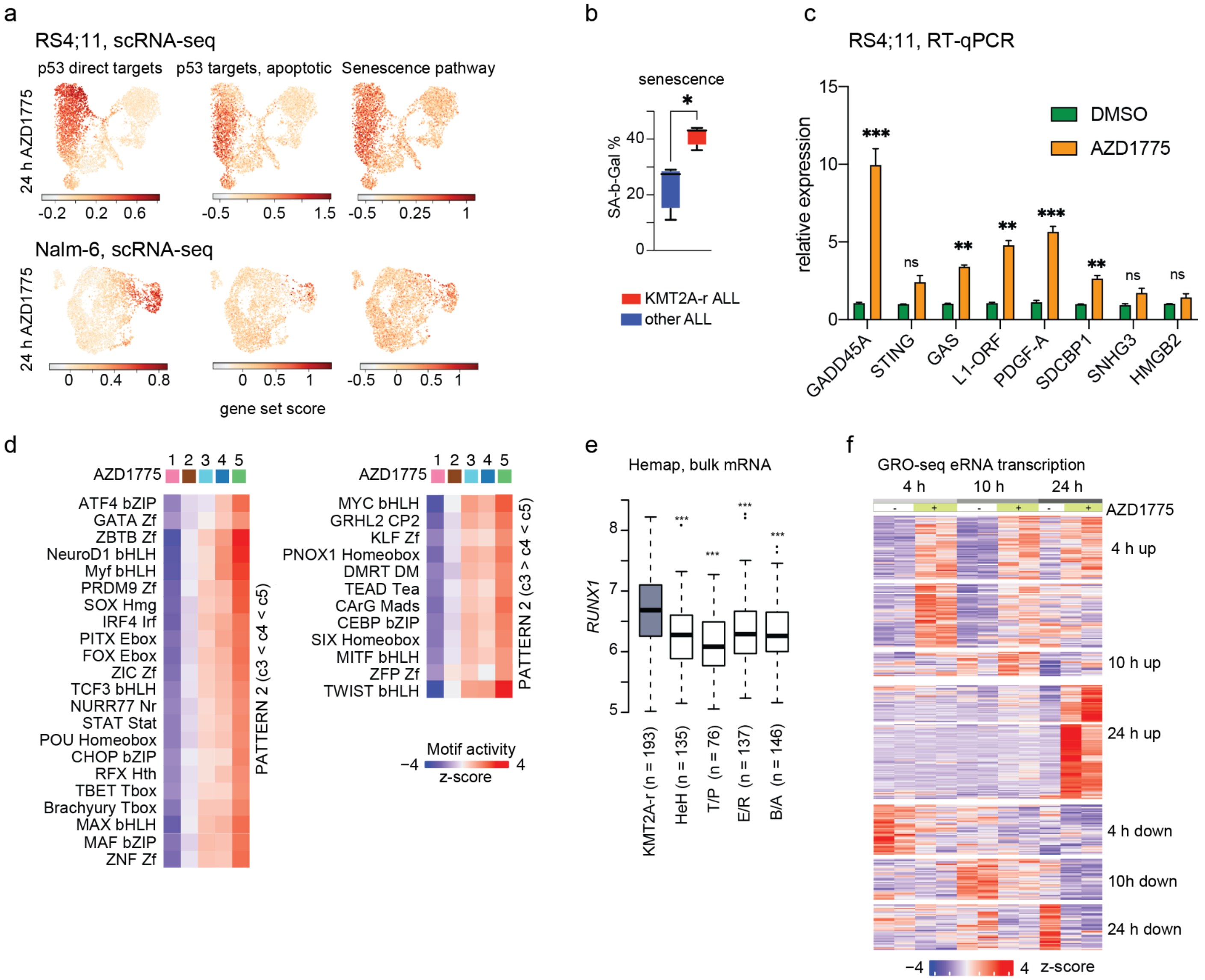

Fig. S4 related to Fig. 4.

a. Gene set score for P53 direct target genes is visualized on the scRNA-seq UMAP for RS4;11 (top) and Nalm-6 cells (bottom) treated with AZD1175 for 24 h.

b. Box plot visualization of the percentage of senescence associated-β-Galactosidase (SA-β-Gal)-positive cells following 72 hr of AZD1775 treatment and 10 days drug wash-out comparing KMT2A-r and non-KMT2A-r ALL cell lines. * indicates p=0.0167 determined using t-test. Data are represented as mean ± SD.

c. qPCR analysis of senescence-associated secretory pathway (SASP) genes. mRNA expression levels of the indicated genes at 48 h of AZD1775 treatment is shown adjusted to the *GAPDH* mRNA and relative to the DMSO control. ** denotes p<0.01 and *** denotes p<0.001 determined using Student's t-test. Data are represented as mean ± SD.

d. Heatmap showing TF motif activity (scATAC, 24 h) across chromatin states in AZD1775 treated cells for additional TF motifs in Pattern 2 (see Fig. 4a).

e. Boxplots comparing *RUNX1* gene expression in B-ALL subtypes based on HEMAP data from diagnostic patient samples. Wilcoxon test p-value from KMT2A-r vs each other subtype is indicated (*** : p-value < 0.001).

f. Heatmap illustrating the magnitude and direction of changes in the GRO-seq eRNA signal (z-score; tones of red indicate high level, tones of blue low level). Top enriched TF motifs for the corresponding DNA sequences are listed in Table S5.

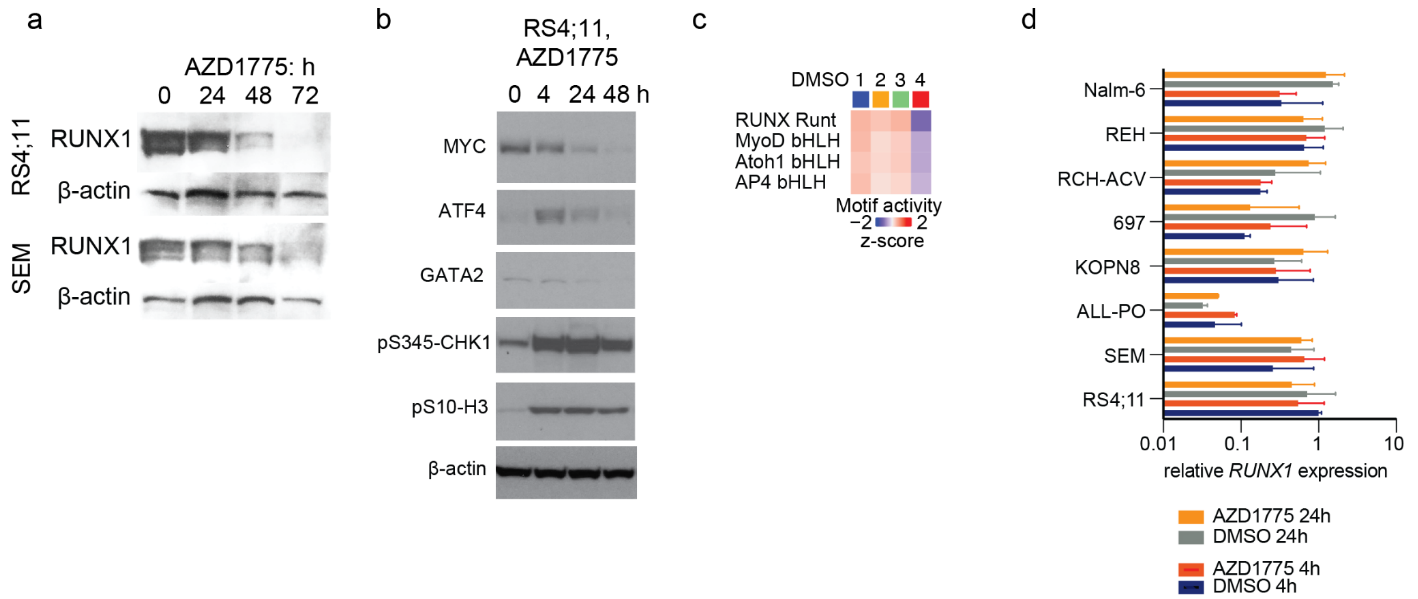

Fig. S5 related to Fig. 5.

a. RUNX1 protein levels in RS4;11 and SEM treated with AZD1775 or DMSO for indicated time points were analysed by immunoblotting. β-actin served as a loading control.

b. Whole cell lysates of RS4;11 cells treated with AZD1775 for the indicated time points and immunoblotted with antibodies against MYC, ATF4, GATA2, pCHK1, pS10-H3. b-actin is a loading control.

c. Motifs with similar profile to RUNX1 (refer to Fig. 4a) visualized as heatmap from DMSO-treated cells (24 h). Chromatin states 1-4 identified from DMSO scATAC-seq data are shown. Cluster 1 matched G1 and S-phase cells, see Fig.S2a.

d. *RUNX1* mRNA expression levels at 4 h and 24 h treatment (DMSO compared to AZD1775) was analysed by quantitative PCR (qPCR). Relative *RUNX1* expression levels were adjusted to the *GAPDH* expression.

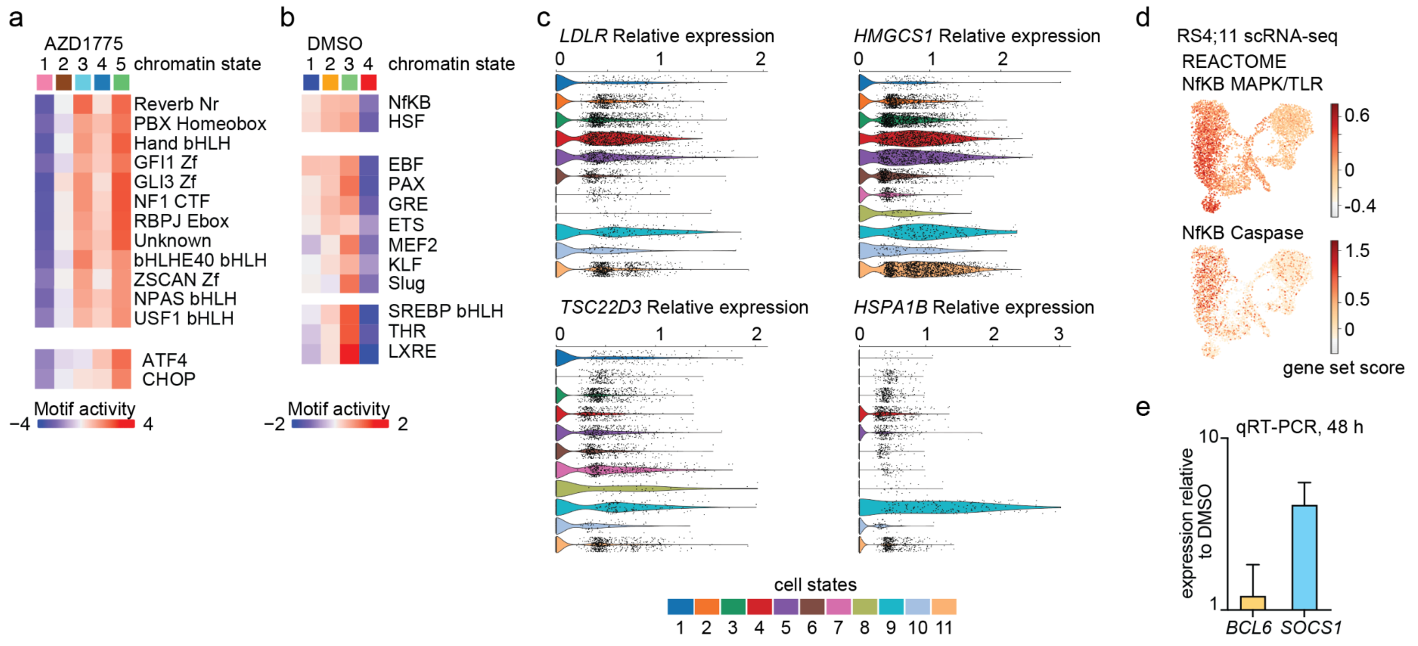

Fig. S6 related to Fig. 6.

a-b. TF motif access across 24 h AZD1775 (A) and DMSO (B) chromatin states is shown in the heatmap for TFs with high activity in AZD1775 chromatin state 5 (see Fig. 6a). Condensed chromatin state clusters with overall low signal are omitted from the heatmap.

c. *LDLR*, *HMGCS1*, *TSC22D3* and *HSPA1B* expression level is shown from scRNAseq 24 h as violin plots comparing cell states 1-11. The track color corresponds to cell state annotation.

d. Gene set score for public gene sets related to NfKB MAPK/TLR and NfKB caspase targets visualized on the UMAPs in 24 h AZD1775 treated cell sample. Red color tones indicate higher gene set score.

e. Quantitative reverse transcription PCR (qRT-PCR) analysis of *BCL6* and *SOCS1* mRNA levels at 48 h AZD1775 treatment adjusted to the *GAPDH* mRNA, relative to the DMSO control is shown.

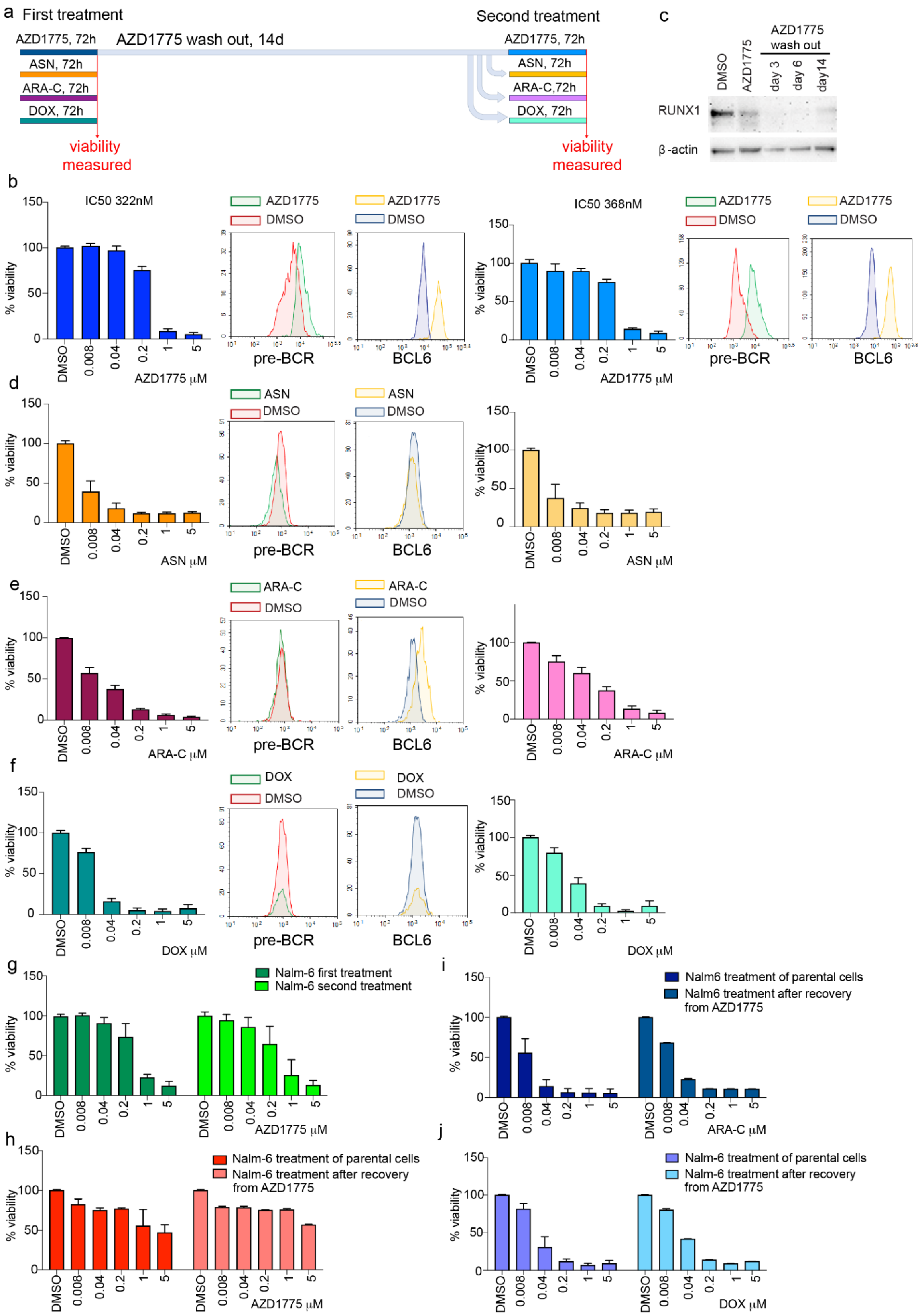

Fig. S7. related to Fig. 6

a. Schematic overview of the experimental setup for assessing drug sensitivity of the RS4;11 and Nalm-6 cell lines following 14 day recovery from the AZD1775 treatment.

b. AZD1775 drug response in RS4;11 parental cells (left) and recovered cells (right) as assessed by Alamar blue assay after 72 h treatment with increasing concentrations of AZD1775. Representative flow cytometry histograms of pre-BCR receptor and BCL6 protein after first and second treatment is shown after corresponding sensitivity plots.

c. RUNX1 protein levels in RS4;11 treated with AZD1775 72 h, followed by the drug washout for 3, 6 and 14 days.

d. Asparaginase (e) Cytarabine (f) Doxorubicin drug response in RS4;11 parental cells (left) and cells recovered after AZD1775 treatment (right) as assessed by Alamar blue assay after 72 h treatment. Representative flow cytometry histograms of pre-BCR receptor and BCL6 protein after first treatment is shown after the corresponding plots.

g. AZD1775, (h) asparaginase (i) Cytarabine (j) Doxorubicin drug response in Nalm-6 parental cells (left) and cells recovered after AZD1775 treatment (right) as assessed by Alamar blue assay after 72 h treatment.

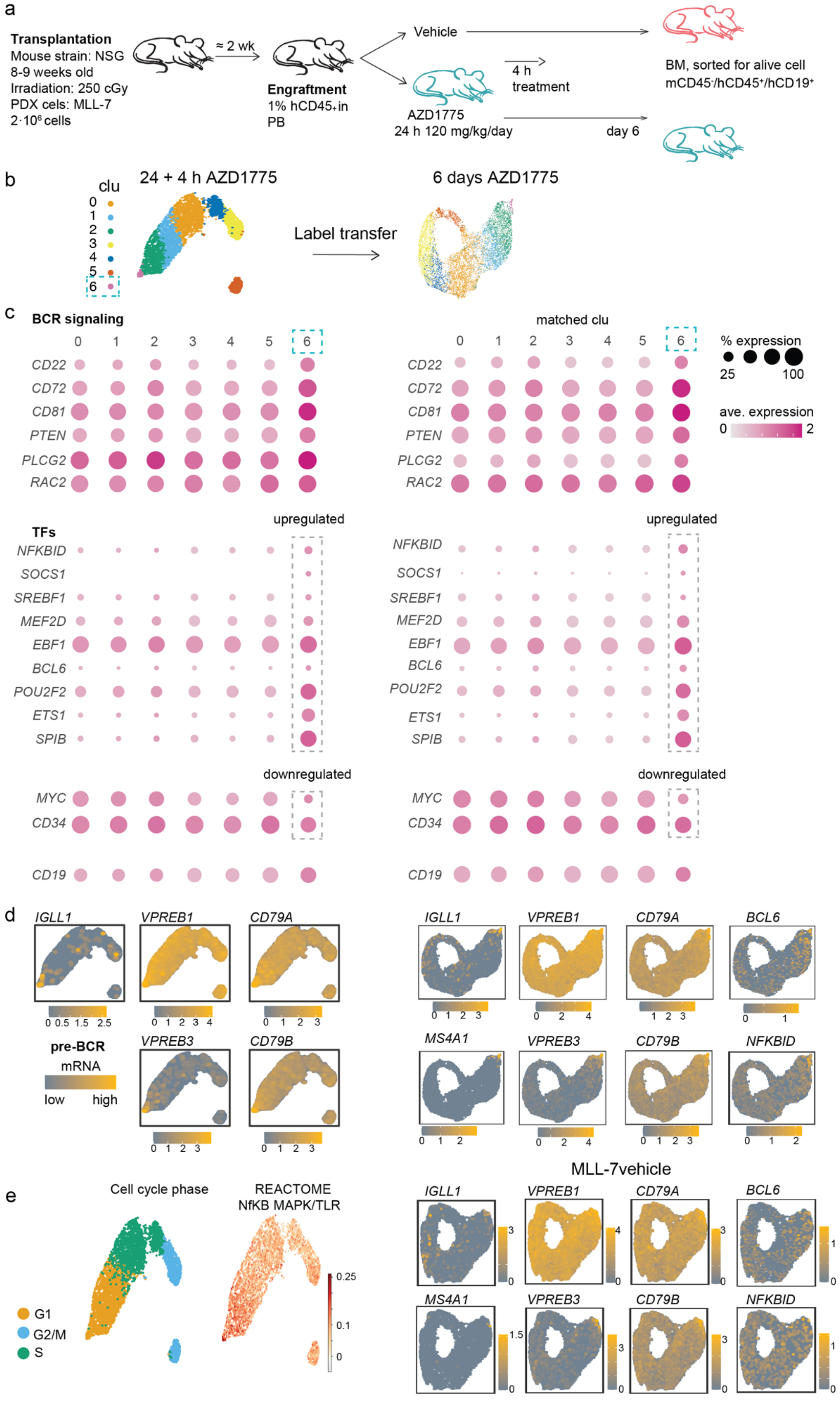

Fig. S8 related to Fig. 6.

a. Schematic representation of the experimental plan depicting the treatment arms and downstream analysis. After 28 h and two doses (24 h + 4 h) of 120 mg/kg AZD1775 animals were sacrificed and the cells were analysed by flow cytometry and hCD45+hCD19+ cells were sorted for scRNA-seq.

b. scRNA-seq transcriptome-based clustering and UMAP visualizations for primary KMT2A-r MLL-7 cells treated with AZD1775 (left: 24 h + 4 h, right:6 days). Colors correspond to clusters (left) or predicted matching cluster label at day 6 (right).

c. Dot plot heatmap showing expression levels of BCR-signaling pathway related genes and pre-B cell fate TF genes. The percentage of cells expressing each gene is indicated by dot size and average expression level by tones of red.

d. mRNA level for pre-BCR genes (top, brighter color tones correspond to higher level). Bottom right panel: vehicle control sample.

e. Assigned cell cycle status (left) and gene set score for NfKB MAPK/TLR pathway (right, red color tones indicate higher values).

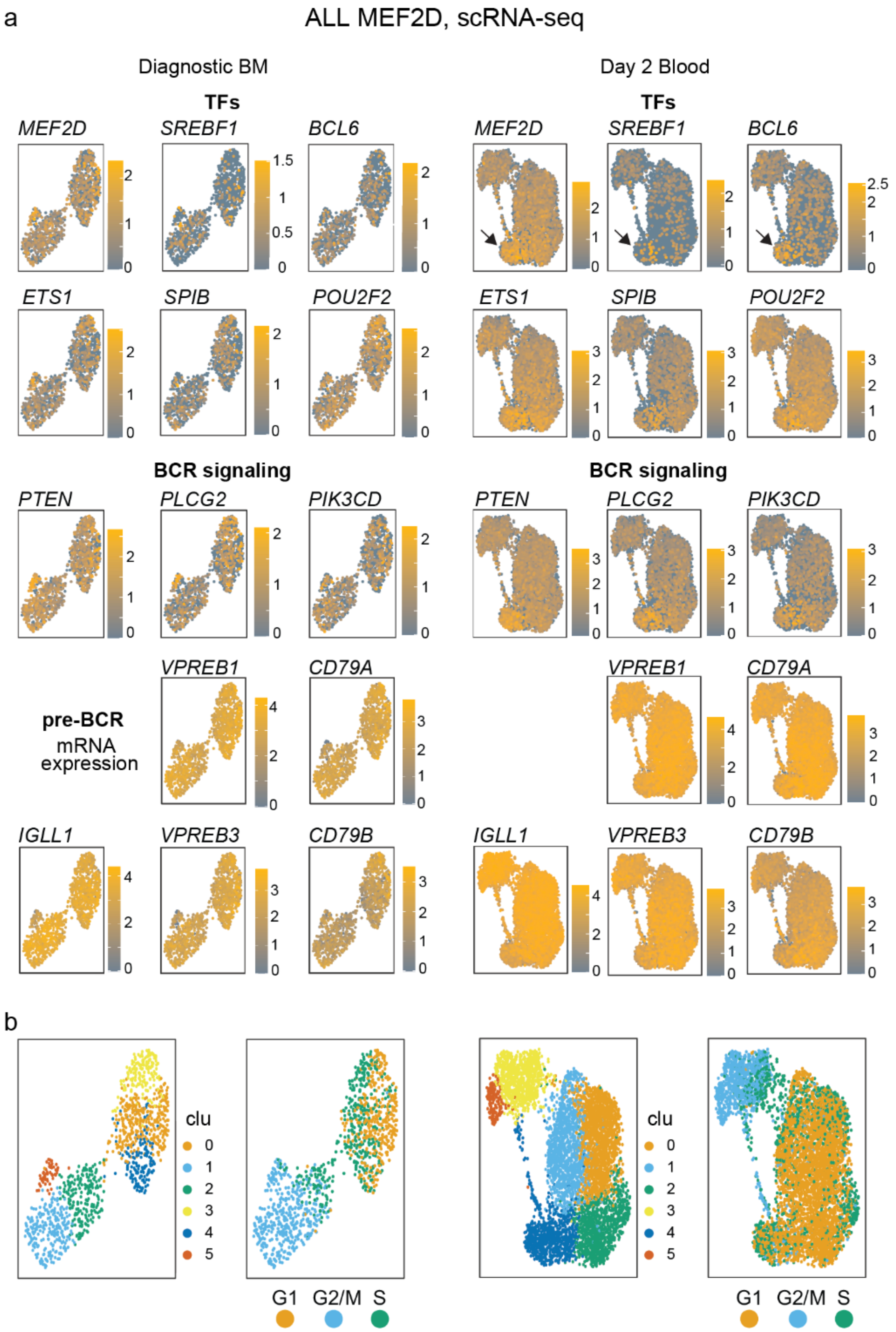

Fig. S9 related to Fig. 6.
Primary MEF2D-fusion ALL case treated with standard chemotherapy (left: diagnostic bone marrow, right: day 2 blood).
a. mRNA level for TFs, BCR signaling pathway and pre-BCR genes (brighter color tones correspond to higher level). Notice the different scales. High expression of TFs *MEF2D*, *SREBF1* and *BCL6* at day 2 (in cluster 4) is indicated by arrow.
b. Cluster assignment and cell cycle state annotated on UMAPs.

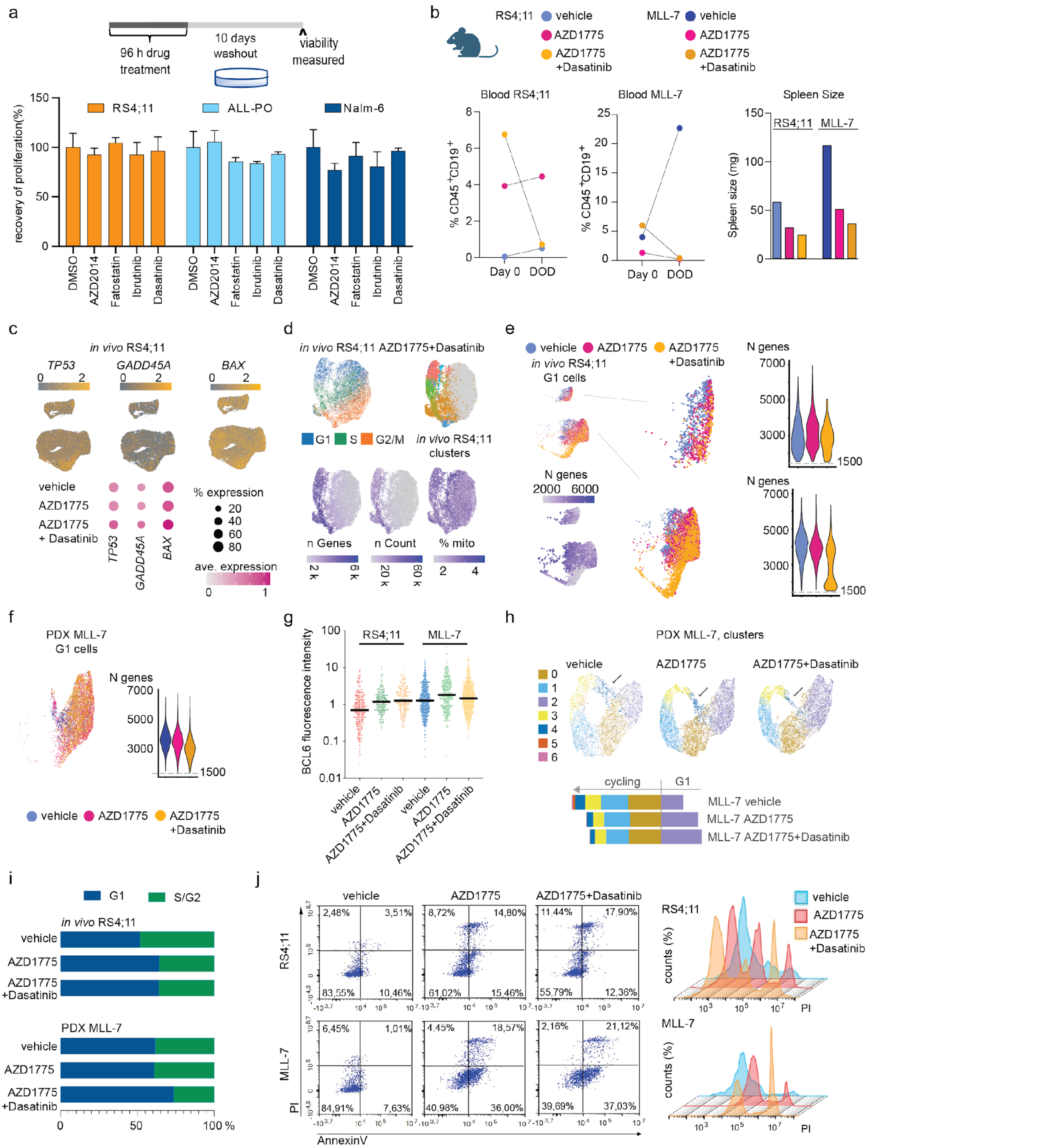

Fig. S10 related to Fig. 7.

a. Upper panel: overview of the experimental setup. Cells were treated for 4 days with indicated drugs and allowed to recover for 10 days without the drugs. Lower panel: recovery of cell proliferation following drug removal shown as in Fig. 7a.

b. Flow cytometric quantification of (left) human CD19+CD45+ cells in peripheral blood at day 0 and day 6 in RS4;11 and MLL-7 xenografts that were subsequently analyzed by scRNAseq; (right) spleen weight of RS4;11 and MLL-7 PDX mice at day 6.

c. Expression levels of *TP53*, *GADD45A* and *BAX* visualized on UMAPs and dotplots (RS4;11).

d. *in vivo* RS4;11 model scRNA-seq data from drug combination with less stringent filtering (% mito <5, n Genes > 500). Cell cycle state, clusters from fig 7h and quality metrics are shown.

e. and **f.** UMAP visualization of G1 cells by treatment group and quality metric violin plots (N genes: number of genes).

g.Quantification of BCL6 fluorescence intensity. Black bar indicates the median and dots correspond to individual cells.

h. Cluster proportion plots and UMAP visualizations by treatment group in MLL-7 PDX model. Cells exiting cell cycle are indicated.

i. Proportion plot of cell cycle distribution of RS4;11 and MLL-7 cells, quantified using DAPI staining as in (Akopyan et al., 2014)

j. Left panels: flow cytometric analysis of apoptosis in RS4;11 and MLL-7 xenograft cells by annexin V/PI staining. Right panels: 3D histogram overlay of PI signal.
