# Supplementary material for: Sequential drug treatment targeting cell cycle and cell fate regulatory programs blocks non-genetic cancer evolution in acute lymphoblastic leukemia
Source: Genome Biol. 2024 May 31;25:143. doi: 10.1186/s13059-024-03260-4 (PMC11143599; doi:10.1186/s13059-024-03260-4)
Supplement: Supplementary file 7 — Additional file 7. Contains original images of uncropped western blots. [file 13059_2024_3260_MOESM7_ESM.pdf]

Figure 1E

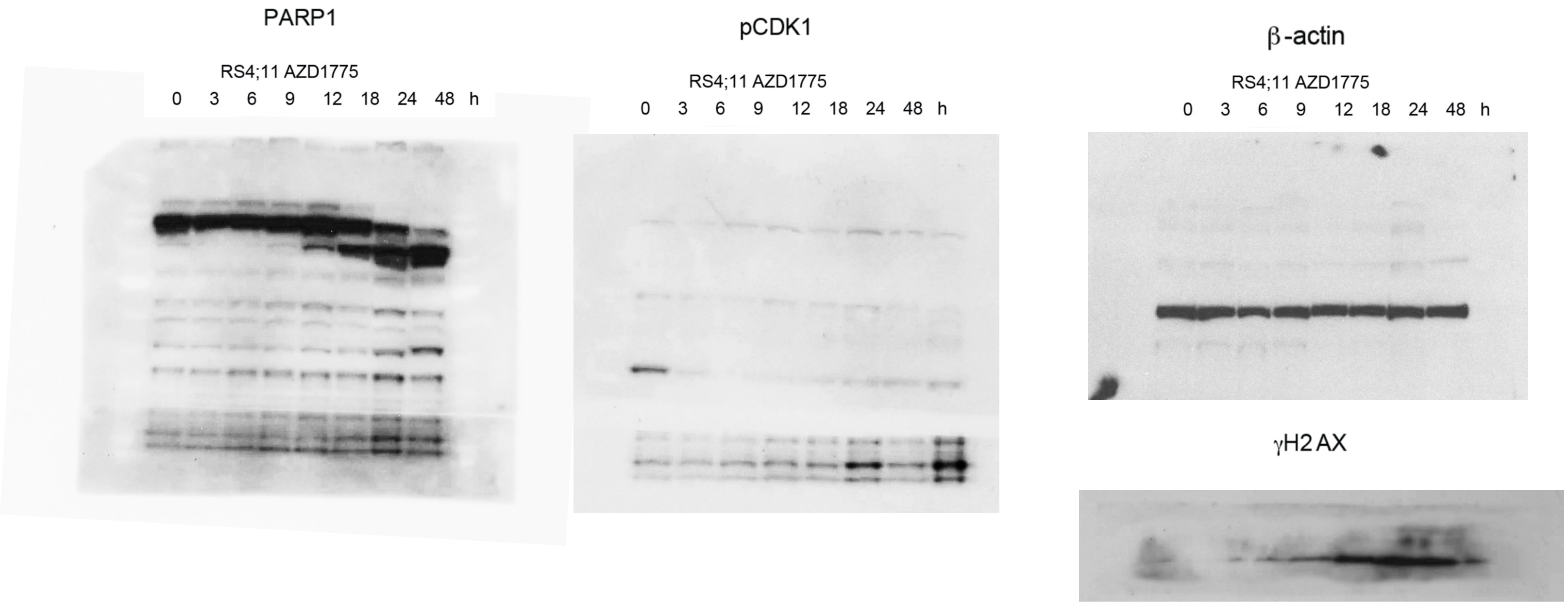
PARP1
RS4;11 AZD1775
0 3 6 9 12 18 24 48 h
pCDK1
RS4;11 AZD1775
0 3 6 9 12 18 24 48 h
β-actin
RS4;11 AZD1775
0 3 6 9 12 18 24 48 h
γH2 AX

Figure 5A

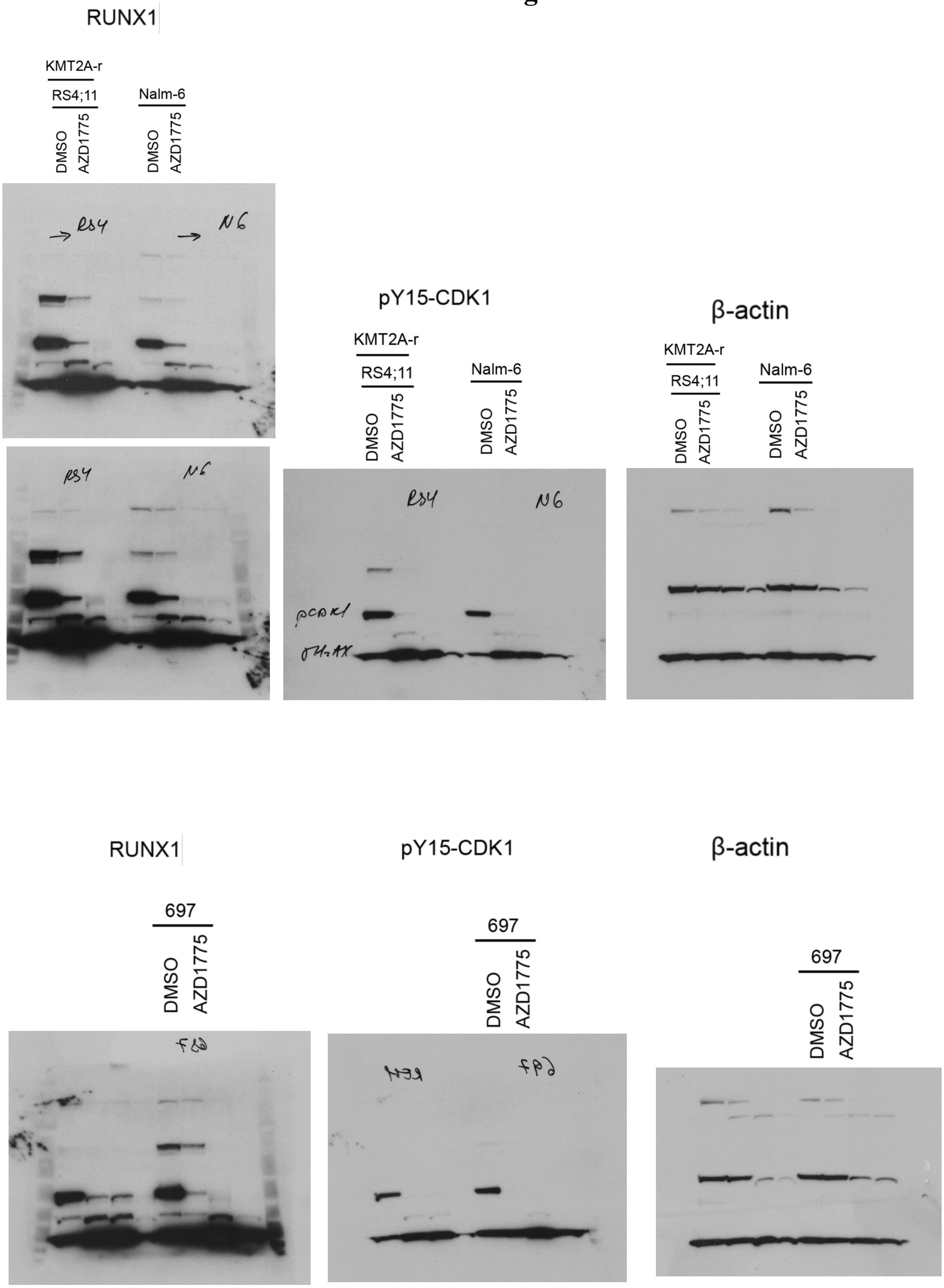
RUNX1
KMT2A-r
RS4;11
Nalm-6
DMSO
AZD1775
DMSO
AZD1775
pY15-CDK1
KMT2A-r
RS4;11
Nalm-6
DMSO
AZD1775
DMSO
AZD1775
β-actin
KMT2A-r
RS4;11
Nalm-6
DMSO
AZD1775
DMSO
AZD1775
RUNX1
697
DMSO
AZD1775
pY15-CDK1
697
DMSO
AZD1775
β-actin
697
DMSO
AZD1775

Figure 5B

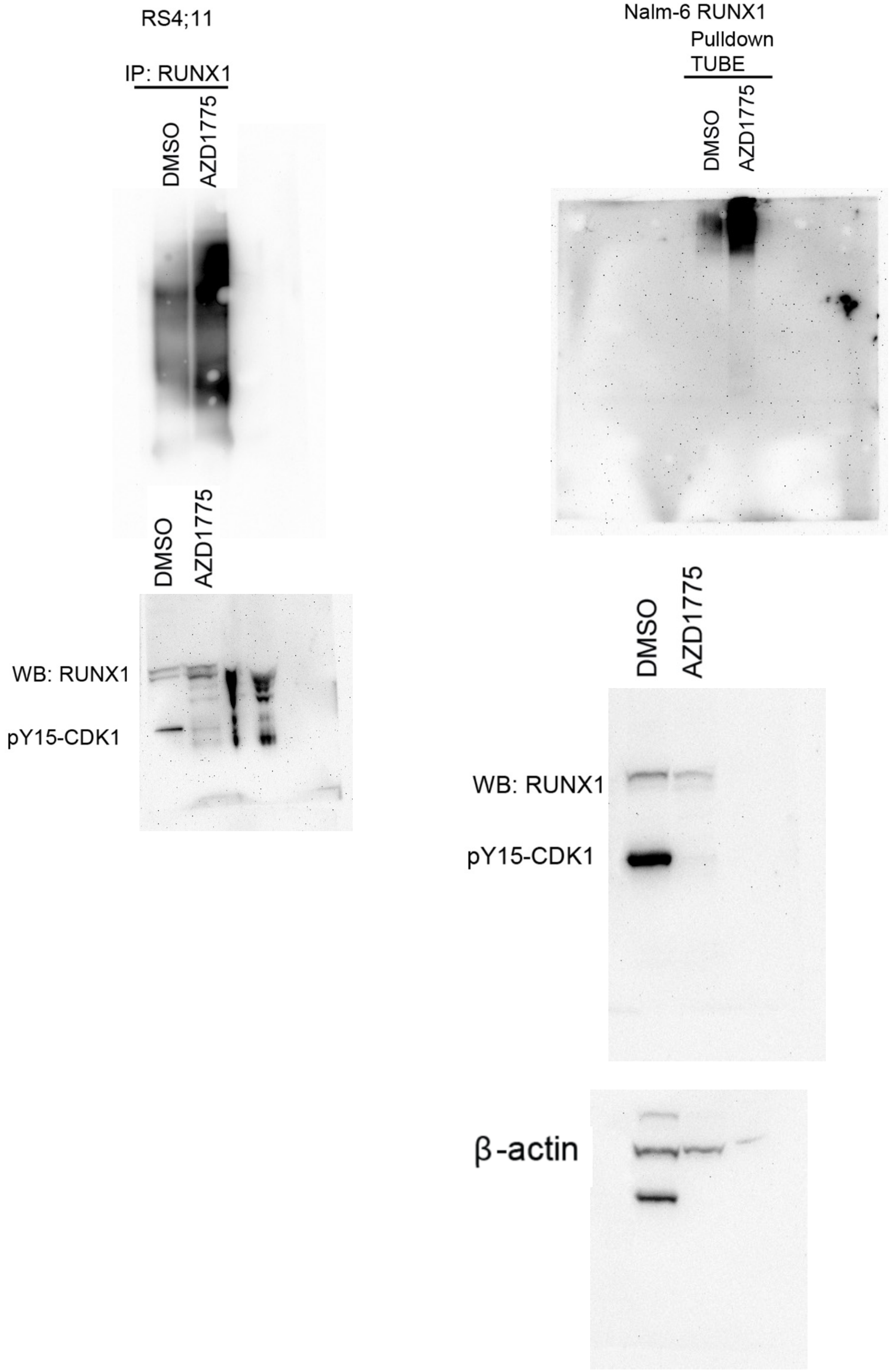
RS4;11
IP: RUNX1
DMSO
AZD1775
DMSO
AZD1775
WB: RUNX1
pY15-CDK1
Nalm-6 RUNX1
Pulldown
TUBE
DMSO
AZD1775
DMSO
AZD1775
WB: RUNX1
pY15-CDK1
β-actin

Figure 5C

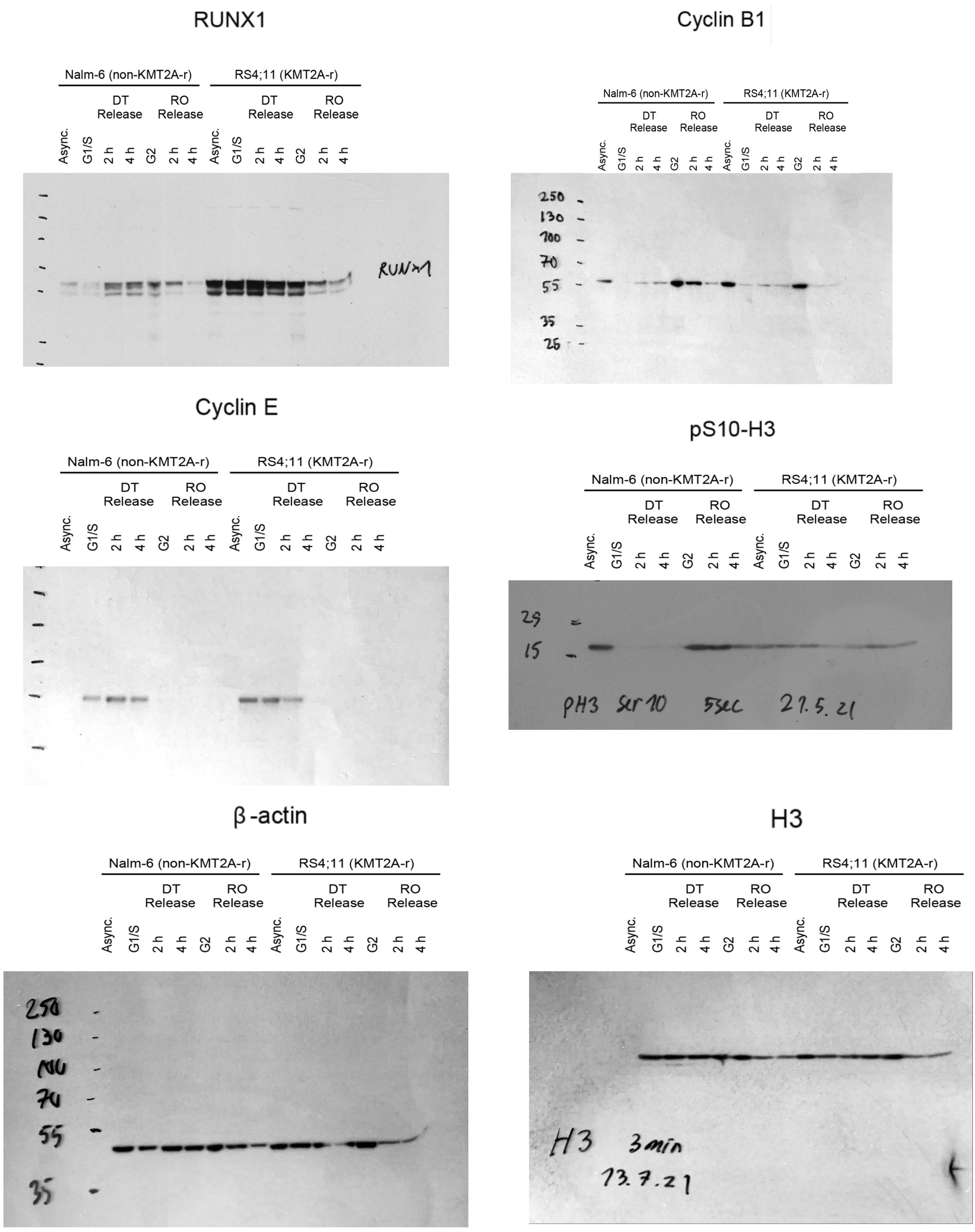
RUNX1
Nalm-6 (non-KMT2A-r)
RS4;11 (KMT2A-r)
DT Release
RO Release
Async.
G1/S
2 h
4 h
G2
RUNX1
Cyclin B1
250
130
100
70
55
35
25
Cyclin E
pS10-H3
25
15
PH3 Ser10 5sec 21.5.21
β-actin
250
130
100
70
55
35
H3
H3 3min
13.7.21

Figure 5D

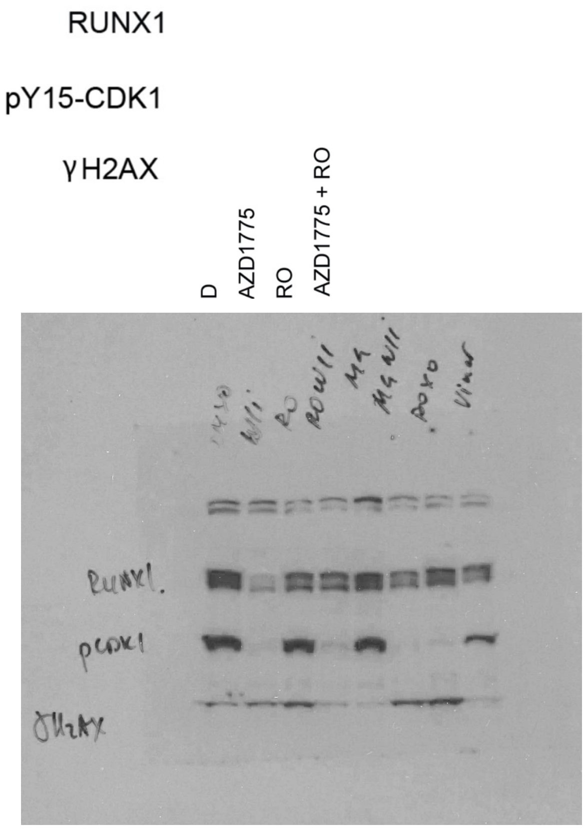
RUNX1
pY15-CDK1
γH2AX
D
AZD1775
RO
AZD1775 + RO

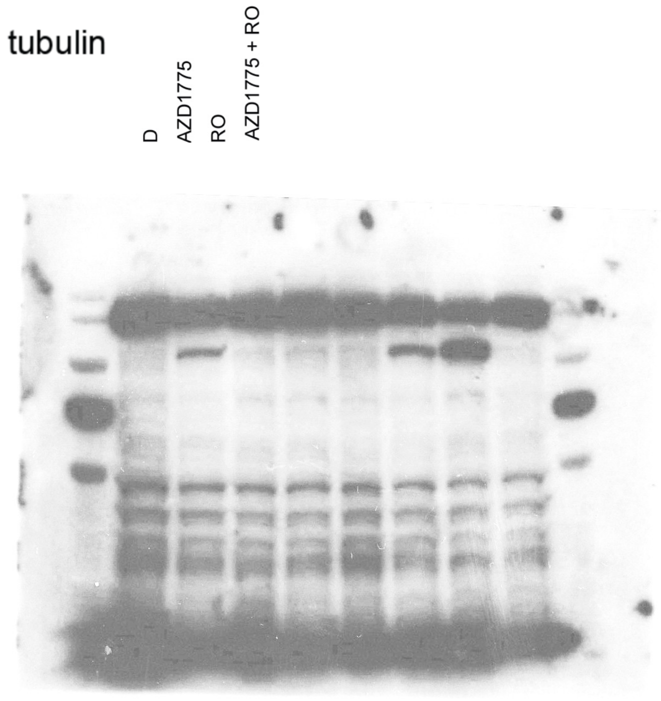
PARP
tubulin
D
AZD1775
RO
AZD1775 + RO

Fig. S3F

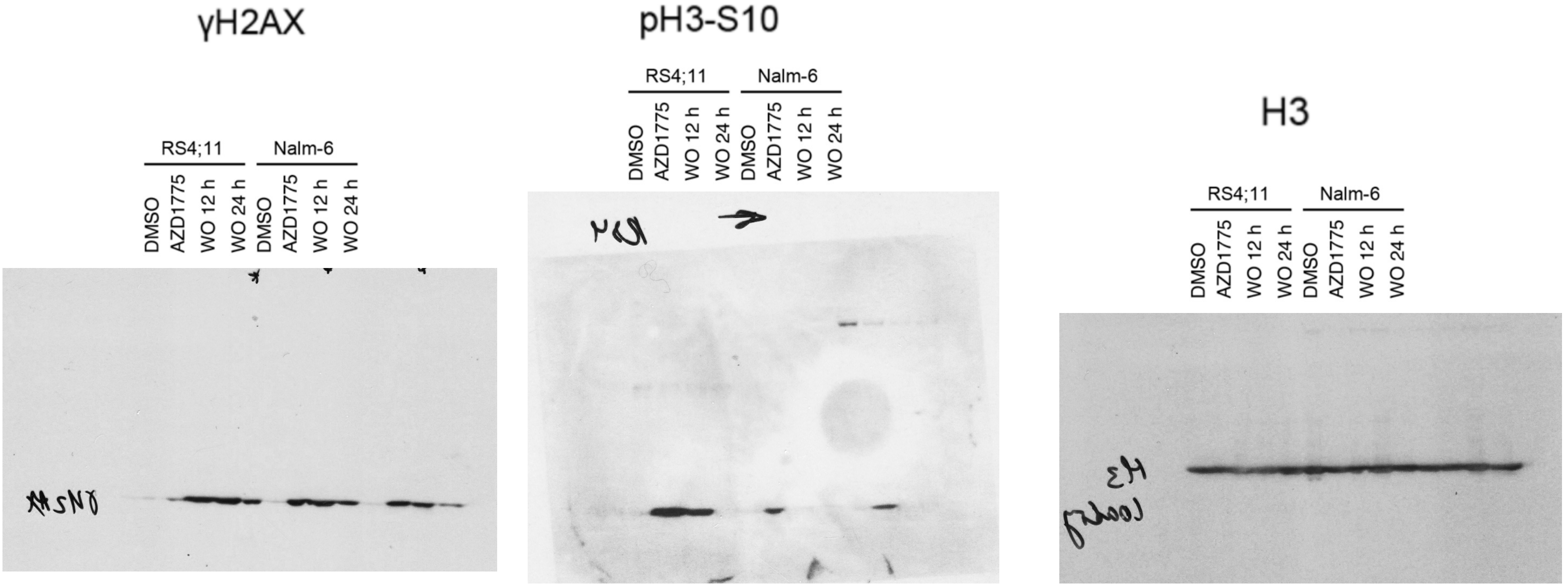

Fig. S5A

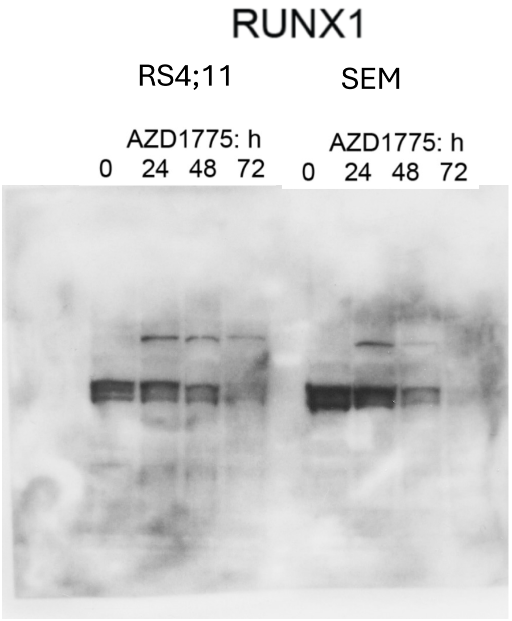

Fig. S5B

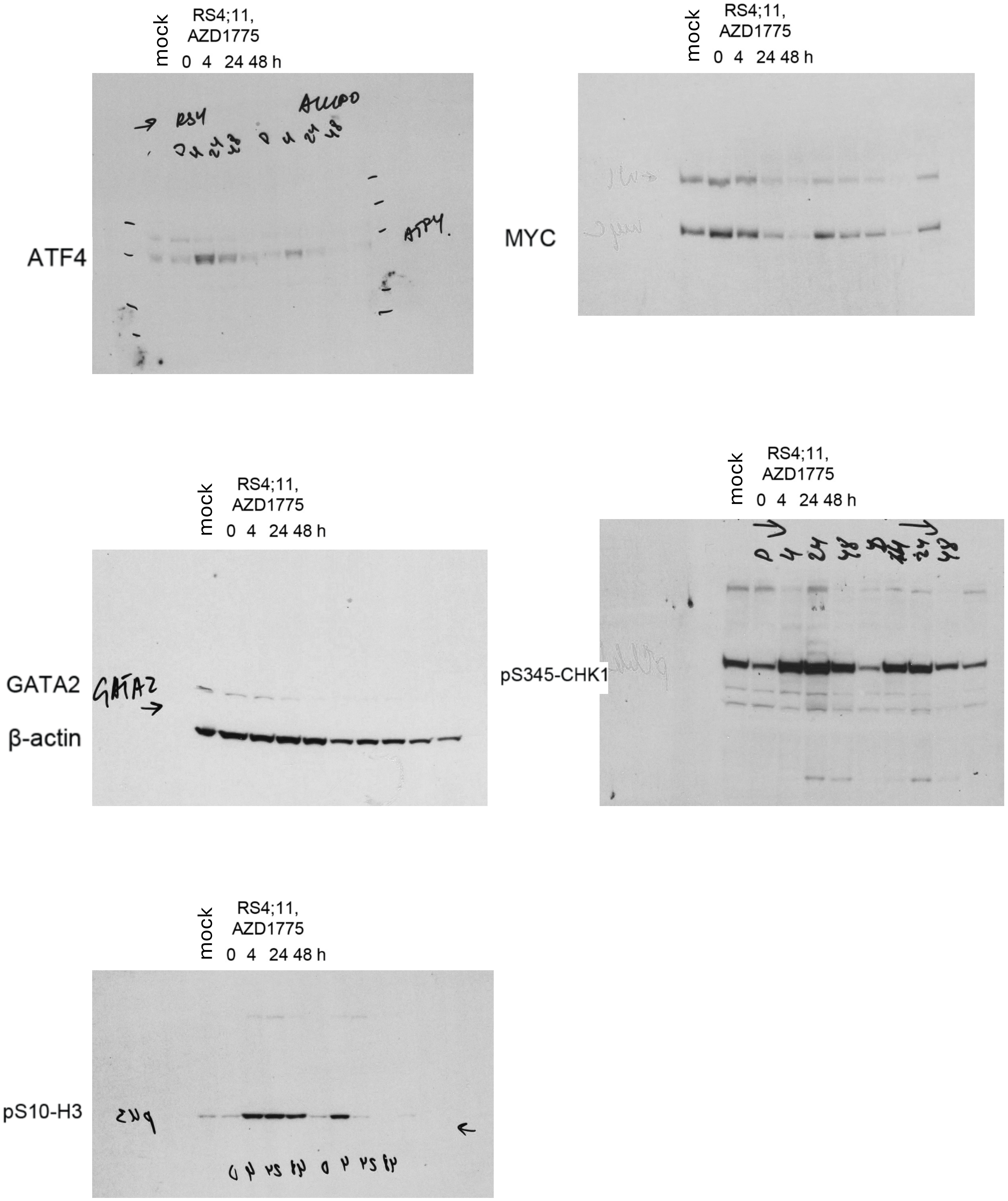
mock
RS4;11,
AZD1775
0 4 24 48 h
ATF4
mock
RS4;11,
AZD1775
0 4 24 48 h
MYC
mock
RS4;11,
AZD1775
0 4 24 48 h
GATA2
β-actin
mock
RS4;11,
AZD1775
0 4 24 48 h
pS345-CHK1
mock
RS4;11,
AZD1775
0 4 24 48 h
pS10-H3

Fig.S7C

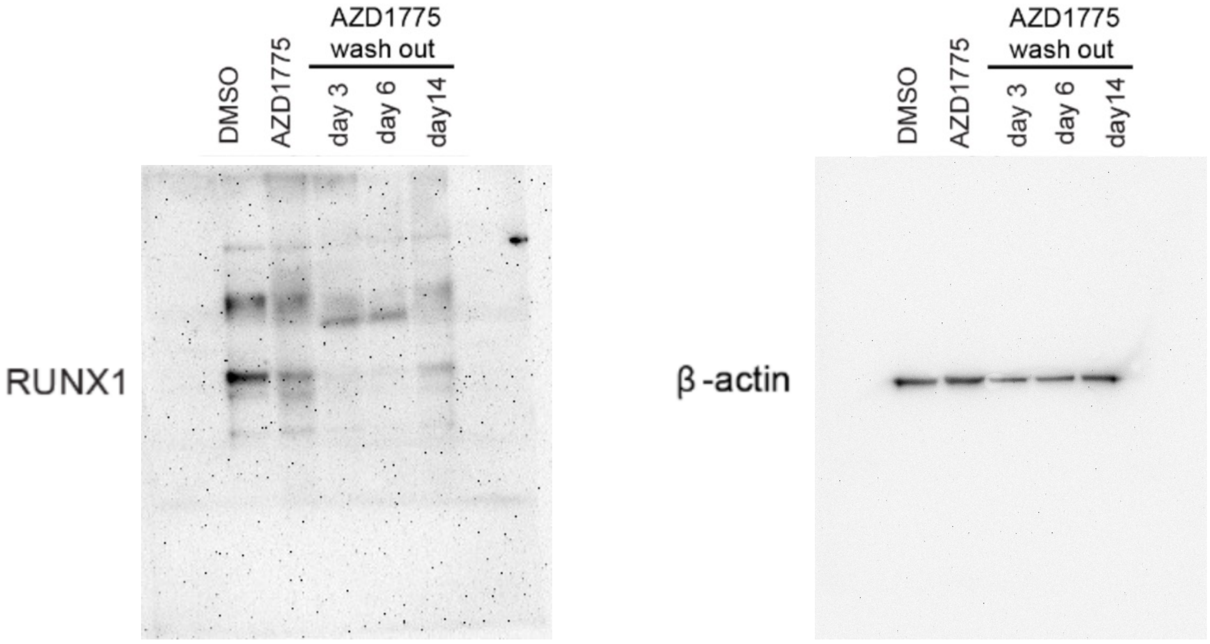

DMSO
AZD1775
AZD1775
wash out
day 3
day 6
day14
RUNX1
DMSO
AZD1775
AZD1775
wash out
day 3
day 6
day14
β-actin
