# Supplementary material for: Sequential drug treatment targeting cell cycle and cell fate regulatory programs blocks non-genetic cancer evolution in acute lymphoblastic leukemia
Source: Genome Biol. 2024 May 31;25:143. doi: 10.1186/s13059-024-03260-4 (PMC11143599; doi:10.1186/s13059-024-03260-4)
Supplement: Supplementary file 8 — Additional file 8. Review history. [file 13059_2024_3260_MOESM8_ESM.docx]

Review History

**First round of review**

**Reviewer 1**

**Are you able to assess all statistics in the manuscript, including the appropriateness of statistical tests used?** Yes, and I have assessed the statistics in my report.

**Comments to author:**

In this paper, Malyukova et al. tried to elucidate the consequences and mechanisms of WEE1 inhibitor treatment on acute lymphoid leukemias. Extensive multi-omic research have been conducted in this study. The RS4;11 cell line with KMT2A-r arrangement were first selected based on WEE1 expression. After treatment, an inhibition of proliferation recovery rather than acute response was found, thus authors tried to verify a drug-induced non-genetic state for the recovering cells. GRO-seq were used to characterize gene activity progression patterns at different time. By scRNA/ATAC analyses of AZD1775-treated/control cells at interested time points, authors found a G1/S checkpoint arrest pattern and characterized three cell fates in which fate III was recognized as drug tolerant. Subsequent analyses including chromatin accessibilities and TF activity patterns were performed, leading to the identification of core TFs and regulatory circuits for each cell fate. Finally, authors showed that a scheduled drug usage may work while concurrent drug combinations fail. This paper covered a wide range of aspects without counterfactual logic. However, several concerns must be addressed before it can be considered for publication.

major:

1. How to distinguish a drug-induced resistance state from a clonal evolution state of drug-resistant cell subpopulations? The authors suggest that drug-induced states can be characterized by altered expression profiles and changes in TF regulatory features, but these differences may also arise from different responses of specific subpopulations. As non-pre-existing patterns can still be subpopulation-related, a more detailed discussion is needed here.

2. The cell distribution in clusters in figure 2e could be a result of batch effect. Did the authors consider batch correction or evaluated its influence? Decisions should be made carefully as this will affect the label transfer process and further annotations.

3. In this paper, various clusterings marked by numbers are used, including cell states, chromatin states, cell fates, TF motif access patterns, in vivo treatment clusters... Considering the incorporation of different cell lines, time points, single-cell techniques etc., more different clusters have added great complexity to read and understand. A more clear structure & illustration is highly needed, maybe as a supplementary plot (Sankey diagram?). My suggestion is to set cell fate as the center.

4. Some statements in this study should have statistical support like p-values or correlation coefficients, e.g. "The tightly coordinated expression of MEF2D, BCL6 and SREBF1 ..."

5. The better outcome of scheduled drug usages can be explained by an alteration of intra-tumoral heterogeneity under a competing scheme. For example, a delayed usage of drug 2 may provide the opportunity for a high-sensitivity high-fitness subclone to out-compete other cells.

6. The proportion of the cells resembling the drug-resistant subpopulation is rather low at different time points (Figure 2g) and conditions (Figure 1a). What is the significance then? Will the proportion of these cells be higher after recovering from treatment? Authors should show these results.

minor:

1. Sufficient information should be provided in plot itself. e.g., figure 1a: what type of dimension reduction is used? t-sne? what type of expression values are shown? calculated by limma-voom? are these comparable? are there significances? figure 1b: what does M mean? figure 1f: should S/G2 be S/G2M according to the figure legend and main text? Also mind the typos: torelant -> tolerant cellls -> cells

2. The ub band in figure 5b isn't that obvious. While I believe the ubi-proteasome process on RUNX1 is real, authors may need to find stronger evidence or try to improve the experiment. Also, why use phospho-CDK1 Y15 to represent a WEE1 inhibition? There should be WEE1 antibodies.

3. Cluster 10 in Figure 3 is missing. It's recommended to use unified colors for Figure 6f and 6g.

4. Where does the four clusters in Figure s5c come from? Figure s3b have five clusters. It's hard to draw the conclusions about motif access at G1/G2 from this figure. More information should be provided for better understanding, not only in this one.

5. High/low color annotations are commonly seen in figures. Some of the annos (e.g., mRNA expression) should be replaced by actual values, or list them in a supplementary file.

**Reviewer 2**

**Are you able to assess all statistics in the manuscript, including the appropriateness of statistical tests used?** No, I do not feel adequately qualified to assess the statistics.

**Comments to author:**

The authors performed a single cell genomics analysis in acute lymphoblastic leukemia (ALL) cell lines and primary ALL cells coupled with biochemical assays to demonstrate that drug scheduling has a critical impact on the efficacy of targeted cancer drugs. Strikingly, it was demonstrated that the combined treatment of a Wee1 kinase inhibitor (AZD1775) with other targeted drugs (targeting BCR signaling, mTOR or fatty acid synthesis) were not able to prevent recovery of a treatment-persistent cell population, whereas in contrast sequential treatment were able to eradicate persistent cell population. The first part of the paper characterizes these "persister" cells, which evade Wee1 inhibition, in great detail and show a dramatic remodeling of gene regulatory networks. Here, it was shown that three main cell states were selected upon Wee1 kinase inhibitor treatment: extended mitotic arrest with a specific condensed chromatin state, activation of apoptosis and senescence pathways through p53 and higher chromatin access of NFkB, SREBF that regulating BCL6 and pre-BCR genes.

Overall, the authors have used state-of-the-art transcriptomics and bioinformatics methods that allow to dissect mRNA expression and gene regulatory networks at the single cell level. The applied methods are appropriate to the aims of the study and a large amount of high-quality data is presented. The results and methods are well described; few details are missing and should be added to the revised manuscript (see below). All experiments contain the necessary controls. The conclusions of the manuscript are supported by the presented data and appropriately discussed. The Materials and Methods section contains sufficient details to allow study replication. As detailed above, the study represents a significant advance in the field, as I am not aware of other studies that have demonstrated with such mechanistic depths how sequential treatment of two targeted inhibitors can be superior to their concomitant use. Therefore, the paper is of broad interest to others in the field and will be of outstanding interest to the broader cancer research community.

Minor points:

1. line 118/119: an approximate range of GC50 values of the dose-response curves in Fig. 1b should be added to the text.

2. line 141: control experiments with Nalm6 cells are mentioned, but no reference to where this data is shown, is provided. Please a reference to figure or SI.

3. line 386-190: I could not find the concentrations of the drugs that were used in Figure 7 c/d. These should be added to the figure legend and/or text.

**Reviewer 3**

**Are you able to assess all statistics in the manuscript, including the appropriateness of statistical tests used?** No, I do not feel adequately qualified to assess the statistics.

**Comments to author:**

In this manuscript Malyukova et al. characterize a non-genetic mechanism of cancer evolution in the context of KMT2A-rearreanged ALL upon cell cycle-targeting treatment. By analyzing the single-cell transcriptome and chromatin accessibility, the researchers observed that WEE1 inhibition led to diversification of cell states at critical decision points, resulting in the identification of a subpopulation of cells with active stress and pre-B cell transcriptional programs, potentially linked to drug tolerance.

The work is generally well-designed and written, and results were carefully analyzed, especially those regarding single-cell sequencing data. Overall this study provides relevant information related to the non-genetic evolution of leukemic cells and propose an innovative targeted therapy based on a sequential treatment strategy. These are two of the main points in terms of scientific relevance and originality in the manuscript which, if further validated, can be of general interest to the leukemia and cancer therapy communities.

However, there are some questions that need to be addressed in order to clarify the main conclusions.

Major points:

- As one of the strongest take-home messages from the study, authors state that "in this cell state diversification induced by WEE1 inhibition, a subpopulation transitioned to a cell state characterized by activation of transcription factors regulating pre-B cell fate, lipid metabolism and pre-BCR signaling which supported a drug tolerance".

First, the so-called drug-tolerant state derived in KMT2A-r cells upon WEE1 exposure is not sufficiently demonstrated. Authors should provide further experimental evidence of the sensitivity of the cell-fate-III subpopulation to either WEE1 inhibitor or even to other agents used in the clinics for ALL management. For example, following the scheme used for figures 1C and S1C, authors can expose those surviving cells recovered after WEE1 inhibitor wash-out to different drugs, both in a KMT2A-r and a non-KMT2A-r context.

Importantly, the data provide does not preclude the pre-existence of an infra-detected subpopulation of cells corresponding to the cell-fate-III profile. This reviewer acknowledges the difficulty in refuting this scenario, but either authors demonstrate robustly this point (i.e. finding a way to track cells co-expressing pre-BCR and BLC6 under basal conditions) or they should temper the affirmation of a transition towards a different cellular state.

- Authors should use a PDX-mouse model to test the efficacy of the sequential therapy strategy proposed in vivo.

- Molecular mechanisms underlying cell state diversification upon WEE1 inhibition should be further discussed. Other authors (i.e. Gupta et al., Science Advances 2022) have also reported acceleration in cellular diversification in response to stress or injury in a KMT2A-rearranged leukemia system. Despite the authors describe such effect upon WEE1 inhibition affecting RUNX1 protein levels, a more general mechanism supporting a limited capacity of KMT2A-r cells to cope with stress can be also considered. At least, authors should address these comments in the discussion section.

Minor:

- Figure 1 and related. Authors claim "Acute drug treatment induced apoptosis as measured by activation of caspase-3/7" but data is not shown.

- Recovery experiments: normalization of data should be further detailed. Which is the reference value for % of cell viability calculations?

- Any hints of how WEE1 expression is upregulated in KMT2A-r ALLs vs non-KMTA2-r cases?

- Differentiation vs stemness status of the surviving cells upon treatment is not evaluated.

**Authors Response**

**Point-by-point responses to the reviewers’ comments:**

*General response:*

*We were thrilled that all reviewers were positive on the manuscript as a whole. The insightful feedback we received has been instrumental in refining our manuscript. To address the points raised, we have conducted additional experiments and diligently incorporated the suggested additions and changes, which we agree have substantially improved the clarity and impact of our work. We extend our deepest thanks to the reviewers for their time and dedication to enhancing the quality of our manuscript.*

Reviewer #1:

In this paper, Malyukova et al. tried to elucidate the consequences and mechanisms of WEE1 inhibitor treatment on acute lymphoid leukemias. Extensive multi-omic research have been conducted in this study. The RS4;11 cell line with KMT2A-r arrangement were first selected based on WEE1 expression. After treatment, an inhibition of proliferation recovery rather than acute response was found, thus authors tried to verify a drug-induced non-genetic state for the recovering cells. GRO-seq were used to characterize gene activity progression patterns at different time. By scRNA/ATAC analyses of AZD1775-treated/control cells at interested time points, authors found a G1/S checkpoint arrest pattern and characterized three cell fates in which fate III was recognized as drug tolerant. Subsequent analyses including chromatin accessibilities and TF activity patterns were performed, leading to the identification of core TFs and regulatory circuits for each cell fate. Finally, authors showed that a scheduled drug usage may work while concurrent drug combinations fail. This paper covered a wide range of aspects without counterfactual logic. However, several concerns must be addressed before it can be considered for publication.

major:

1. How to distinguish a drug-induced resistance state from a clonal evolution state of drug-resistant cell subpopulations? The authors suggest that drug-induced states can be characterized by altered expression profiles and changes in TF regulatory features, but these differences may also arise from different responses of specific subpopulations. As non-pre-existing patterns can still be subpopulation-related, a more detailed discussion is needed here.

*Response: We appreciate the valuable input from the reviewer and acknowledge the significance of addressing this intricate point, which was also brought up by reviewer #3 (as detailed below). We share the reviewer's viewpoint and recognize the difficulty of conclusively excluding the existence of a pre-existing minor subpopulation that may represent cell state III (or a non-pre-existing pattern that is subpopulation-related) in the treated samples.*

*In response to this concern, further characterization and additional experiments have been conducted and results discussed to better elucidate the distinction between the emergence of a drug-tolerant subpopulation (not detected in the untreated cells) as compared to clonal expansion of a pre-existing drug-resistant subpopulation.*

*In brief, while our data do not definitively rule out the possibility that the emergence of new subpopulations (drug-induced) arise from non-pre-existing patterns (undetectable before treatment), they do suggest that these subpopulations do not originate from a pre-existing subpopulation already resistant to the drug before the treatment commenced as outlined below.*

*As shown in New Fig S7a, we conducted additional experiments by subjecting RS4;11 cells that recovered from AZD1775 treatment to a second round of AZD1775 exposure (along with other chemotherapeutic drugs, see below, as recommended by reviewer #3). The cells that recovered post-treatment retained their sensitivity to AZD1775 (Fig S7a,b,c), and consistent with a dynamic and reversible response, the increased levels of pre-BCR and BCL6 returned to baseline upon removal of AZD1775 (new data added from day 6 and day 14 to Fig 6g,h, Fig S7a,b). Thus, the response to AZD1775 is reversible, and the cells revert back to their original proliferative capacity following withdrawal of the drug and start to re-express RUNX1 at day 14 (Fig. S7c). These findings further support the concept of a drug-induced transition of a subpopulation of cells that survive, rather than selection and expansion of a pre-existing resistant cell subpopulation during the recovery phase.*

*Additionally, we exposed KMT2A-r and non-KMT2A-r cells recovering from AZD1775 to several different drugs used in ALL treatment protocols (L-asparaginase, cytarabine, doxorubicin). As demonstrated in New Fig. S7d-f, cells that recovered post-AZD1775 treatment retained their sensitivity to standard chemotherapeutics, without activating pre-BCR signaling in KMT2A-r cells. This underscores the unique phenotypic shift induced by AZD1775, indicating that adopting a more pre-B-like cell state corresponds to a specific response to WEE1 inhibition. Altogether, as the cell state transition distinguished by distinct gene expression profiles (not observed in the DMSO-treated cells) is already evident at the 10 hour time point (Fig 2g), this indicates - although does not conclusively prove - that the subpopulation of drug-tolerant cells do not originate from a pre-existing subpopulation that uniformly expands in culture, as such a process would typically lead to the establishment of a stable and irreversible drug-resistant population.*

*We have carefully revised the Results and Discussion to present alternative scenarios and their agreement with data obtained.*

2. The cell distribution in clusters in figure 2e could be a result of batch effect. Did the authors consider batch correction or evaluated its influence? Decisions should be made carefully as this will affect the label transfer process and further annotations.

*Response: We have processed the RS4;11 cell line samples at the same time from the DMSO and AZD1775 treated cell cultures, representing the same parental line. Cells were loaded to the 10x Genomics Chromium controller and processed into libraries in parallel. Also, during data analysis, samples were first merged together and filtered in the same manner. Thus, we are confident that the clusters shown do not reflect technical handling batch effects. We have clarified this in the Methods section.*

3. In this paper, various clusterings marked by numbers are used, including cell states, chromatin states, cell fates, TF motif access patterns, in vivo treatment clusters... Considering the incorporation of different cell lines, time points, single-cell techniques etc., more different clusters have added great complexity to read and understand. A more clear structure & illustration is highly needed, maybe as a supplementary plot (Sankey diagram?). My suggestion is to set cell fate as the center.

*Response: We appreciate the reviewer's comments, and we sincerely apologize for any inconvenience caused by the difficulty in interpreting these figures. We agree that it is crucial to visualize the data in a reader-friendly way; that is what we have been endeavoring to accomplish based on color-coding. The RS4;11 scRNA-seq data collected at 24 h serves as the reference for clustering that we refer to as cell states (RNA) and chromatin states (ATAC). These colors are used throughout the paper. The scATAC-seq chromatin state correspondence to RNA cell states was evaluated based on 10 h multiome data, and matched cell state indicated by a similar color tone.*

*We produced a Sankey diagram that compares the correspondence of RNA and chromatin cluster labels at 10 h to better illustrate how we arrived at the cluster coloring scheme denoting specific cell fates. The labels of these cells for each modality are assigned based on the 24 h samples. The 10 h scMULTIOME cells (DMSO and AZD1775) are shown in the middle as a Sankey diagram that illustrates the 1:1 matching of RNA and ATAC profile that is directly available in the data (same cell barcodes). The correspondence between the cells is indicated by the connecting lines. (Rare categories of labels assigned to < 1.5% of cells analysed are omitted from the plot for clarity, as explained in the respective Methods section). We have added Sankey diagrams to Fig. S2a to support Fig.2 and interpretation of further results presented in Figs. 3-5.*

*Cell fates are referred to with roman numerals from I, II, and III, and correspondence of RNA and ATAC labels assigned to them are highlighted in the Sankey diagram added to Fig. S2a. The additional scRNA-seq data from in vivo profiles is referred to simply by cluster numbers and the sample names are included to each figure panel for clarity.*

4. Some statements in this study should have statistical support like p-values or correlation coefficients, e.g. "The tightly coordinated expression of MEF2D, BCL6 and SREBF1 ..."

*Response: We thank the referee for this suggestion. We included p-values to support statistical analysis results throughout the manuscript.*

*Related to the statement in quotes, we tested correlation statistical analysis of the MEF2D day 2 blood sample. To calculate pairwise gene correlations for genes of interest, including transcription factors (TFs) and BCR signaling genes, as illustrated in the original manuscript Figure 7b (now presented in Fig. S9), we initially performed the analysis using the scran tool (as detailed in https://rdrr.io/bioc/scran/man/correlatePairs.html). Unfortunately, the pair-wise correlation results across genes (in cluster 4 with highest expression of the TFs, or across all cells) revealed an abundance of drop-out values (i.e., 0 values) and correlation coefficients (rho) centered around zero.*

*To address this challenge, we mitigated the detection problem by computing correlations at cluster level based on the percentage of cells expressing a given gene. The correlation between the TFs MEF2D and BCL6 was 0.80 (Pearson rho, p-value 0.056) and between the TFs SREBF1 and BCL6 was 0.9 (Pearson rho, p-value 0.0157) in the MEF2-ALL sample that agrees with their previously characterized regulatory interactions. We updated Results text accordingly. However, since comparison of clusters does not ideally capture “coordinated” expression within cluster (technically difficult to assess, as shown above), we decided to emphasize the matching upregulation in specific clusters in the different in vivo samples analyzed (Fig. S8, S9) and revised the Results text accordingly.*

5. The better outcome of scheduled drug usages can be explained by an alteration of intra-tumoral heterogeneity under a competing scheme. For example, a delayed usage of drug 2 may provide the opportunity for a high-sensitivity high-fitness subclone to out-compete other cells

*Response: To address this thoughtful comment, we have tried to clarify this better in response to Q1. Our data do not definitively rule out the competing scheme. However, the drug re-treatment at day 14 did not indicate enrichment of more resistant cells that would support a putative high-fitness subclone that out-competes other cells. Rather we observed slow return of the original preBCR- state and TF activities, indicative of cell state transitions between metastable states. We have revised the Results and Discussion related to alternative scenarios to address this comment.*

6. The proportion of the cells resembling the drug-resistant subpopulation is rather low at different time points (Fig 2g) and conditions (Fig 1a). What is the significance then? Will the proportion of these cells be higher after recovering from treatment? Authors should show these results.

*Response: We appreciate the reviewer's observation regarding the relatively low proportion of cells resembling the drug-resistant subpopulation. We would like to clarify that the data shown in Figure 2 corresponds to the clustering of cells based on RNA expression and chromatin access (Figure 1a is not related to the comment, and shows HEMAP transcriptomic data across hematologic malignancies). As the reviewer correctly points out, Cell fate 3 is not representing the major population at these early time points that were profiled by single cell analysis (Fig 2).*

*As mentioned above in context of Q1, we have conducted further analyses to address the reviewer’s concern, and as shown in New Fig.6g, FigS7b, a substantial fraction of cells (assessed at the protein-level by flow cytometry) is positive for pre-BCR and BCL6 at 72 h treatment. We now provide additional data, measuring the levels of pre-BCR/BCL6-positive cells during recovery. Importantly, these results reveal that during the recovery period (day 6 and day 14), cells transition back to a pre-BCR/BCL6-negative state (Fig6f,g). This evolution supports the transient nature of the drug-tolerant state and its reversibility upon cessation of treatment.*

*We hope that the inclusion of these data in our manuscript, which aimed to address the reviewer's question regarding the (initially) observed low proportions of drug-tolerant cells, now clarifies this point and further highlights the dynamic changes in cell states that occur during recovery after treatment.*

Reviewer #1: minor:

1. Sufficient information should be provided in plot itself. e.g., figure 1a: what type of dimension reduction is used? t-sne? what type of expression values are shown? calculated by limma-voom? are these comparable? are there significances?

*Response: We have revised the figure and legend accordingly. Figure 1 shows a tSNE plot and the expression values shown are normalized microarray probe signal values. Statistical significance is denoted in the revised figure legend.*

2. The ub band in figure 5b isn't that obvious. While I believe the ubi-proteasome process on RUNX1 is real, authors may need to find stronger evidence or try to improve the experiment. Also, why use phospho-CDK1 Y15 to represent a WEE1 inhibition? There should be WEE1 antibodies.

*Response: We thank the reviewer for this comment and now provide additional data providing clearer evidence for RUNX1 polyubiquitination in RS4;11 and Nalm-6 cells. We repeated the ubiquitination assays in both RS4;11 and Nalm-6 cells using two different strategies; immunoprecipitation of RUNX1 followed by immunoblotting for ubiquitin in RS4;11 cells, and pulldown of polyubiquitinated species and immunoblotting for RUNX1 (as described in detail in the method section). The new results are shown in New Fig 5b, clearly demonstrating RUNX1 polyubiquitination in response to AZD1775 treatment.*

*The level of pY15-CDK1 can serve as a biomarker for the effectiveness of WEE1 inhibition and pY15-CDK1 antibodies are frequently used in experiments studying the effects of AZD1775. We have included the appropriate reference in the revised manuscript (Takebe et al, Clin Cancer Res (2021) 27 (14): 3834–3844 https://doi.org/10.1158/1078-0432.CCR-21-0329).*

3. Cluster 10 in Figure 3 is missing. It's recommended to use unified colors for Figure 6f and 6g.

*Response: The cluster labels were assigned based on 24 h sample. No cells were matched to this cluster in the 10 h RS4;11 sample. Fig. 6 has been revised with new plots.*

4. Where does the four clusters in Figure s5c come from? Figure s3b have five clusters. It's hard to draw the conclusions about motif access at G1/G2 from this figure. More information should be provided for better understanding, not only in this one.

*Response: We apologize. To clarify, we have visualized in the figures that follow Fig. 3 and Fig. S3 only clusters with comparable QC metrics, excluding cells with condensed chromatin state from the heatmap (corresponding to cluster 5 in DMSO-treated cells, refer to Fig. S3d). We have clarified this in the respective figure legends and Methods text. The results remain consistent even when displaying all chromatin states in the same heatmap. For example, chromatin state 5 from DMSO cells (shown in Fig. S5c) exhibits consistently low or negligible motif activity for the TF motifs analysed.*

*To aid the reader in their understanding of cell cycle status, we have added a Sankey plot to Fig. S2a, making it easier to follow the matching between cell states and cell cycle phases. From the Sankey plot panel, it can be seen that cell state 1 (G1 cells) match with chromatin state 1 (DMSO sample) with highest RUNX1 signal visible in the heatmap. The other cells in this cluster (chromatin state 1 DMSO cells) are matched by the Sankey diagram to cell states 2 and 3 that correspond to early and late S-phase.*

5. High/low color annotations are commonly seen in figures. Some of the annos (e.g., mRNA expression) should be replaced by actual values, or list them in a supplementary file.

*Response: We have modified the figures according to the reviewer’s suggestion.*

*Once again, we thank this Reviewer for their insightful and thoughtful suggestions to improve our manuscript.*

Reviewer #2: The authors performed a single cell genomics analysis in acute lymphoblastic leukemia (ALL) cell lines and primary ALL cells coupled with biochemical assays to demonstrate that drug scheduling has a critical impact on the efficacy of targeted cancer drugs. Strikingly, it was demonstrated that the combined treatment of a Wee1 kinase inhibitor (AZD1775) with other targeted drugs (targeting BCR signaling, mTOR or fatty acid synthesis) were not able to prevent recovery of a treatment-persistent cell population, whereas in contrast sequential treatment were able to eradicate persistent cell population. The first part of the paper characterizes these "persister" cells, which evade Wee1 inhibition, in great detail and show a dramatic remodeling of gene regulatory networks. Here, it was shown that three main cell states were selected upon Wee1 kinase inhibitor treatment: extended mitotic arrest with a specific condensed chromatin state, activation of apoptosis and senescence pathways through p53 and higher chromatin access of NFkB, SREBF that regulating BCL6 and pre-BCR genes.

Overall, the authors have used state-of-the-art transcriptomics and bioinformatics methods that allow to dissect mRNA expression and gene regulatory networks at the single cell level. The applied methods are appropriate to the aims of the study and a large amount of high-quality data is presented. The results and methods are well described; few details are missing and should be added to the revised manuscript (see below). All experiments contain the necessary controls. The conclusions of the manuscript are supported by the presented data and appropriately discussed. The Materials and Methods section contains sufficient details to allow study replication. As detailed above, the study represents a significant advance in the field, as I am not aware of other studies that have demonstrated with such mechanistic depths how sequential treatment of two targeted inhibitors can be superior to their concomitant use. Therefore, the paper is of broad interest to others in the field and will be of outstanding interest to the broader cancer research community.

*Response: We thank the referee for these positive remarks on our manuscript.*

Minor points:

1. line 118/119: an approximate range of GC50 values of the dose-response curves in Fig. 1b should be added to the text.

*Response: GI50 values are now added to Supplementary Information (New Table S1).*

2. line 141: control experiments with Nalm6 cells are mentioned, but no reference to where this data is shown, is provided. Please a reference to figure or SI

*Response: We have included the Nalm-6 cell control results in both the main figures and the Supplementary Information. Specifically, the relevant figures where Nalm-6 results is referenced are as follows: Fig 5a, b, c; Fig 7a,b; Fig S1b,c,d; Fig S3e, f; Fig S4a; Fig S7 g-j; and Fig S10a*

3. line 386-190: I could not find the concentrations of the drugs that were used in Figure 7 c/d. These should be added to the figure legend and/or text.

*Response: The concentrations of the drugs are now added to the respective legends*

Reviewer #3:

In this manuscript Malyukova et al. characterize a non-genetic mechanism of cancer evolution in the context of KMT2A-rearreanged ALL upon cell cycle-targeting treatment. By analyzing the single-cell transcriptome and chromatin accessibility, the researchers observed that WEE1 inhibition led to diversification of cell states at critical decision points, resulting in the identification of a subpopulation of cells with active stress and pre-B cell transcriptional programs, potentially linked to drug tolerance.

The work is generally well-designed and written, and results were carefully analyzed, especially those regarding single-cell sequencing data. Overall this study provides relevant information related to the non-genetic evolution of leukemic cells and propose an innovative targeted therapy based on a sequential treatment strategy. These are two of the main points in terms of scientific relevance and originality in the manuscript which, if further validated, can be of general interest to the leukemia and cancer therapy communities. However, there are some questions that need to be addressed in order to clarify the main conclusions.

Major points:

1)- As one of the strongest take-home messages from the study, authors state that "in this cell state diversification induced by WEE1 inhibition, a subpopulation transitioned to a cell state characterized by activation of transcription factors regulating pre-B cell fate, lipid metabolism and pre-BCR signaling which supported a drug tolerance".

First, the so-called drug-tolerant state derived in KMT2A-r cells upon WEE1 exposure is not sufficiently demonstrated. Authors should provide further experimental evidence of the sensitivity of the cell-fate-III subpopulation to either WEE1 inhibitor or even to other agents used in the clinics for ALL management. For example, following the scheme used for figures 1C and S1C, authors can expose those surviving cells recovered after WEE1 inhibitor wash-out to different drugs, both in a KMT2A-r and a non-KMT2A-r context.

Importantly, the data provide does not preclude the pre-existence of an infra-detected subpopulation of cells corresponding to the cell-fate-III profile. This reviewer acknowledges the difficulty in refuting this scenario, but either authors demonstrate robustly this point (i.e. finding a way to track cells co-expressing pre-BCR and BLC6 under basal conditions) or they should temper the affirmation of a transition towards a different cellular state

*Response: We appreciate the thoughtful and constructive feedback by the reviewer and for recognizing that our study provides relevant information related to the non-genetic evolution of leukemic cells. We have addressed the specific questions with either additional experiments or clarifications, as outlined below.*

*First, in response to the questions related to the sensitivity of the treatment-induced drug-tolerant cell subpopulation (cell fate III) and its potential pre-existence in unchallenged cells (also raised by reviewer #1), we have explored this further.*

*We conducted additional experiments utilizing the suggested drug-recovery setup as detailed in new Fig S7a. Cells after 14 days recovery from WEE1 inhibitor treatment were re-challenged with AZD1775 and various other drugs (doxorubicin, cytarabine, L-asparaginase) typically used in ALL therapy. The results presented in new Fig S7 demonstrate that the KMT2A-r RS4;11 cells, post-recovery, maintain their sensitivity to AZD1775, closely resembling the response of the original, untreated parent cells (Fig S7b). Similarly, cells recovering from AZD1775 exhibited consistent sensitivity to the other conventional drugs (Fig 7d-f). This finding implies that the cell population that survives is able to revert to its original state after the WEE1 inhibitor is washed out. Corroborating this, levels of pre-BCR and BCL6, marking the drug-tolerant population, and RUNX1 (new data added to Fig. S7) marking the initial gene regulatory state, slowly (within 6-14 days) normalized to baseline following the removal of the drug (as indicated in new Fig 6f,g, Fig S7b). This kinetics is distinct from an acute stress response that would be expected to return to the basal state more rapidly, as observed for ATF4 levels (Fig. S5b).*

*Compared to the data we present in the original manuscript, our flow cytometric analysis of vehicle treated cells indicated that 0.35% co-express pre-BCR and BCL6, while at 72 h nearly all cells (88.96%) exhibit the pre-BCR+/BCL6+ protein expression pattern upon WEE1 inhibition at IC50 concentration of AZD1775. This highlights that cells may be poised to express preBCR+/BCL6+ upon drug-induced stress (supported by GRO-seq enhancer analysis) and the persisters retain the expression over extended time period, which could indicate switch to metastable (attractor) state that slowly returns to initial state. Since the pre-BCR+/BCL6+ was not detected when challenged by the other drugs (new data added to Fig. S7), a barrier may exist making this transition highly unlikely under basal or “general” stress state. The AZD1775-specific change in the gene regulatory network is therefore likely to be linked to the concomitant loss of RUNX1, MYC and GATA2. We have revised the Discussion to address this point.*

*As the cellular response to WEE1 inhibition is a dynamic and transient process, this complicates a direct assessment of the drug tolerant subpopulation. We appreciate the reviewer's recognition of the challenge in identifying a minor pre-existing subpopulation (presumably representing a cell-fate-III profile marked by pre-BCR/BCL6). This would require the utilization of barcoding technology and meticulous kinetic tracking of cells exposed to the drug and following drug withdrawal. This, however, extends beyond our current research scope. Consequently, we have now moderated our statement, acknowledging that the drug-induced transition to cell fate III does not preclude that the observed drug-induced tolerance and cell survival could pre-exist.*

- Authors should use a PDX-mouse model to test the efficacy of the sequential therapy strategy proposed in vivo.

*Response: In response to the reviewer’s valuable recommendation, we have augmented the revised manuscript with new in vivo data. To focus on the cellular mechanisms of drug effects that we have characterized in detail in the manuscript, we chose a comparable time window as analysed in vitro, collecting cells at day 5. We applied a sequential treatment regime to mice engrafted with two different KMT2A-r cells, from the MLL-7 PDX (matching previous in vivo data from shorter time point) and including RS4;11 cells that we profiled in detail in vitro, as schematically outlined in new Fig 7D. These new results have been included in revised Figures 7, Fig. S8 and Fig S10.*

*Post-treatment, we performed single cell analysis (flow cytometric immunophenotyping, immunofluorescence analysis and scRNA-seq) on CD19+CD45+ human leukemic cells from bone marrow samples. Additionally, we assessed white blood cell counts (WBC), spleen weight, and bone marrow blast percentage that indicated a decrease in leukemia burden.*

*We present the following findings from detailed molecular level analysis of post-treatment cells supporting treatment efficacy in the revised manuscript:*

*i) Decrease in stemness markers: immunophenotype and scRNA analysis showed a decline in CD34+ cells after treatment in both MLL-7 and RS4;11 cells (Fig 7e, f, g).*

*ii) Increase in cell fate III markers pre-BCR and BCL6 by AZD1775 treatment (Fig. 7i), confirming the treatment effects observed in vitro (Fig 6f).*

*iii) In the presence of Dasatinib, the pre-BCR-positive population decreased in RS4:11 (AZD1775 26% vs AZD1775 +Dasatinib 11%). Similarly, the proportion of pre-BCR-positive cells decreased in MLL-7 (AZD1775 97 % vs AZD1775 + Dasatinib 89%), and a distinct population of pre-BCR-negative cells emerged (3% vs 11%) (Fig. 7j).*

*iv) DAPI staining and scRNA-seq results indicated a reduction in actively cycling cells in the MLL-7 model and decreased S-phase population in both xenograft models in the combination treatment (Fig 7k, Fig. S10i-h).*

*v) In both models the scRNA-seq analysis revealed a decrease in the number of expressed genes post-combination treatment (Fig. S10d-f). We refer the reader to Duncan-Lewis et al. 2021 Cytoplasmic mRNA decay represses RNA polymerase II transcription during early apoptosis (https://elifesciences.org/articles/58342) and Thomas et al. 2015 Apoptosis Triggers Specific, Rapid, and Global mRNA Decay with 3′ Uridylated Intermediates Degraded by DIS3L2 (https://doi.org/10.1016/j.celrep.2015.04.026) studies reporting effects on cellular mRNA content at early apoptosis. We took measures to ascertain that this phenomenon was not attributable to poor cell viability due to cell handling prior to measuring in two ways. Firstly, there was no significant difference in the viability stain observed in FACS prior to loading the cells to 10x Chromium for scRNA-seq library preparation, and these cells exhibited a low mitochondrial gene percentage (usually damaged cells have low mitochondrial gene percentage together with low mRNA count). This putative early apoptotic gene expression signature in RS4;11 corresponded to G2/M or G2/M-to-G1 transitioning cells, which were depleted by treatment in MLL-7, as indicated by arrows on UMAP plots (Fig S10h).*

*vi) Subsequent Annexin V/PI staining on CD45+/CD19+ RS4;11 and MLL-7 cells confirmed increased cell death in both xenograft models post-treatment (Fig S10j). This suggests that the observed effects may stem from cell death during the S and G2/M phases, arrest in the G1 or mitotic phase, or a combination thereof.*

*We hope that this new in vivo data upon drug treatment can represent a valuable resource also for future studies that aim to relate in vitro/ex vivo drug responses to in vivo effects, or would proceed to optimise the drug scheduling following the sequential regime proposed here. In our analysis we noticed that the in vivo microenvironment impacted the basal cell state diversity in the RS4;11 xenograft, exhibiting a cell population that was not detected in vitro (p2 in Fig. 7f) that represented more stem-like cells with higher CD34 levels. Notably, these cells were reduced by the sequential treatment. Notably, the effects of WEE1 inhibition on the stemness phenotype could be highly relevant in the clinical setting since such stem-like cells have been shown to have more drug-tolerance in previous studies using standard chemotherapy in mouse models or primary patient data (Ebinger 2016, Chen et al. 2022).*

- Molecular mechanisms underlying cell state diversification upon WEE1 inhibition should be further discussed. Other authors (i.e. Gupta et al., Science Advances 2022- https://www.science.org/doi/10.1126/sciadv.abn4886 (MLL-AF9)) have also reported acceleration in cellular diversification in response to stress or injury in a KMT2A-rearranged leukemia system. Despite the authors describe such effect upon WEE1 inhibition affecting RUNX1 protein levels, a more general mechanism supporting a limited capacity of KMT2A-r cells to cope with stress can be also considered. At least, authors should address these comments in the discussion section.

*Response: We value the reviewer’s question concerning the resilience of KMT2A-r leukemia under stress. In the original manuscript, we discussed the possibility that stress and DNA damage induced by AZD1775 might expose a vulnerability in KMT2A-r cells. Given that KMT2A-r leukemias exhibit a compromised ATR-mediated S/G2-phase checkpoint, they may be particularly susceptible to treatments that provoke replication stress and DNA damage, such as WEE1 inhibitors. This susceptibility could lead to an expedited evolution of cell states in KMT2A-r cells, rapidly engaging pre-survival pathways that culminate in a drug-tolerant, differentiated cell state, as reported here.*

*KMT2A-r leukemias exhibit limited genetic diversity but harbor changes in epigenetic regulation that may make cells more poised to cell state transitions. Gupta et al. found that stress conditions induce increased transcriptional variability and rapid cellular diversification in these cells, indicating a potential role for transcriptional variability in driving leukemia cell fate changes. However, we did not detect this diversification (presence of pre-BCR+/BCL6+ subpopulation) upon other common chemotherapy drugs. Since the study of Gupta et al. is highly relevant in the context of non-genetic evolution, we have revised the Discussion addressing also this aspect.*

*As discussed in the manuscript (and above), our results support a molecular mechanism underlying the rapid diversification of cell states observed upon WEE1 inhibition that could potentially be linked to the downregulation of RUNX1 (and MYC). RUNX1, in particular, occupies a central and indispensable position with high stress centrality within the KMT2A-AF4 gene regulatory network (Harman et al.). Consequently, the downregulation of RUNX1 has the potential to disrupt this central node, redirecting signaling through alternative genes. This perturbation may thus facilitate cell state transitions in KMT2A-r leukemias.*

*Harman JR, Thorne R, Jamilly M, Tapia M, Crump NT, Rice S, Beveridge R, Morrissey E, de Bruijn M, Roberts I, et al: A KMT2A-AFF1 gene regulatory network highlights the role of core transcription factors and reveals the regulatory logic of key downstream target genes. Genome Res 2021, 31:1159-1173.*

Minor:

- Figure 1 and related. Authors claim "Acute drug treatment induced apoptosis as measured by activation of caspase-3/7" but data is not shown

*Response: In the initial manuscript version, we presented caspase results in Fig S6g alongside senescence data; however, we overlooked including a reference to the figure. In the revised version, the caspase results are included as Fig S1c.*

- Recovery experiments: normalization of data should be further detailed. Which is the reference value for % of cell viability calculations?

*Response: This has been clarified in the Methods section.*

- Any hints of how WEE1 expression is upregulated in KMT2A-r ALLs vs non-KMTA2-r cases?

*Response: This is a relevant question. Upregulation of WEE1 in KMT2A-r cases may involve genetic, epigenetic, or signaling pathway alterations that promote WEE1 expression. Since the regulation of WEE1 expression is intricately tied to the DNA damage response and activation of major sensors of DNA replication stress (eg ATR-CHEK1), and a previous study indicate that the replication stress response is a vulnerability in MLL-r AML (Morgado-Palacin et al, Sci Signal. 2016 Sep 13;9(445):ra91. doi: 10.1126/scisignal.aad8243), it is possible that KMT2A-r leukemias manage the excessive replication stress and DNA damage by indirectly activating transcription factors that promote WEE1 gene expression. It would be interesting to examine if KMT2A fusions directly or indirectly influence WEE1 expression at the transcriptional and/or epigenetic level in future studies.*

- Differentiation vs stemness status of the surviving cells upon treatment is not evaluated

*Response: See above, CD34 protein level analysis vs preBCR/BCL6 has been added to the revised manuscript.*

*We thank this Reviewer for their insightful and constructive suggestions to improve our manuscript.*

**Second round of review**

**Reviewer 1**

The authors have answered my questions. I have no more comments.
